# Homeodomain proteins: an update

**DOI:** 10.1007/s00412-015-0543-8

**Published:** 2015-10-13

**Authors:** Thomas R. Bürglin, Markus Affolter

**Affiliations:** Biozentrum, University of Basel, Klingelbergstrasse 50/70, 4056 Basel, Switzerland; Department of Biomedicine, University of Basel, Mattenstrasse 28, 4058 Basel, Switzerland

**Keywords:** Homeobox, Homeodomain, Hox, PAIRED (PRD) domain, EH1 (Octapeptide/TN) motif, DNA binding

## Abstract

Here, we provide an update of our review on homeobox genes that we wrote together with Walter Gehring in 1994. Since then, comprehensive surveys of homeobox genes have become possible due to genome sequencing projects. Using the 103 *Drosophila* homeobox genes as example, we present an updated classification. In animals, there are 16 major classes, ANTP, PRD, PRD-LIKE, POU, HNF, CUT (with four subclasses: ONECUT, CUX, SATB, and CMP), LIM, ZF, CERS, PROS, SIX/SO, plus the TALE superclass with the classes IRO, MKX, TGIF, PBC, and MEIS. In plants, there are 11 major classes, i.e., HD-ZIP (with four subclasses: I to IV), WOX, NDX, PHD, PLINC, LD, DDT, SAWADEE, PINTOX, and the two TALE classes KNOX and BEL. Most of these classes encode additional domains apart from the homeodomain. Numerous insights have been obtained in the last two decades into how homeodomain proteins bind to DNA and increase their specificity by interacting with other proteins to regulate cell- and tissue-specific gene expression. Not only protein-DNA base pair contacts are important for proper target selection; recent experiments also reveal that the shape of the DNA plays a role in specificity. Using selected examples, we highlight different mechanisms of homeodomain protein-DNA interaction. The PRD class of homeobox genes was of special interest to Walter Gehring in the last two decades. The PRD class comprises six families in *Bilateria*, and tinkers with four different motifs, i.e., the PAIRED domain, the Groucho-interacting motif EH1 (aka Octapeptide or TN), the homeodomain, and the OAR motif. Homologs of the co-repressor protein Groucho are also present in plants (TOPLESS), where they have been shown to interact with small amphipathic motives (EAR), and in yeast (TUP1), where we find an EH1-like motif in MATα2.

**Electronic supplementary material:**

The online version of this article (doi:10.1007/s00412-015-0543-8) contains supplementary material, which is available to authorized users.

## Introduction

In 1994, we wrote a review on homeodomain (HD) proteins for Annual Review of Biochemistry together with Walter Gehring that became a standard reference (Gehring et al. 1994). Sadly, Walter passed away in May 2014 as a consequence of a tragic traffic accident (Affolter and Müller 2014; Affolter and Wüthrich 2014; Levine 2014; Mlodzik and Halder [Bibr CR178][Bibr CR178]; Mlodzik and Halder [Bibr CR179][Bibr CR179]; Schier 2014; Wieschaus and Nüsslein-Volhard 2014). In his honor, we provide here an update of this review, although we will barely be able to scratch the surface, given that over 17,000 publications containing “homeobox,” “homeodomain,” or “Hox” in the title or abstract have appeared since the first publications. We will highlight a few of the novel findings of the past two decades, with special emphasis on topics that were of particular importance to Walter Gehring.

Our understanding of the homeobox gene family has expanded substantially in the last 20 years, not least because the numerous completed genome sequences allow comprehensive analyses. While many findings and the basic framework from 1994 are still valid, numerous revisions and refinements have been made since then with regard to classification and homeobox gene numbers per individual genome. More structural data has also become available, and the characterization of the molecular roles of HD proteins has tremendously advanced.

The homeobox was originally discovered as a shared sequence element of about 180 bp in homeotic genes in *Drosophila melanogaster*, which gave rise to its name (McGinnis et al. 1984a; Scott and Weiner 1984) (for review, see Bürglin 2013a; Bürglin 2013b; Pick 2015). Soon, it was realized that this motif was also conserved in vertebrates (McGinnis et al. 1984b), and the first vertebrate homeobox gene was cloned from *Xenopus laevis* by Andrés Carrasco and colleagues in the laboratory of Eddy De Robertis (Carrasco et al. 1984), across the hall from the laboratory of Walter Gehring at the Biozentrum in Basel. In a sad coincidence, Andrés Carrasco also passed away in May 2014 (Blumberg 2014).

The homeobox sequence encodes the HD, a globular domain of about 60 amino acids that normally functions as a DNA-binding domain. We now know that in animals, there are usually around 100 homeobox genes in protostome species, e.g., 103 in *Caenorhabditis elegans* (Hench et al. 2015), 103 in *Drosophila melanogaster* (Sup. Fig. S1), 121 in the sea snail *Lottia gigantea* (Simakov et al. 2013), 111 in the polychaete worm *Capitella teleta* (Simakov et al. 2013), and at least 92 in the oyster *Pinctada fucata* (Morino et al. 2013). In the leech *Helobdella robusta*, an expansion has taken place, resulting in 181 homeobox genes (Simakov et al. 2013). In the deuterostome branch, 96 homeobox genes were found in the sea urchin *Strongylocentrotus purpuratus* (Howard-Ashby et al. 2006), and 133 in *Amphioxus branchiostoma* (Takatori et al. 2008). Most vertebrates have about 250 homeobox genes, due to two extra rounds of genome duplication and subsequent loss of paralogs (Holland 2013). In teleost fish, one additional round of genome duplication followed by gene loss increased the number to over 300 (Holland 2013). Overall, about 15–30 % of all transcription factors in animals are HD proteins (de Mendoza et al. 2013), which represents about 0.5–1.25 % of all proteins in a given species. In plants, similar numbers of homeobox genes can be found, e.g., 110 in *Arabidopsis thaliana* (Mukherjee et al. 2009). In fungi and single-cell organisms, the number tends to be small, usually less than a dozen (Derelle et al. 2007), but in *Acanthamoeba*, the homeobox family has expanded to 25 (Clarke et al. 2013). In a number of unicellular eukaryotes, homeobox genes seem to have been lost entirely (de Mendoza et al. 2013; Derelle et al. 2007), though in some cases, e.g., *Paramecium*, they were subsequently found (de Mendoza et al. 2013) (Sup. Fig. S1).

HD transcription factors fulfill a plethora of biological functions. There is probably no tissue in plants or animals that does not require them to function properly. In animals, they act from the earliest stages of development onward (Driever and Nüsslein-Volhard 1988; Töhönen et al. 2015), and they are essential in embryonic stem cells (Young 2011). They play crucial roles in patterning, in particular the Hox genes (Capellini et al. 2011; Kmita and Duboule 2003; Maeda and Karch 2015; Pearson et al. 2005; Rezsohazy et al. 2015; Seifert et al. 2015; Zakany and Duboule 2007). Many are involved in nervous system development (Schulte and Frank 2014; Vollmer and Clerc 1998; Zagozewski et al. 2014), and not surprisingly, disruption of homeobox genes leads to various genetic disorders and diseases (Kumar 2009; Liu et al. 2015; Purkayastha and Roy 2015; Quinonez and Innis 2014; Wang et al. 2014a). Also in plants, homeobox genes regulate numerous aspects of development, e.g., stem cell maintenance, lateral outgrowth, stress response, or light response (Brandt et al. 2014; Costanzo et al. 2014; Hay and Tsiantis 2010; Ratcliffe and Riechmann 2002; Tsuda and Hake 2015). While much progress has been made in understanding the function of many HD proteins, even in the model system *Drosophila* 12 homeobox genes have not yet been subject to intensive study, and therefore, lack a descriptive name associated with their function (Sup. Fig. S1).

## HD sequence and classification

### The HD sequence

**Fig. 1:**
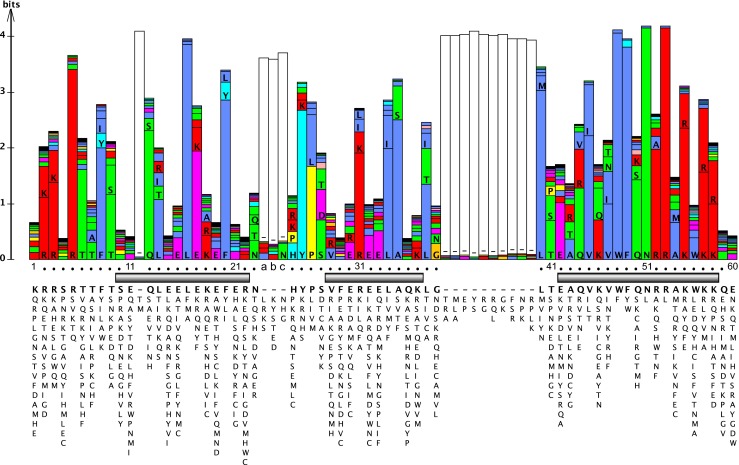
HD protein logo generated primarily from *Drosophila* HD proteins (Sup. Fig. S1) using LogoBar (Pérez-Bercoff Å and Bürglin 2010; Pérez-Bercoff et al. 2006). The higher the bar, the stronger a position is conserved. *Letters inside the bars* indicate amino acid residues. *Open bars* indicate gap regions that were introduced to accommodate longer atypical HDs. *Numbers underneath* are based on the standard numbering for HDs with 60 residues, and “*abc*” marks the positions of the three extra residues in loop 1 of TALE HDs. The three alpha helices are indicated with *shaded boxes*. At the bottom, the consensus sequence (most frequent residue) is shown; residues underneath each position are listed in decreasing order of frequency of occurrence

The “typical” HD is 60 amino acids long. The originally described consensus sequence was biased toward animal homeobox genes, particular of the ANTENNAPEDIA (ANTP) class (Bürglin 1994b; Gehring et al. 1994). As more genomes were sequenced, more divergent HDs were encountered. Here, we created a new profile of the conserved residues (a protein logo) of the HD sequences from a single animal, *Drosophila melanogaster*, including a few selected other HDs (Sup. Fig. S1, Fig. 1). This provides a less biased profile, although still 46 % of the HD sequences are of the ANTP class (Table 1). Compared to the previous profile (Bürglin 1994b; Bürglin 1995; Gehring et al. 1994), the overall pattern of amino acid conservation stays essentially the same. Due to more divergent sequences, individual positions show now more variability than evident in the previous profile (Fig. 1). In *C. elegans* (Hench et al. 2015) and plants (Mukherjee et al. 2009), the HD profiles show even more variability due to higher numbers of divergent HDs.

**Table 1:** Summary of all HD proteins in *Drosophila melanogaster* according to their classification

Superclass	Class	Subclass	Nr.		Pos. 5	Pos. 50
	ANTP	HOXL	19	47	R	Q, 1K
		NKL	26		R, 1Q	Q
			2		R	Q
	PRD		7 (+3)^a^		R	S
	PRD-LIKE		19		R	Q, 3K
	LIM		7		R	Q
	ZF		2		R	Q, 1R
	POU		5		R	C
	HNF		lost		(R)^b^	(A)^b^
	CUT	CUX	1		R	H
		ONECUT	1		R	M
		CMP	1		R	K
		SATB	Not present		(R)^b^	(Q)^b^
	PROS		1		S	S
	CERS		1		S	R
	SIX/SO		3		S, T, V	K
TALE	PBC		1		R	G
	MEIS		1		R	I
	TGIF		2		R	I
	IRO		3		R	A
	MKX		1		K	A
Total			103 (+3)			

The updated classification scheme that we suggest for future use retains only the TALE superclass, which is conserved from animals to plants. Number (Nr.) of proteins within a class/subclass are given. Residues found at position 5 (Pos. 5) and position 50 (Pos. 50) of the HD are indicated in the columns; numbers before residues indicate the number of less frequently found residues

^a^The PRD class contains three additional proteins that lost their HD (i.e., Poxn, Poxm, Sv), which is indicated in brackets and not counted as HD protein proper

^b^Residues of the human sequences HNF1A and SATB1, respectively

Some examples of this additional variability can be found in key residues important for the hydrophobic core of the HD, which constitute the signature of the HD and seemed to be essentially invariant, i.e., leucine (L,16), phenylalanine (F, 20), tryptophan (W, 48), and phenylalanine (F, 49). However, they can be substituted with amino acids of similar properties. For example, instead of the core signature sequence WF (pos. 48, 49), position 48 can be, e.g., phenylalanine (F), or tyrosine (Y), while position 49 can be occupied by tyrosine (Y), tryptophan (W), or small hydrophobic residues such as methionine (M), isoleucine (I), or leucine (L). Another important residue is the basic residue arginine (R) at position 5 of the HD (Sup. Fig. S1), which is found in 99 of the 106 *Drosophila* HDs.

### Updates to HD classes

**Fig. 2:**
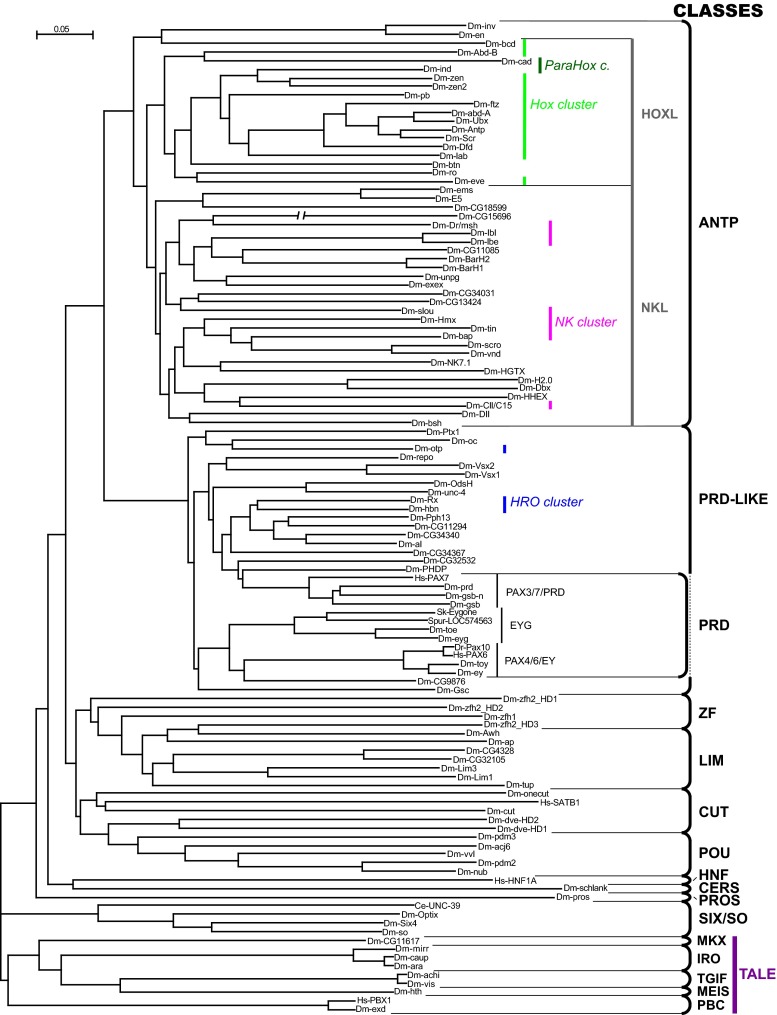
Classification of HD proteins. A phylogenetic tree of the HD sequences in Sup. Fig. S1 was created using neighbor joining in Clustal X (Larkin et al. 2007). Classification of the HDs is marked on the *right*. Genes belonging to genomic clusters are marked in *different colors in italics*. The three PRD class families with HDs are indicated. Due to the limited number of sequences, the tree should only be taken as simple guide to illustrate how similar or divergent HD sequences are when compared to each other. Hence, not all genes within a class fall within the same clade

**Fig. 3:**
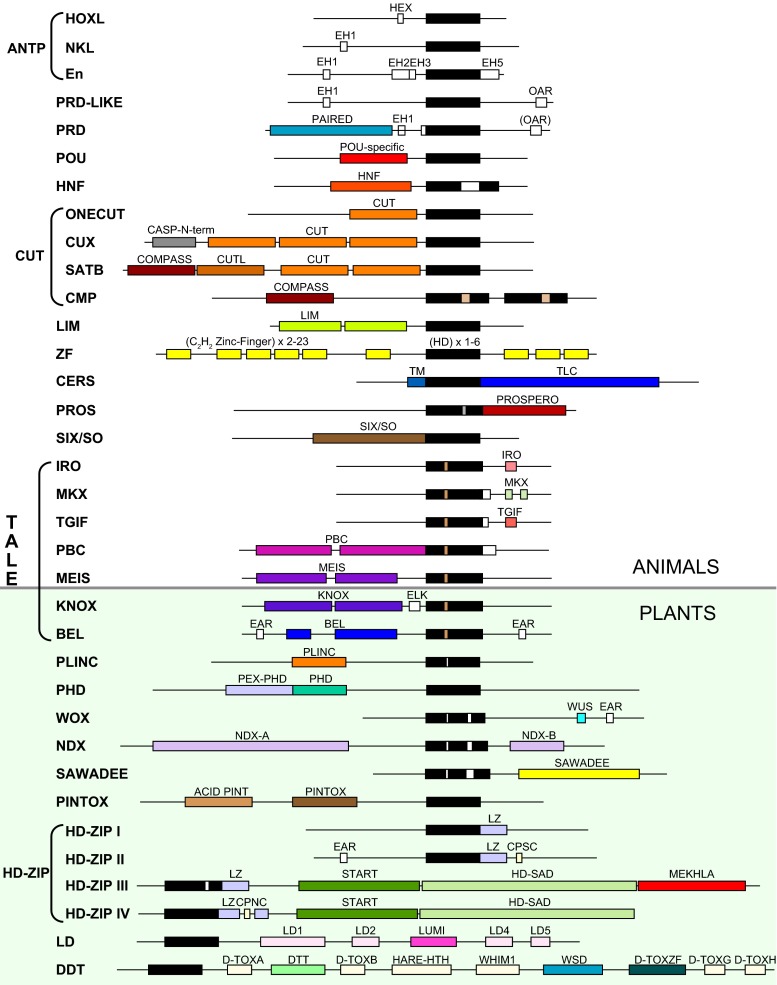
Schematic representation of conserved domains and motifs associated with HD proteins in different classes. The upper part shows classes of animal HD proteins, the lower part shows classes of plant HD proteins. Not all domains and motifs are shown, which is mostly the case for motives conserved only within families

The principles underlying the classification of HDs have been outlined previously (Bürglin 2005; Bürglin 2011; Holland 2013). Briefly, in the case of animals, orthologous homeobox genes that can be traced at least to the urbilaterian split are placed into families. Families with similar features (e.g., a particular additional domain) are grouped together into classes. Classes may be merged into a superclass or subdivided into subclasses. Such a simplistic system, however, does not fully reflect the real evolutionary complexity. New homeobox genes can arise by duplication and may diverge substantially from the precursor in a relatively short time frame, giving rise to new families that may be restricted to a single taxonomic class. The most important aspect of a good and consistently used classification is that orthologous genes in different species are properly identified and not confused with paralogs. The updated classification presented here is based on two criteria: on the one hand, the sequence similarity of HDs to each other, which is used to generate phylogenetic trees (Fig. 2); on the other hand, flanking conserved domains and motifs in the HD proteins can be used as classifiers (Fig. 3). Here, we suggest retaining only one superclass, TALE, because of its deep evolutionary conservation (Figs. 2 and 3).

The HD is embedded in proteins that can differ substantially in size. Some proteins are barely larger than the HD itself, e.g., mouse Hopx (73 amino acids) (Kook et al. 2006) or *C. elegans* CEH-7 (84 amino acids) (Kagoshima et al. 1999), while some are large and contain many other domains, e.g., *Arabidopsis* Ringlet 1 (AT1G28420, 1705 amino acids), or human ZFHX3 (ATBF1, 3703 amino acids, Sup. Fig. S3). Figure 3 shows the different domains and some of the smaller motifs found in various classes of homeobox genes. In addition to the major domains, smaller motifs or regions can be conserved within families, or even between related families, many of which are not shown in Fig. 3. For example, the very N-terminal region showed sequence conservation between the proteins of several different Hox families (De Robertis et al. 1988). In *Drosophila*, it was demonstrated that the core of this region with the residues “SSYF” is an activation domain in Ubx and Scr proteins in *Drosophila* (Tour et al. 2005).

Many classes of homeobox genes in animals were already discovered in 1994 (Bürglin 1994b; Gehring et al. 1994). However, a number of new classes have appeared, and some refinement and re-evaluation has taken place. Furthermore, most of the plant homeobox gene classes were discovered only after 1994. We will discuss these new findings below.

Overall, given that numerous complete genome sequences are now available, the classification of HD proteins for bilaterians and vascular plants is probably quite complete. More information and analysis of “lower” eukaryotes may reveal novel types of HD proteins that evolved in specific branches. E.g., in *Dictyostelium* a new group of double homeobox genes has evolved (Clarke et al. 2013). Also, within particular phyla, new types of HD proteins may evolve. For example, in the genus *Caenorhabditis* a novel, highly divergent type of double HD emerged, termed HOCHOB (Hench et al. 2015).

Using the model system *Drosophila melanogaster* as an example to summarize the complement of homeobox genes in a single organism (Fig. 2, Sup. Fig. S1) we find that a large fraction is made up of ANTP (46 %) and PRD-LIKE (18 %) homeobox genes (Table 1). The remaining fraction (36 %) is shared by the remaining classes, which all encode large domains flanking the HD. Some homeobox genes were lost in the evolutionary lineage leading to *Drosophila*, e.g., the HNF class and the Prep family (MEIS class) are missing (Chi et al. 2002; Mukherjee and Bürglin 2007).

### Animal HD classes and motifs

Most of the animal HD classes emerged early in metazoan evolution. A number are already present in sponges, while most exist in *Placozoa*, *Cnidaria*, and *Ctenophora* (Ryan et al. 2006; Ryan et al. 2007; Ryan et al. 2010; Srivastava et al. 2008; Srivastava et al. 2010b). In this section, we give brief updates regarding the different animal HD classes, except for PRD, SIX/SO, and POU, which are dealt with further below.

#### ANTENNAPEDIA (ANTP) class

The ANTP class can be broadly divided into two subclasses. The HOXL (Hox-like) group encompasses genes most similar to the Hox genes (Fig. 2), i.e., those genes found in the Hox cluster. According to established guidelines, only homeobox genes in the Hox cluster should be named Hox genes (or genes derived from the Hox cluster, if this has secondarily broken up). Many of the HOXL genes have a short motif, the Hexapeptide (HEX, aka YPWM), upstream of the HD (Fig. 3). In the AbdB family this motif has diverged, although a tryptophan (W) is still present. The NKL (NK-like) group is comprised of the NK type homeobox genes, a number of which are found in the NK cluster (see below). A few NKL genes also encode a HEX motif (e.g., Tlx). In many NKL families, an EH1 motif is found toward the N-terminus (see below). Some genes, such as *Drosophila engrailed* (*en*), cannot be easily assigned to either subclass. The distinction into HOXL and NKL is not always clear-cut, due to the fact that HOXL genes are probably derived from NKL genes (see below).

#### The Octapeptide/Hep/EH1/TN/GEH motif, a Groucho interaction motif

**Fig. 4:**
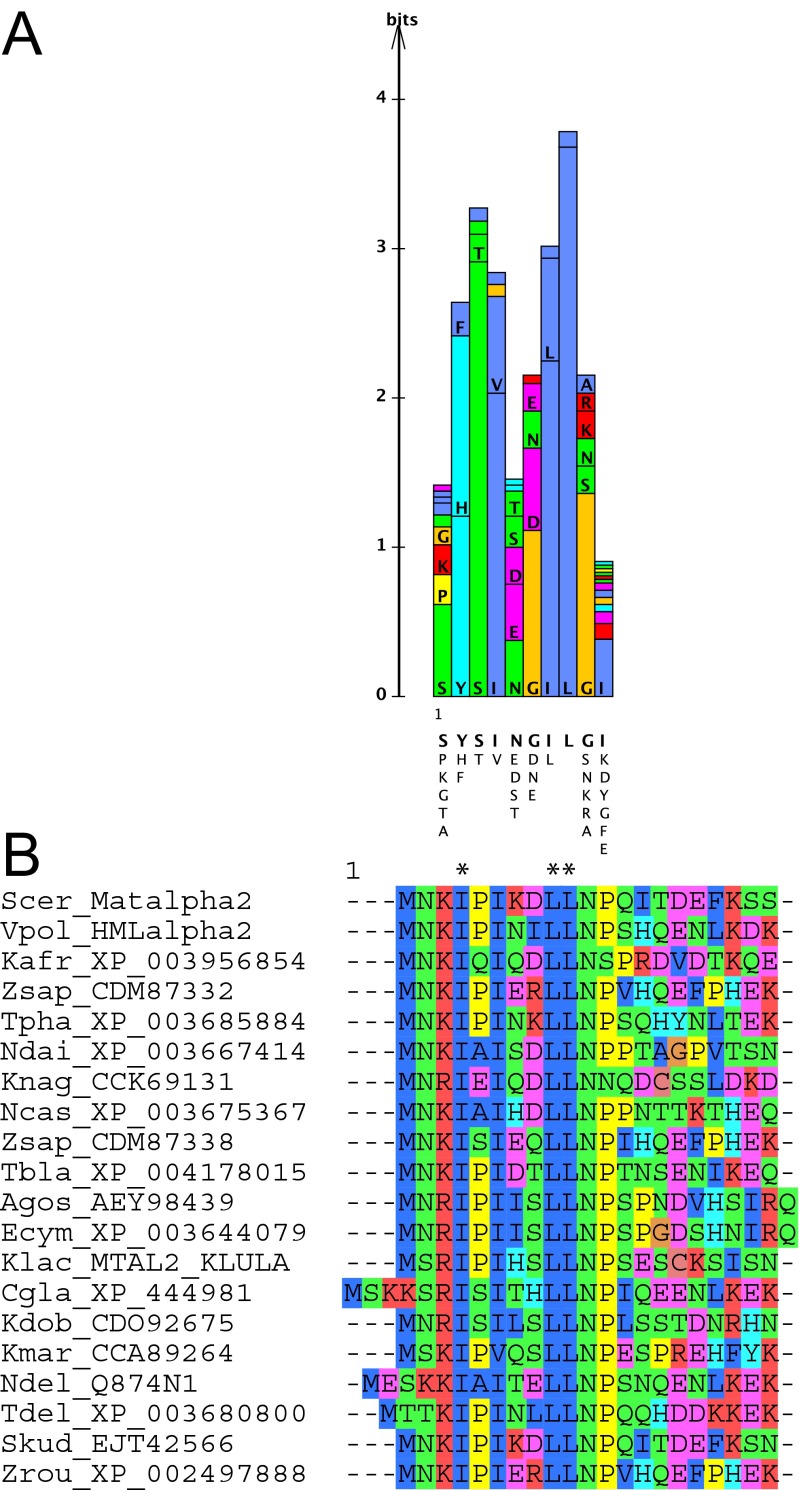
Gro and TUP1 interaction motifs. **a** Protein logo of the EH1/Octapeptide Gro interaction motif derived from PRD class proteins. The EH1 motif from the PRD class protein alignment (Sup. Fig. S5) was taken and a logo created using LogoBar (Pérez-Bercoff et al. 2006). Underneath the logo the most common residues in descending order are shown. **b** Conserved N-terminal region of fungal MATα2 HD proteins (for the complete alignment see Sup. Fig. S2). Asterisks mark positions were mutations in MATα2 were isolated in a TUP1 interaction screen (Komachi et al. 1994). Note the matching pattern of hydrophobic residues to the EH1 motif

The Octapeptide was first discovered in PAIRED domain-containing proteins of fly and humans as a short, conserved sequence motif between the PAIRED domain and the HD (Burri et al. 1989; Noll 1993). Subsequently, variants of this motif were discovered in other HD proteins, and given different names, i.e. Hep (Allen et al. 1991), EH1 (in En, Hemmati-Brivanlou et al. 1991; Hui et al. 1992; Joyner and Hanks 1991; Logan et al. 1992), TN (in NK/Tinman proteins, Bodmer 1995; Lints et al. 1993), and GEH (Goriely et al. 1996). The common similarity was not always noted, and also its significance was unclear, due to the shortness of the motif. However, as more sequences became available, the motif was better defined (Harvey 1996; Smith and Jaynes 1996), and eventually was found to occur in many PRD-LIKE, PRD, and NKL class HD proteins, as well as other transcription factors, such as Fox, Ets, and T-Box (Copley 2005; Shimeld 1997; Yaklichkin et al. 2007). We refer to the motif from here on as EH1, the most commonly used name (Copley 2005; Yaklichkin et al. 2007). The core of the motif spans seven residues with only a few conserved positions. Even when only the EH1 motifs encoded by the PRD class proteins are compared, the limited conservation of the motif can be noted (Fig. 4a, Sup. Fig. S5). Since the motif is so small, convergent evolution cannot be excluded. In the PRD-LIKE HD protein UNC-4 of *C. elegans*, the EH1 motif is found C-terminal to the HD, which implies either a duplication event, or a de novo origin of the motif at this position (Winnier et al. 1999). Nevertheless, the fact that this motif has been well conserved in many homeobox families across the bilaterian divide, and even in *Cnidaria*, suggests that this motif has been subject to strong evolutionary constraint. We would like to suggest that the common ancestor of ANTP, PRD, and PRD-LIKE homeobox genes already encoded this motif.

*Drosophila* En is a transcriptional repressor protein. Functional mapping experiments revealed that an important motif that conveys the repressor activity is EH1 (Smith and Jaynes 1996). Further experiments revealed that EH1 binds to the Groucho (Gro) co-repressor protein to exert its repressor function (Fisher and Caudy 1998; Jiménez et al. 1997; Jiménez et al. 1999; Muhr et al. 2001; Papizan et al. 2011; Winnier et al. 1999). EH1 in other proteins was also confirmed to interact with Gro as in the case for the zinc-finger factor Odd-skipped (Goldstein et al. 2005). Gro is a co-repressor that works together with many developmental transcription factors (Jennings and Ish-Horowicz 2008; Mannervik 2014). Gro and its human orthologs TLE (Transducin-like Enhancer of split) are characterized by an N-terminal glutamine-rich domain and a conserved WD-repeat. Gro/TLE not only recognizes the EH1 motif, but also another motif, termed WRPW based on its sequence. The WRPW motif is found in factors such as Hairy or Runt (Chen and Courey 2000; Cinnamon and Paroush 2008; Turki-Judeh and Courey 2012). Structural studies have shown that the EH1 and the WRPW motif bind into the central groove of the beta propeller structure of the WD repeats of TLE1 (Jennings et al. 2006). The two motifs bind on top of the mouth of the central channel and EH1 forms a short amphipathic alpha helix that binds to this hydrophobic recess.

In yeast, the Gro homologous factor is the TUP1 protein that also functions as a transcriptional repressor (Smith and Johnson 2000). In a genetic screen to identify mutations of yeast MATα2 defective in repression, a number of point mutations were isolated, many of which mapped to the N-terminus of MATα2 (positions 4, 9, 10, Fig. 4b) (Komachi et al. 1994). We observed that the N-terminus is well conserved in many fungal MATα2 proteins, in particular in those positions that, when mutated, relieve repression (Fig. 4b, Sup. Fig. 2S). These positions are characterized by small hydrophobic residues with the same spacing as in the EH1 motif. The only exception is that an isoleucine (I) residue is present instead of a F/Y/H residue at the first hydrophobic position. Thus, also in yeast, a slightly modified version of the EH1 motif exists that interacts with a Gro family molecule, i.e., TUP1.

Gro/TLE has been proposed to function via its interaction with histone deacetylases (HDAC) and subsequent chromatin modification, and exert long-range repression through oligomerization (Turki-Judeh and Courey 2012). More recent evidence suggests alternative pathways. For example, in yeast, it has been found that rapid depletion of the Cyc8-Tup1 co-repressor results in de-repression of target genes, while re-association of Tup1 leads to rapid repression before any repressive chromatin structure can be formed (Wong and Struhl 2011). Co-activators such as Swi/Snf, SAGA, and mediator complex can be rapidly recruited upon TUP1 depletion. Thus, it is thought that TUP1 repression acts primarily through masking of activation domains in transcription factors. In *Drosophila*, Gro is found at transcription start sites containing hypoacetylated histones H3 and H4, and at sites that exhibit strong RNA polymerase pausing. Activation and repression responses can be very rapid in vivo, also suggesting that Gro/TLE modulates transcription, rather than cause general chromatin repression (Kaul et al. 2014). Recently, it has been demonstrated that the Mediator subunit Med19 can bind directly to the HD of Hox proteins (Boube et al. 2014). Thus, perhaps, and similar to yeast, Gro/TLE blocks recruitment of mediator to the HD by binding to the EH1 motif.

#### PAIRED-LIKE (PRD-LIKE) class

We consider the PAIRED class (see below) separately from the PRD-LIKE class, while others group them together (e.g., Zhong and Holland 2011a). The PRD-LIKE class genes encode only the HD as the major conserved domain. Like the NKL genes, about 10 PRD-LIKE families encode an EH1 motif toward the N-terminus (Vorobyov and Horst 2006). A second small motif found in over half of the PRD-LIKE proteins is the OAR motif, first identified in *otp*, *al*, and *rax* (Furukawa et al. 1997). The OAR motif is encoded near the C-terminus of ten families of PRD-LIKE homeobox genes (Galliot et al. 1999; Vorobyov and Horst 2006). The OAR motif is thought to play a role in transcriptional activation (Vorobyov and Horst 2006).

#### TALE superclass

The typical HD has 60 residues that fold into a globular structure with three alpha helices connected by two short loops (Bürglin 1994b; Gehring et al. 1994). HDs with deviations from this length have been characterized as “atypical” and usually accommodate extra residues either in loop 1 between helices 1 and 2, and/or in loop 2, between helices 2 and 3 (Bürglin 2005; Bürglin 2011). “Atypical” proved not to be a useful classification characteristic, since the insertion (or deletion) of extra residues has occurred multiple times independently in evolution. For example, even in the well-conserved SIX/SO class, the *C. elegans* gene *unc-39* (*ceh-35*) encodes an extra residue in loop 1 (Dozier et al. 2001) (Sup. Fig. S1). However, one special group of HD proteins, the TALE superclass, is characterized by a HD with 63 residues, where three extra residues are inserted in loop 1 (Bertolino et al. 1995; Bürglin 1995; Bürglin 1997) (Sup. Fig. S1). The TALE HD proteins are highly conserved in evolution and are present in single-cell eukaryotes, in plants, and in animals in parallel with typical homeobox genes, and therefore represent an ancient split into two types of HD proteins (Bharathan et al. 1997; Bürglin 1997; Bürglin 1998; Derelle et al. 2007). In plants, the TALE proteins can be divided into two classes, BEL and KNOX (Mukherjee et al. 2009). The KNOX and BEL factors have been shown to heterodimerize (Bellaoui et al. 2001; Lee et al. 2008). In animals, the TALE group has split into five classes (PBC, MEIS, IRO, MKX, TGIF) with different domain configurations (Fig. 3). One of these classes, MEIS, is further subdivided into two families (MEIS and PREP) (Bürglin 1997; Mukherjee and Bürglin 2007). Both PBC and MEIS HD proteins, including upstream conserved domains, are already present in *Acanthamoeba*, which does indicate an ancient role for these TALE HD proteins (Clarke et al. 2013).

#### CUT class

The CUT class has been divided into several subclasses based on the associated domains, i.e., CUX (comprising the *Drosophila cut* gene), ONECUT, SATB, and COMPASS (CMP) (Bürglin and Cassata 2002; Takatori and Saiga 2008). Proteins of the CUX, ONECUT, and SATB classes encode one to three copies of the about 80 residue-long CUT domain (Fig. 3). The crystal structure of the CUT domain has been determined and found to comprise five main alpha helices, with helix 3 binding in the major groove of the DNA, and it showed structural similarity to the POU-specific domain (Iyaguchi et al. 2007; Yamasaki et al. 2007). In the Cux family, the N-terminal region (though not in *Drosophila*) is part of an alternative spliced product called CASP (CDP/CUX alternatively spliced cDNA), which is found in yeast and plants as a distinct, separate protein (Bürglin and Cassata 2002; Gillingham et al. 2002). CASP localizes to the Golgi and its N-terminal region is predicted to adopt a coiled-coil structure (Gillingham et al. 2002; Malsam et al. 2005), which might be used in the Cux family for protein-protein interaction.

The CMP genes do not encode CUT domains. Instead, their association with CUT class genes is solely based on the N-terminal COMPASS domain that is found both in the CMP proteins (e.g., *Drosophila* Dve) and in vertebrate SATB proteins. Analysis of the crystal structure of the COMPASS domain showed that it has an ubiquitin-like structure and can form tetramers (Wang et al. 2012; Wang et al. 2014b). Oligomerization is essential for SATB proteins to exert their function when binding to matrix attachment regions (MARs). In SATB proteins, a DNA-binding CUT-LIKE domain follows the COMPASS domain (Wang et al. 2014b).

#### HNF class

The mammalian HNF1A (LFB1) transcription factor was initially described as an atypical HD protein with a long insert in loop 2 of the HD (Finney 1990; Frain et al. 1989) (Sup. Fig. S1). Subsequently, orthologs in invertebrate species were discovered and a conserved domain (HNF domain) of about 90 amino acids was found upstream of the HD. Structural analysis of HNF1A revealed that the HNF domain is comprised of five alpha helices, with helices 2 to 4 showing structural similarity to the POU domain (Chi et al. 2002). It is thought that the HNF class diverged from the POU class of homeobox genes.

#### LIM class

LIM class HD proteins encode two LIM domains upstream of the HD (Bürglin 1994b). The LIM domain is about 50–60 residues long and is comprised of a double zinc finger motif with a predominant consensus of CX_2_CX_16–23_HX_2_CX_2_CX_2_ CX_16–21_CX_2_(C/H/D) (Kadrmas and Beckerle 2004). The LIM domain is involved primarily in protein-protein interaction (Kadrmas and Beckerle 2004; Zheng and Zhao 2007). Six families have been defined in bilaterians (Hobert and Westphal 2000; Srivastava et al. 2010a). A closely related family, LMO (aka rhombotin), encodes only two LIM domains, possibly having lost the HD secondarily (Boehm et al. 1991; Srivastava et al. 2010a). Unlike many other HD-associated motifs (e.g., PRD, POU, CUT), the LIM domain occurs in many other protein classes, varying in number between 1 to 6 copies, and associating with numerous other domains and motifs; overall, 14 LIM classes have been defined, many of which are involved in cytoskeletal function (Kadrmas and Beckerle 2004; Koch et al. 2012; Te Velthuis et al. 2007).

#### ZF class

Zinc finger (ZF) class homeobox genes encode C_2_H_2_ and C_2_H_2_-like zinc fingers (ZF) in addition to the HD. C_2_H_2_ zinc fingers are typically involved in DNA binding (Najafabadi et al. 2015). The number of zinc fingers (2–23) as well as the number of the HDs (1–6) can vary substantially, e.g., human ZFHX3 (aka ATBF1) has 23 ZFs and 4 HDs (Sup. Fig. S3). In vertebrates, five families were defined (Adnp, Tshz, Zeb, Zfhx, Zhx) (Holland et al. 2007). Three families, Zeb (*Drosophila zfh-1*), Zfhx (*Drosophila zfh-2*), and Tshz are conserved across the bilaterian divide. The vertebrate Tshz family members encode a divergent HD, but the two Tshz paralogs in *Drosophila* (*teashirt* and *tiptop*) lack the HD (Koebernick et al. 2006; Santos et al. 2010); this probably represents a secondary loss of the HD. The Adnp and Zhx families seem to be vertebrate specific. The *Homez* gene is derived from the Zhx family (Bayarsaihan et al. 2003), even though it does not encode ZFs, which most likely were lost secondarily. In fungi, C_2_H_2_-HD proteins have also been identified, although their relationship to the Metazoan ZF-HD has not been systematically investigated (Xiong et al. 2015).

#### PROSPERO (PROS) class

The PROS homeobox genes encode a highly divergent HD with extra residues in loop 2 (Sup. Fig. S1). They also lack the usual basic residues at the N-terminus of the HD. C-terminal to the HD is the 100 amino acids long PROSPERO domain (Bürglin 1994a). X-ray structure analyses revealed a continuity between the HD and the PROSPERO domain; the third alpha helix of the HD is extended, and, together with three further alpha helices, a four-helix bundle is formed that could contribute to DNA binding (Ryter et al. 2002).

#### CERS (aka LASS) class

In most studied cases, HD proteins act as transcription factors. However, in the CERS (aka longevity assurance (LASS)) class, the HD is embedded in a protein carrying multiple transmembrane (TM) regions, where the TM regions following the HD constitute a TLC (TRAM, LAG1, CLN8) domain (Mesika et al. 2007; Mizutani et al. 2005; Pewzner-Jung et al. 2006). The CERS genes encode ceramide synthases, and the HD does not appear to be essential for this function. Not all CERS genes encode a HD, suggesting that the HD may have been acquired in a translocation event at some point in evolution. Experimentally, virtually the complete HD could be deleted without affecting function, although residues at the very end of the HD and in the linker between the HD and the second TM region are required for function (Mesika et al. 2007). In a 1-hybrid system, the isolated HD was shown to be able to bind DNA, suggesting that it did not lose its DNA-binding capacity (Noyes et al. 2008).

### HD genes in plants

#### Plant HD classes

In plants, the HD proteins can be divided into 11 classes (HD-ZIP, BEL, KNOX, WOX, DDT, PLINC, PHD, NDX, SAWADEE, PINTOX, and LD) based on the associated domains (Mukherjee et al. 2009; Viola and Gonzalez 2015) (Fig. 3). One of these classes, HD-ZIP, is divided into four related subclasses (HD-ZIP I to IV), since their members all encode leucine zippers following the HD. HD-ZIP III and IV both contain a START and a HD-SAD (HD-START associated domain) (Mukherjee et al. 2009; Schrick et al. 2004); the START domain has been implicated in lipid/sterol binding (Alpy and Tomasetto 2014; Schrick et al. 2014). The HD-ZIP III class, in addition, has a MEKHLA domain at the C-terminus. This domain is related to PAS domains and regulates dimerization, and thereby transcriptional activity, of the HD protein, via some cell intrinsic signal or mechanism (Duclercq et al. 2011; Magnani and Barton 2011; Mukherjee and Bürglin 2006). Two subclasses, HD-ZIP II and HD-ZIP IV, encode a CxxC motif downstream of, or within the leucine zipper (hence aka zipper-loop-zipper (ZLZ) motif), respectively (Ciarbelli et al. 2008; Nakamura et al. 2006). It has been suggested that intracellular redox state can influence the activity of these factors via these cysteine motifs (Tron et al. 2002).

The DDT class is characterized by a DDT domain, which is found in numerous other factors involved in chromatin regulation, and binds to the SLIDE domain in ISWI chromatin remodeling factors (Doerks et al. 2001; Dong et al. 2013). DDT class HD proteins contain additional conserved domains named D-TOXA to D-TOXH, and WSD, named because of the sequence conservation to BAZ/Williams syndrome transcription factor (WSTF) chromodomain proteins (Mukherjee et al. 2009). The bipartite WSD domain was already described in the BAZ proteins as BAZ1 and BAZ2 motifs (Jones et al. 2000). Recently, D-TOXC was characterized as a winged helix-turn-helix domain, named HARE-HTH, which is found in eukaryotic and prokaryotic proteins involved in DNA binding or modification (Aravind and Iyer 2012). Further, D-TOXD and WSD (BAZ1 and BAZ2) were named WHIM1, WHIM2, and WHIM3 and were also implicated in the interaction with ISWI factors (Aravind and Iyer 2012).

Several classes have distinctive domains with conserved cysteine/histidine residues, that are putative or confirmed zinc fingers, i.e., the PLINC (plant zinc finger) “double finger” (two motifs C-X_3_-H-X_9_-D-X-C and C-X_2_-C-X-C-H-X_3_-H) (Hu et al. 2008), the D-TOX ZF “finger” in the DDT class (Mukherjee et al. 2009), and the SAWADEE domain (Mukherjee et al. 2009), which has been shown to be a novel chromatin-binding module that probes the methylation state of the histone H3 tail (Law et al. 2013). Further, the PHD finger is a well-characterized zinc finger resembling a RING domain and also plays a role in binding to methylated histones (Pena et al. 2006).

#### EAR and WUS repressor motifs

A number of plant homeobox genes also function as transcriptional repressors. A small repressor motif with conserved leucine residues (core consensus sequence: LxLxL), named ERF-associated amphiphilic repression (EAR), was first identified in ERF transcription factors (ethylene-responsive element binding factors) (Ohta et al. 2001). Putative EAR motives were subsequently also described in the N-terminus of HD-ZIP II HD proteins (Ciarbelli et al. 2008), in the C-terminus of several WOX HD proteins (Ikeda et al. 2009; van der Graaff et al. 2009), as well as in the N-terminus and C-terminus of BEL class proteins (therein named ZIBEL motif) (Mukherjee et al. 2009) (Fig. 3). More comprehensive searches discovered similar motives in many plant transcription factors (Causier et al. 2012; Kagale et al. 2010; Kagale and Rozwadowski 2011). Functional studies have implicated the EAR motif in repression in WOX HD proteins (Ikeda et al. 2009). WOX homeobox genes also encode a WUS box (consensus sequence TLxLFP) that has been demonstrated to be involved in repression (Ikeda et al. 2009; Lin et al. 2013). The EAR motif interacts with TOPLESS (TPL) and TPL-related proteins, which are one family of plant homologs of the animal Gro/TLE family of proteins (Causier et al. 2012; Liu and Karmarkar 2008). Thus, the interaction of WD repeat proteins of the Gro/TUP1/TPL group with transcription factors, including HD proteins, is an ancient feature of the eukaryotic transcription machinery.

### Genomic clusters of homeobox genes

A number of homeobox genes are organized into clusters and the various genome sequencing projects have uncovered more clusters since 1994. The best known clusters are the four paralogous mammalian Hox clusters with 39 Hox genes, which correspond to the *Drosophila* Antennapedia complex and the Bithorax complex (Bürglin 1994b; Bürglin 2011; Deutsch 2010; Duboule 2007; Gehring et al. 1994; Lonfat and Duboule 2015; Lonfat et al. 2014; Pick 2015; Rezsohazy et al. 2015). The Hox cluster is well conserved in tetrapods; however, as more Hox clusters have been isolated from different animals, more variation has been noted (Ikuta 2011). In teleost fish, due to the extra round of genome duplication, the Hox cluster organization can be quite capricious, with many losses (Kuraku and Meyer 2009; Martin and Holland 2014). In the genus *Drosophila*, rearrangements have occurred in the Hox cluster and it has split into two subclusters several times independently (Negre and Ruiz 2007), while in sea urchins and tunicates the organization is very disordered or split multiple times (Deutsch 2010; Duboule 2007); also, in nematodes, the cluster has substantially degenerated (Aboobaker and Blaxter 2003b). In butterflies and moths, extra Hox genes have been inserted in the cluster via duplication (Ferguson et al. 2014). *Cnidaria* have only few Hox genes (Ryan et al. 2007), and it appears that the full expansion of the Hox cluster occurred only in bilaterians (Deutsch 2010).

The Hox cluster is by no means the only homeobox gene cluster. Tandem duplication is one of the most common mechanisms in eukaryotes to increase gene diversity (Fan et al. 2008). A smaller cluster with three genes, called the ParaHox cluster, was originally found in amphioxus (*Gsx*, *Xlox* [mammalian *Pdx*], *Cdx*) (Brooke et al. 1998; Bürglin 2011). In *Drosophila*, it is disrupted, and only two genes (*ind* [*Gsx*] and *cad*) are present (Fig. 2). The ParaHox and Hox genes probably have arisen via duplication from each other. Members are present in *Placozoa* and *Cnidaria*, with the beginnings of the Hox and ParaHox clusters emerging in *Cnidaria* (Chourrout et al. 2006; Garstang and Ferrier 2013; Holland 2013; Hui et al. 2008; Srivastava et al. 2008). Together with a few additional Hox-related genes, ParaHox genes are grouped into the HOXL genes (Fig. 2).

The NK cluster (aka tinman complex) was initially discovered in *Drosophila* (Jagla et al. 2001). As more sequence information became available, further genes could be incorporated into the cluster (Bürglin 2005; Bürglin 2011; Cande et al. 2009). Beginnings of an NK cluster with several NK genes were found in the sponge *Amphimedon queenslandica*, with no evidence of HOXL genes (Larroux et al. 2007). More recently, it has emerged that at least one ParaHox/Hox-like gene already existed in sponges and that some Hox genes may have been lost in some sponges (Fortunato et al. 2014; Mendivil Ramos et al. 2012). The NK cluster homeobox genes and a number of disperse NK homeobox genes are grouped into the NKL subclass based on their HD (Fig. 2). However, since HOXL genes are likely to be derived NKL genes that have arisen later in evolution, the NKL subclass is paraphyletic with respect to the HOXL subclass genes, and therefore some classifications have abandoned this distinction (Holland 2013).

A number of other clusters exist, not counting tandem duplicated genes. The PRD-LIKE homeobox genes are usually dispersed in the genome. However, a small cluster with three genes exists, named HRO (*homeobrain*, *rx* and *orthopedia*) that is already found in *Placozoa* and *Cnidaria* (Mazza et al. 2010). This also demonstrates the ancientness of the PRD-LIKE homeobox genes. Also, in *Placozoa*, a cluster of two LIM homeobox and two LIM-only genes was found (Srivastava et al. 2010a).

Newly evolving clusters have also been discovered. In mice, the PRD-LIKE *Rhox* genes have expanded into a large cluster with 33 genes, while in humans, only three genes are present in the equivalent chromosomal location (Maclean et al. 2005; MacLean and Wilkinson 2010). Another family that has expanded in mice is the *Obox* gene family (Rajkovic et al. 2002). The PRD-LIKE *Dux* genes have also been subject to rapid evolution and duplication in mammals and primates (Leidenroth et al. 2012; Leidenroth and Hewitt 2010).

## HD structure and function

### Structure of the HD

The basic structure of the HD as a globular domain with three alpha helices had already been determined by 1994 (Bürglin 1994b; Gehring et al. 1994). Since then, numerous additional structures of HDs have been determined, often in complexes with DNA, or with additional flanking domains, or with cofactors (see Sup. Table S1). A key residue for sequence-specific DNA binding, position 50, was already defined by then, and this residue also allows one to distinguish some HD classes (Table 1). The importance of water molecules in the interface of the HD and the DNA was already noted (Bürglin 1994b; Gehring et al. 1994). More refined X-ray crystallography and modeling gave further insights into how these water molecules contribute to DNA contacts (e.g., Billeter et al. 1996; Li et al. 1995). While most of the specificity of the HD-DNA interaction resides in the major groove contacts, the minor groove contacts of the N-terminal arm contribute to the strength of the binding as well. Considerable variation can be found in the sequence of the N-terminal arm between different HD families, yet often basic residues are present in these positions. Arginine (R) is particularly favored at position 5 of the HD (Table 1, Sup. Fig. S1). Arginine residues are preferentially found in narrow minor grooves, which tend to be A-tracts (Rohs et al. 2009).

The HD contains a helix-turn-helix motif, and thus similarities to bacterial DNA-binding proteins exist (Laughon and Scott 1984). For example, the similarity to the Hin recombinase family was pointed out early on (Affolter et al. 1991). Structural analysis of the Hin recombinase shows that the DNA-binding domain is indeed composed of three alpha helices in a similar arrangement as the HD (Feng et al. 1994).

### DNA binding of the HD

The basic DNA-binding properties of the HD proteins were also known in 1994 (Gehring et al. 1994). Since then, many binding sites where determined with Selex and other methods, more recently using high-throughput approaches. This resulted in databases where binding site preferences for individual transcription factors, or their DNA-binding domain, can be looked up (Affolter et al. 2008; Berger et al. 2008; Jolma et al. 2013; Noyes et al. 2008), for example, in Jaspar (http://jaspar.genereg.net) (Mathelier et al. 2014). Mutational analysis of the En HD explored the potential binding space of the HD further; novel En HD mutant variants were uncovered that displayed substantially altered binding preferences (Chu et al. 2012).

Although this information will certainly be of great value to better understand gene regulation by HD proteins, recent evidence suggesting that clusters of low affinity sites also play important roles in gene regulation by HD proteins (Crocker et al. 2015) suggests that in silico searches using high-affinity sequences might miss many functionally relevant sites (see also below).

It is clear that the limited sequence specificity of the HD itself, comprising TA-rich sequences of hardly more than four base pairs, is not sufficient to explain how genes can be activated in a selective manner in vivo (Mann et al. 2009). Several mechanisms are exploited by HD proteins to increase DNA-binding specificity, involving either flanking domains or cofactors. In a number of cases (Fig. 3), additional domains provide extra DNA-binding capacity (e.g., PAIRED, POU, PROS, ZF, CUT). In a few cases, multiple HDs occur in a given protein (e.g., ZF class, DVE family) (Sup. Fig. S3), the most extreme case to date is found in *C. elegans*, with CEH-100 having 12 HDs (Hench et al. 2015).

In the case of cofactors, the HD or flanking regions provide protein-protein interaction interfaces that allow either other DNA-binding cofactors to bind together with the HD transcription factor (e.g., the HEX motif), allow oligomerization (e.g., COMPASS domain, or leucine zipper in HD-ZIP genes), or provide other types of protein interactions with components of the transcription machinery. Further, DNA shape plays a role in adding specificity.

Below, we discuss a few examples of the mechanisms by which HD proteins can interact with partners to exert their task as transcriptional regulators.

## DNA binding of HD proteins

### TALE-Hox interaction and DNA binding

#### MATα2-MATa1 interaction

**Fig. 5:**
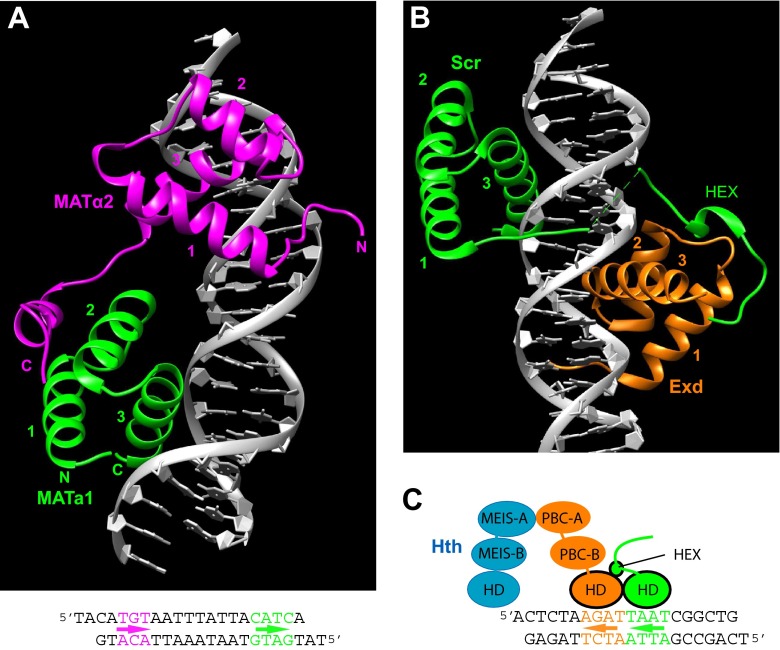
3D structures of TALE-HD/HD/DNA ternary complexes. In this and subsequent figures, UCSF Chimera was used to model the structures (Pettersen et al. 2004). The three alpha helices are numbered. **a** 3D crystal structure of the HDs of yeast MATα2 (*magenta*) and MATa1 (*green*) in a complex with DNA (PDB ID: 1YRN) (Li et al. 1995). *N* and *C* indicate N-terminal and C-terminal ends of the fragments, respectively. The DNA used as binding site is shown underneath and is color coded to show the binding regions of the two HDs. *Arrows* indicate the direction of the third alpha helix. **b** Crystal structure of the *Drosophila* Scr (*green*) and Exd (*orange*) HDs co-crystalized on the *fkh250* binding site (PDB ID: 2R5Z) (Joshi et al. 2007). The N-terminal arm of the HD and the linker to the HEX motif make multiple contacts in the minor groove. **c** *fkh250* sequence used in the Scr/Exd/DNA crystal complex (**b**), and schematic view of a hypothetical complex of the full-length proteins together with *Drosophila* Hth (Homothorax, ortholog of mammalian MEIS proteins). The two HD fragments used in the crystal are in *black outlines*, and *arrows* indicate the direction of the third alpha helix

The yeast mating type locus contains two homeobox genes, the TALE homeobox gene MATα2 and the typical homeobox gene MATa1. MATα2 can regulate different subsets of genes by forming heterodimers with either MCM1 or with MATa1. MATα2 and MATa1 individually lack strong DNA-binding affinity, but together, they bind strongly to their binding sites (Li et al. 1995). In the complexes the two HDs bind in tandem to their binding site, and MATα2 contacts the MATa1 HD via its C-terminal tail downstream of the HD (Fig. 5a). This structural complex also illustrates well, how water molecules are positioned in the interface between helix 3 and the DNA (Sup. Fig. S4).

#### PBC-Hox interaction

Hox proteins usually do not bind alone to enhancers or promoter regions. Proteins encoded by two TALE classes, PBC (Exd in *Drosophila*), and MEIS (Hth in *Drosophila*) have been shown to be important cofactors for Hox function. These TALE proteins are usually expressed rather broadly, so their segment/tissue specificity resides mostly in the Hox cofactors (Mann and Affolter 1998; Mann et al. 2009; Rezsohazy et al. 2015). This interaction is evolutionarily ancient and is also found in the sea anemone *Nematostella* (Ferrier 2014; Hudry et al. 2014; Merabet and Galliot 2015). Notwithstanding, PBC/MEIS proteins function also independently of Hox proteins (Laurent et al. 2008; Schulte and Frank 2014).

Crystal structures of several PBC and Hox HDs when bound to DNA have been determined, such as, for example, Exd-Scr, shown in Fig. 5b (Joshi et al. 2007; Mann et al. 2009). In all these structures, it was found that the HEX motif upstream of the Hox HD interacts with the PBC HD. In Abd-B proteins, the conserved tryptophane (W) plays this role in interaction. It is also interesting to note that the EH2 conserved region in En proteins, with its rudimentary similarity to the HEX motif, confers interaction with Pbx1 (Peltenburg and Murre 1996). Interaction between PBC and Hox proteins is, however, not confined to the HEX motif; in fact, this particular interaction may even be dispensable in some contexts. For example, regions at the C-terminus of the Ubx HD (named UbdA) can provide additional interaction interfaces (Foos et al. 2015).

MATα2/MATa1 and PBC/Hox interactions may have a common ancient evolutionary origin (Bürglin 1998). However, the arrangement of the two factors when bound to DNA differs between yeast (MATα2-MATa1, Fig. 5a) and metazoa (Exd-Scr, Fig. 5b), in that the two protein types exchanged positions (TALE-typical → typical-TALE, note arrows in Fig. 5c). Perhaps, this is a consequence of a secondary loss of an upstream MEIS or PBC domain in MATα2, necessitating changes in the dimer interactions. However, MEIS proteins may also bind on the other side of the Hox protein as in the class 3 interactions shown in Merabet and Lohmann (2015) (Fig. 5c).

The current structural studies are limited by the fact that neither is the highly conserved upstream PBC domain included, nor are structures of complexes available that include the MEIS protein. MEIS/Hth interacts with PBC through the N-terminal subdomain (PBC-A) (Fig. 5c) and MEIS/Hth can also interact with the Hox proteins (Amin et al. 2015; Mann and Affolter 1998; Mann et al. 2009; Merabet and Hudry 2013; Merabet and Lohmann 2015). In such multimeric complexes, DNA specificity would of course be further increased.

#### DNA shape plays a role in DNA-binding specificity

Even though TALE cofactors increase the DNA specificity of the Hox proteins, the HDs of Hox proteins themselves are still very similar in sequence and bind to similar sequences. Thus, the conundrum how individual Hox proteins can exert their highly specific functions in different tissues in vivo is still not sufficiently resolved. Recent insights into how PBC/Hox complexes bind DNA may aid in resolving this puzzle: DNA shape, i.e., structural features such as minor groove width, roll, and twist, also play an important role in specificity (Abe et al. 2015; Dror et al. 2014; Rohs et al. 2009; Slattery et al. 2011; Yang et al. 2014). Exd/Hox dimers display different binding specificities (Rohs et al. 2009). DNA shape contributes to these differences, and DNA shape predictions revealed that anterior and posterior Hox proteins prefer sequences with distinct minor groove topographies (width minima) (Dror et al. 2014; Yang et al. 2014). A key residue for minor groove contacts is arginine at position 5 of the HD. In the case of Exd/Scr binding to the fkh250 site, two additional residues, arginine 3 and histidine -12, also insert into the minor groove (Joshi et al. 2007) (Fig. 5b), and they are important for the binding preferences of Scr, since they select DNA sequences with a narrow minor groove at the Hox half-site (Abe et al. 2015). Future in silico prediction of DNA-binding specificities will certainly benefit by taking such DNA structural features into account (Abe et al. 2015).

#### Additional protein-protein interactions provide specificity

Protein-protein interaction is not restricted to PBC/Hox interactions via the HEX motif. Experiments with *Drosophila* Scr have shown that in salivary glands, Scr but not Antp can form homodimers (Papadopoulos et al. 2012). Glutamine at position 19 in helix 1, which is found in many Hox proteins (Sup. Fig. S1), is critically important for this dimerization. However, Antp fails to dimerize because of short regions N- and C-terminal of the HD (Papadopoulos et al. 2012). Such short linear motifs (SLiMs), an example of which is the HEX motif, are becoming the focus of further studies, since they can contribute to differential, specific protein-protein interaction in different tissues (Merabet and Galliot 2015; Merabet et al. 2009; Sivanantharajah and Percival-Smith 2015). In an in vivo screen, many novel protein-protein interactions that occur in different cellular contexts were identified (Baëza et al. 2015). These studies showed that the HEX and the UbdA motives play key roles in providing specificity and that mutations in these motives can shift the interaction profile. A further mode of selective protein-protein interaction has been demonstrated for the mediator complex. It can bind to the HD of some Hox proteins, but not to other HDs, such as those in PBC proteins (Boube et al. 2014).

#### Low-affinity binding sites

Protein-protein interactions as well as DNA shape can address some of the conundrum of how TALE-Hox proteins discriminate between different promoters/enhancers. Somewhat contradictory is the observation of low-affinity binding sites with reduced specificity (Gehring et al. 1994). In recent experiments, Crocker et al. showed that Ubx together with the Exd and Hth cofactors binds to low-affinity sites in the promoter of the *shavenbaby* (*svb*) gene (Crocker et al. 2015; Merabet and Lohmann 2015). Multiple low-affinity binding sites were required to achieve robust expression of this promoter. Mutation of these binding sites to high-affinity consensus sites decreased tissue specificity, allowing other Hox factors to bind and broaden the expression domain. Thus, while low-affinity sites might be able to better discriminate between different TALE-Hox complexes, multiple sites are necessary to compensate for such low-affinity sites.

### PAIRED (PRD) class

The PRD class of homeobox genes was of prime interest to Walter, ever since the discovery that the *Drosophila* gene *eyeless* is homologous to vertebrate PAX-6 genes, and that these genes are involved in eye development (*Small eye* mutations in mouse, *Aniridia* mutations in human) (Quiring et al. 1994). This triggered a fruitful line of research into the origin and evolution of eyes in his laboratory resulting in highlights such as the spectacular finding that *Drosophila eyeless* and mammalian PAX-6 can induce ectopic eyes in tissues such as legs and wings in *Drosophila* (Gehring 2005; Gehring 2012; Gehring 2014; Halder et al. 1995; Hayakawa et al. 2015).

#### PRD class classification

**Fig. 6:**
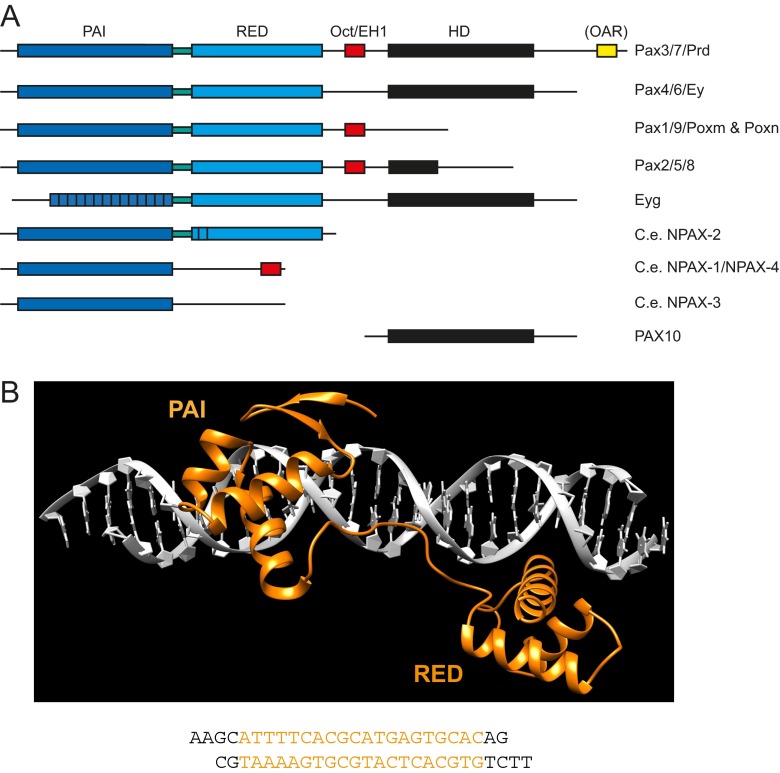

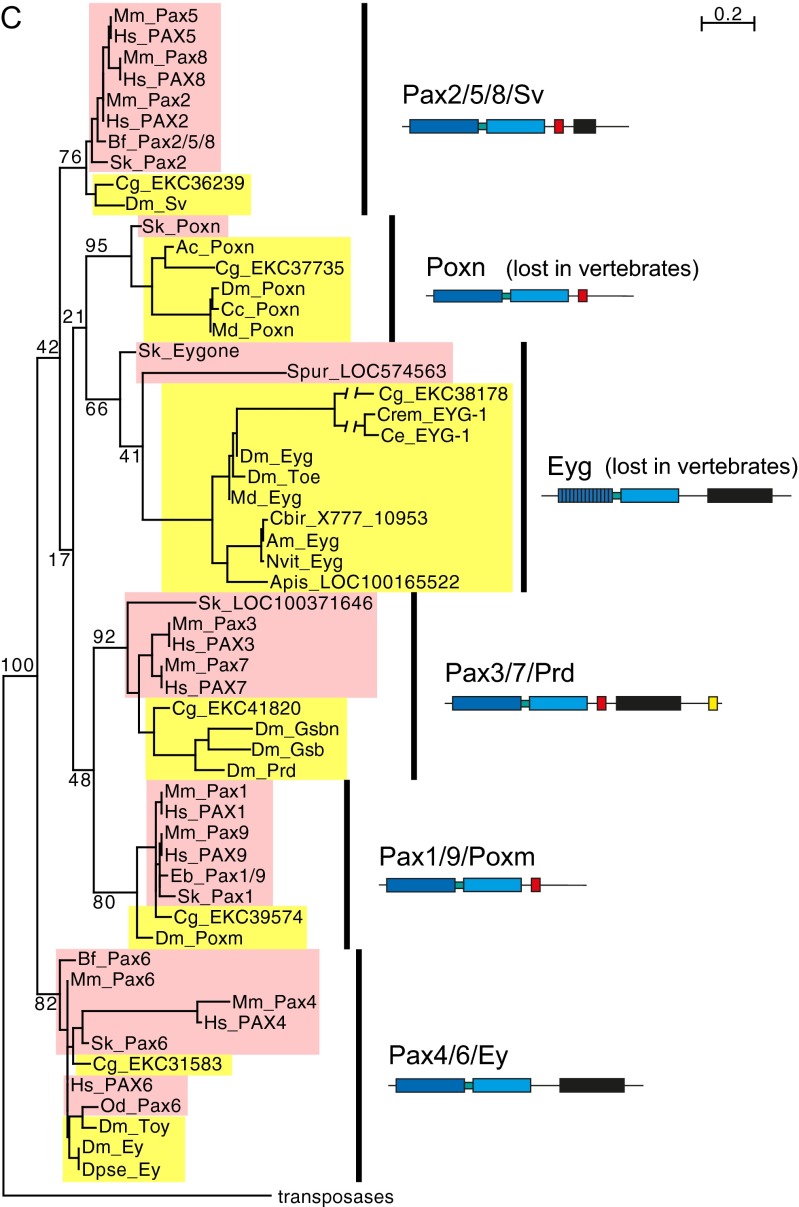
PRD class of homeobox genes. **a** Schematic view of the variable domain and motif organization found in different PRD families or proteins. The PAIRED domain is composed of two subdomains, PAI and RED, separated by a linker. *Brackets* indicate motifs not present in all genes of a family. **b** Structure of the PAIRED domain of human PAX6 bound to DNA (PDB ID: 6PAX) (Xu et al. 1999). PAI and RED domains are indicated. The bound sequence is shown underneath. The beta-strands and alpha helices are marked in the sequence alignment in Sup. Fig. S5. **c** Rooted phylogenetic tree of bilaterian PRD class proteins, based on the PAIRED domain. The PAIRED domain similarity region of four bilaterian transposases (from acorn worm and oyster) was used as outgroup. Values of 100 bootstraps values are shown at selected clades. On the *right side*, the PRD class families are indicated, together with their typical structural organization. Deuterostome branches are highlighted in pink, protostome branches in *yellow*. Branch lengths in the Eyg family were reduced where indicated (Ce, Crem, CG). Note that the branching of the six families should not be taken as evidence for how they evolved from an ancestral precursor. *Drosophila* proteins: Sv: Shaven; Poxn: Pox-neuro; Poxm: Pox-meso. The phylogenetic tree was created using PhyML as implemented in SeaView (Gouy et al. 2010). About 100 residues containing the C-terminal region of PAI and the complete RED subdomain from the multiple sequence alignment in Sup. Fig. S5 were used for tree generation. For species codes, see Sup. Fig. S5

PRD class homeobox genes encode a PAIRED domain and a HD with most often a serine residue at position 50 of the HD. In addition, PRD proteins may contain an EH1/Octapeptide motif and an OAR motif (Fig. 6a). In many species, PRD genes are called Pax genes, and in several instances, they have lost their homeobox secondarily (see below).

The PAIRED domain was originally discovered by Daniel Bopp and colleagues in the laboratory of Markus Noll at the Biozentrum (Bopp et al. 1986; Noll 1993). Structural studies show that the PAIRED domain, which is about 128 amino acids long, is composed of two subdomains (Xu et al. 1995), which were named PAI and RED (Jun and Desplan 1996). In sponges and *Placozoa*, only a single Pax gene exists, while in *Cnidaria*, four types of genes are found (PaxA, PaxB, PaxC, PaxD) (Hill et al. 2010; Suga et al. 2010). In bilaterians, five distinct PRD families have been described (Miller et al. 2000; Underhill 2012).

Only recently has it become apparent that there are actually six families in bilaterians (Fig. 6c), since the Eyegone (Eyg) family is also found in hemichordates and sea urchins (Friedrich and Caravas 2011). Our own phylogenetic analysis confirmed this finding, both when the PAIRED domain was used (Fig. 6c), and when the HD was used (Fig. 2). Two families, Eyg and Poxn, have been lost in vertebrates.

The PAIRED domain has significant sequence similarity to Tc1-like transposases (Ivics et al. 1996), suggesting that it was derived from a transposase (Breitling and Gerber 2000). A PRD domain-like protein has also been found in the protozoan *Giardia lamblia* (Wang et al. 2010), though its relationship to the transposases and the PAIRED domain has not been elucidated yet. The HD sequences of PRD class proteins are most similar to those of PRD-LIKE HDs (Fig. 2), suggesting that a Paired box gene merged with a PRD-LIKE homebox gene in early metazoan evolution prior to the emergence of sponges and *Placozoa* (Galliot et al. 1999; Underhill 2012). Some of the PRD class homeobox genes encode two additional motifs, i.e., the EH1 and the OAR motif. The OAR motif is present only in Pax7 proteins (but not Pax3) and is also found in PAX3/7 homologs of oyster (Sup. Fig. S5), demonstrating that this motif has been conserved across the bilaterian divide. Given that these motifs also exist in several PRD-LIKE families, the most parsimonious explanation is that the original PRD class gene that captured a PRD domain also had an EH1 and an OAR motif (Underhill 2012; Vorobyov and Horst 2006).

#### The PAIRED domain structure

Several 3D structures of the PAIRED domain have been determined (Sup. Table S1), and the DNA-binding specificity of several has been investigated (Mayran et al. 2015). The structure consists of two subdomains (Fig. 6b). The N-terminal PAI subdomain is characterized by a short beta motif and a domain with three alpha helices that fold in a helix-turn-helix fashion similar to the HD. The C-terminal RED subdomain also contains three alpha helices that fold in a HD-like fashion (Xu et al. 1999). The two subdomains are joined by a linker region of eight amino acids. The first structure of the PAIRED domain of the *Drosophila* Paired protein bound to DNA revealed that the main DNA contacts were made by the PAI subdomain (Xu et al. 1995). The subsequent X-ray structure of Pax-6 showed that the RED domain also can contact the DNA, and that the linker region of Pax-6 makes extensive contacts with the minor groove of the DNA (Xu et al. 1999).

#### Molecular tinkering in the PRD class

The PRD class genes represent an interesting case of molecular tinkering (Jacob 1977), an idea which Walter was particularly fond of. While the original PRD gene most certainly encoded a complete PAIRED domain (PAI and RED), an EH1 motif, a HD, and an OAR motif, we find that through loss of motifs, a wide variation of combinations has been created (see Fig. 6a, Sup. Fig. S5). Only a subset of the Pax3/7/Prd family (Pax7 and some invertebrate genes) has retained all motifs, while OAR was lost from most of the other genes (Vorobyov and Horst 2006). The Pax4/6/Ey family lost the EH1 motif, and Pax1/9/Poxm as well as Poxn lost the HD. Interestingly, the Pax2/5/8/Sv family lost only the last half of the HD in vertebrates, although in *Drosophila* Sv this HD remainder completely diverged. The Eyg family evolved the PAI subdomain rapidly, losing at least the N-terminal beta strands (Friedrich and Caravas 2011), and in the most extreme case in nematodes, the whole PAI subdomain was lost (Hobert and Ruvkun 1999). Conversely, nematodes also code for proteins that only retained the PAI domain (Hobert and Ruvkun 1999), and two of these also contain the EH1 motif (NPAX-1/NPAX-4, Fig. 6a, Sup. Fig. S5). Finally, in vertebrates, a Pax10 protein (Pax3/6/Prd family) exists that lost the PAIRED domain; this protein itself was lost in mammals (Feiner et al. 2014; Ravi et al. 2013). The Pax3/6/Prd family lost the EH1 motif, although another conserved motif is present in a similar location (named PAX6 in Sup. Fig. S5). It has been suggested that this motif is Octapeptide-like (Keller et al. 2010). However, this motif does not have the characteristic conserved pattern of hydrophobic resides.

The variation of domains and motifs is also replicated to some extent within individual genes through alternative splicing, which can alter DNA-binding specificity (Underhill 2012). For example, the *C. elegans* Pax-6 gene *vab-3* has an alternative splice form (*mab-18*) that lacks the Paired box (Zhang and Emmons 1995). Another example is the alternative splicing of *Pax-3* in olive flounder, which can produce transcripts encoding a disrupted PAIRED domain, and/or lacking a HD (Jiao et al. 2015). Experimentally, the functional separation of the PAIRED domain and the HD has also been demonstrated: a construct of Ey lacking the HD is able to rescue the *ey2* mutant phenotype (Punzo et al. 2001).

### SIX/SO (aka SINE) class

The SIX/SO class of HD proteins is characterized by a 120 amino acids long SIX/SO domain upstream of the HD (Fig. 3). The HD itself is also noteworthy, since basic residues in the N-terminal region of the HD are absent (Sup. Fig. S1, Table 1), which suggests that the N-terminal arm may not interact with the minor groove of the DNA.

**Fig. 7:**
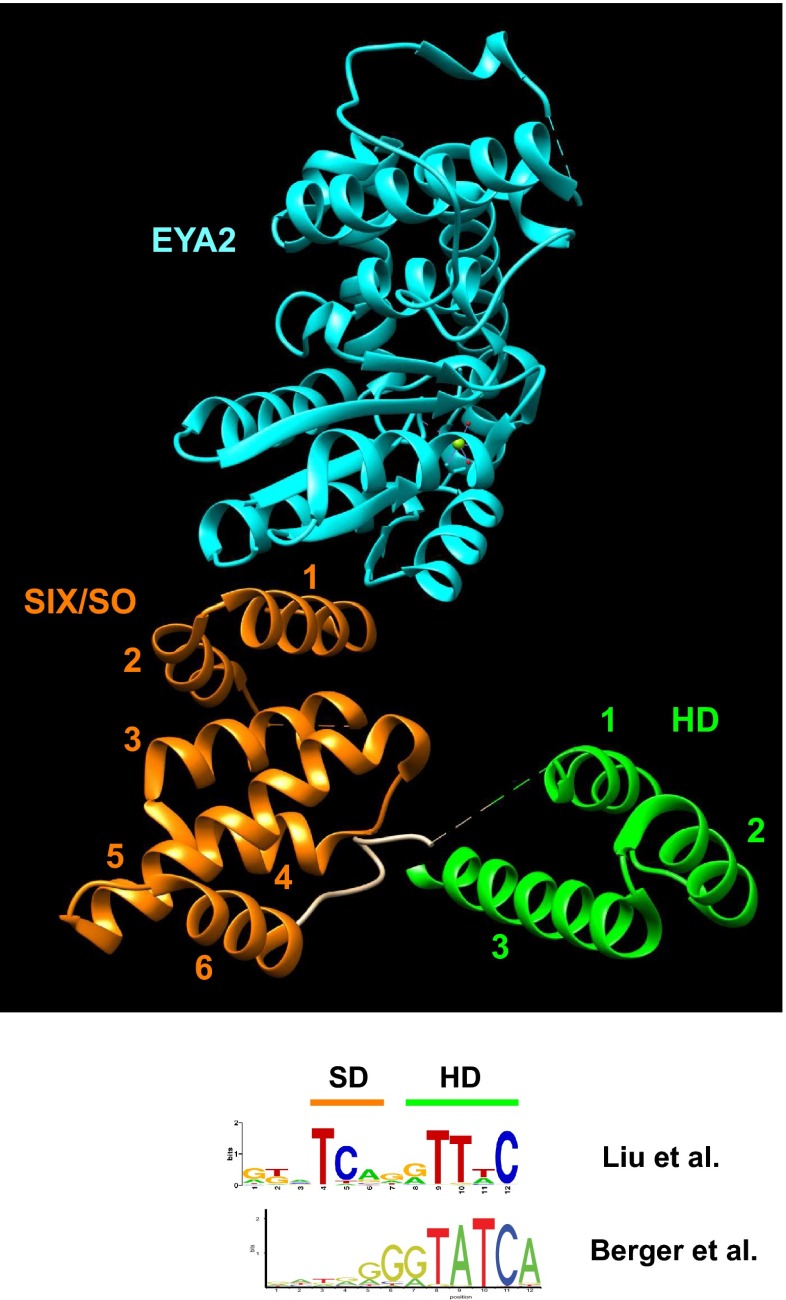
3D structure of human SIX1 bound to the human Eyes Absent protein EYA2 (PDB ID: 4EGC) (Patrick et al. 2013). Note that the MBP fusion protein, which is part of the crystal, is hidden in this view. The HD is in *green*, the SIX/SO domain is in *orange*, and EYA2 is in *cyan*. Helix 6 of the SIX/SO domain and helix 3 of the HD are arranged such that they could fit in the major groove of the DNA. Underneath, the DNA logo of the binding site determined for mouse Six1 is shown, visualized using Weblogo (Crooks et al. 2004) with the sequences from Sup. Table 2 from (Liu et al. 2012). The binding site logo determined in the high-throughput study by Berger et al. (2008) using the mouse Six1 HD only (Berger et al. 2008) is shown at the bottom, retrieved and visualized using Jaspar (Mathelier et al. 2014)

Two SIX/SO class genes, *Optix* (Six3 family) and *sine oculis* (*so*, Six1 family), play a role in eye development like several of the PRD homeobox genes. *Drosophila* Eyes absent (Eya), a special protein tyrosine phosphatase, has been shown to be a cofactor of SIX/SO proteins. Molecular and structural analysis revealed that human SIX1 interacts directly with human EYA2 (Patrick et al. 2013). The SIX/SO domain is a globular domain comprised of six alpha helices that has no obvious similarity to other structures (e.g., helix-turn-helix motifs, etc., Fig. 7). Modeling and mutational analyses suggest that the sixth helix binds in the major groove of the DNA, and together with the third helix of the HD, the two domains provide specific DNA binding. EYA2 does not bind DNA, instead it interacts with helix 1 of the SIX/SO domain and provides thus the co-activator role for SIX1 (Patrick et al. 2013). DNA-binding studies suggest that the SIX/SO domain modifies the DNA-binding properties of the HD. Berger et al. (2008) produced a DNA-binding profile for the SIX1 HD (core TATC, Fig. 7) in their high-throughput study (Berger et al. 2008), which differed from the profile identified when full-length SIX1 was used (TT[t/a]C) (Liu et al. 2012) (Fig. 7). In addition, an additional conserved pair of residues, TC, was revealed, which we suggest is bound by helix 6 of the SIX/SO domain. The latter motif matches the binding site MEF3 (consensus: TCAGGTTTC) (Patrick et al. 2013). Overall, this illustrates how through the action of a tethered flanking DNA-binding domain, the specificity of the HD can be altered.

### POU class

The POU class of transcription factors is characterized by a conserved POU-specific domain of about 70 resides that is located upstream of a HD that usually contains a serine residue at position 50 of the HD (Herr et al. 1988) (Table 1). The POU domain has so far not been found independently of the HD, unlike the PAIRED domain discussed above. X-ray structures have shown that the POU domain is composed of a compact, globular DNA-binding domain with four alpha helices that have a similar fold to bacteriophage repressor molecules (Assa-Munt et al. 1993). Helices 2 and 3 form a helix-turn-helix motif and base-specific contacts are made with helix 3 in the major groove of the DNA.

**Fig. 8:**
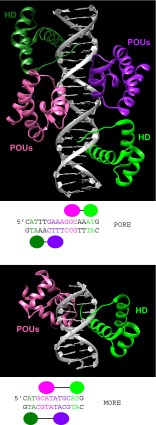
3D structure of the POU-specific domain of human OCT1 bound to DNA in two different configurations (Reményi et al. 2001). The HD is in *green*, and the POU-specific domain is in *magenta. Underneath each panel* are the respective binding sites used in the X-ray studies as well as schematic views of OCT1 DNA-binding domains. **a** OCT1 dimer binding to the PORE DNA sequence. The two PAX6 monomers are distinguished by different color intensity (PDB ID: 1HF0). **b** OCT1 bound to the MORE DNA sequence (PDB ID: 1E30). Note that only half of the dimer is shown, due to the complete symmetry in conjunction with the palindromic binding site.

POU proteins can bind as homodimers or heterodimers to DNA. The human POU protein OCT1 (POU2F1) exemplifies another way of increasing as well as modulating DNA-binding specificity. OCT1 binds as a dimer, and strikingly, can bind in two different conformations (Reményi et al. 2001). In one conformation, it binds to a DNA sequence termed PORE (Fig. 8a), which is not palindromic. In this configuration, the binding site is longer (15 bp), and the two molecules sit further apart so that their respective binding sites are adjacent to each other. In the second configuration, the binding to the palindromic MORE DNA sequence is symmetric and the site is shorter (12 bp) (Fig. 8b). Here, each dimer is oriented more longitudinally along the DNA so that the two POU domains bind the DNA in a nested fashion.

The protein-protein contact interfaces between these two configurations are very different; in the case of MORE, the C-terminus of the HD contacts the POU domain at the N-terminus of helix 1 and the loop between helices 3 and 4, while in the PORE configuration, the N-terminus of the HD contacts the POU domain in the helix 1 to helix 2 region (Reményi et al. 2001). Thus, two different configurations of how the protein can bind to DNA yield different sequence specificities. These provide also different interfaces for other proteins to interact with OCT1 so that cofactors, e.g., OBF1 (POU2AF1), only recognize one conformation (Reményi et al. 2004).

## Conclusion

HD proteins predominantly function as transcription factors that activate or repress gene expression. We have seen that a HD by itself is not sufficiently specific to bind to targets in gene promoters. On the one hand, additional flanking domains, or cofactors are used to add extra specificity. These extra protein domains cannot only add specificity, but they can also alter the specificity of the HD itself. How the necessary specificity is achieved in vivo is still poorly understood. DNA shape appears to play an important role in addition to the base pair sequence, and contributes to specificity. On the other hand, even low-affinity sites are used. This is compensated to some degree through the use of multiple copies of such binding sites to achieve specificity. Other interacting cofactors may also contribute towards stabilizing low-affinity sites. Overall, the most common theme is that the combinatorial interaction of multiple factors is required in the regulatory region of a specific gene for proper regulation. Most HD proteins can interact with multiple partners, either other HD proteins (both homo- or hetero-interactions are possible), or other types of transcription factors. Many of these interactions can be mediated by SLiMs. Some of the motifs are required for coupling to the transcription machinery, e.g., EH1, which can mediate a repression state.

The discovery of the homeobox genes was seminal for our improved understanding of developmental and evolutionary biology. Initially, as exemplified by the homeotic genes, it demonstrated that sequence-related proteins can play similar yet different functions, i.e., that duplicated and subsequently divergent paralogous genes allowed specialization in different body segments. Likely, the expansion of the homeobox genes contributed to the Cambrian explosion (Holland 2015).

The realization that homeobox genes are well conserved from flies to vertebrates provided a fundamental technological revolution: in a flurry of activity many different types of developmental control genes were isolated based on sequence similarity. A new area of reverse genetics was born that allowed breakthroughs in mammalian developmental biology. Furthermore, it demonstrated for the first time that the fundamental molecular mechanisms underlying metazoan development are evolutionarily conserved. Another consequence of these findings, though it may seem obvious nowadays, was the realization that transcription factors play a key role in decoding the genetic blueprint and converting it through a cascade of events into cell fate decisions and cell differentiation that ultimately gives rise to a complex multicellular organism.

Further insights into development and evolution of organisms stem from the knowledge gained over the last decades about key regulators of developmental processes, whether they are transcription factors, signaling molecules, or regulatory RNAs. Homeobox genes represent only a subset of all these regulators, yet their analysis has provided many important insights into the evolutionary events that have taken place over hundreds of million years.

For example, how can major changes in body morphology evolve? We now know that a mutation in a HD transcription factor can lead to drastic altered body shapes, since a whole cascade of downstream target genes is affected (e.g., Ronshaugen et al. 2002). Thus, evolutionary events need not only occur in small, gradual steps, but larger jumps are also possible, although they may not be as frequent.

Another example is gene loss. It is perhaps self-evident that, as the multicellular complexity of an organism grows, the number of regulatory factors has to increase. This is well exemplified by the increase in the number of homeobox genes when going from single-celled eukaryotes to multicellular plants or animals. Conversely, one might perhaps expect that losing key developmental regulators such as homeobox genes, once acquired, would be a taboo. Yet, we observe again and again that homeobox genes were lost in evolution. For example, the Hox cluster in *C. elegans* is very degenerate and several genes were lost (Aboobaker and Blaxter 2003a; Aboobaker and Blaxter 2003b). Similarly, the PRD class shows that two of its families, one comprising the apparently “important” gene *eyegone*, were lost early in the chordate lineage (Fig. 6). Most impressive is the loss of 34 homeobox families in parasitic tapeworms (Tsai et al. 2013).

A last example is evolutionary innovation. The *Drosophila* homeobox gene *bcd* is essential for early embryogenesis, where its protein forms a gradient through the embryo (Driever and Nüsslein-Volhard 1988). Yet, *bcd* is an evolutionary novelty, existing only in Cyclorrhaphan flies, and the gene itself was derived from a Hox3 cluster gene (Stauber et al. 1999; Stauber et al. 2002). A related observation is that 14 homeobox genes in *C. elegans* lack obvious orthologs in other *Caenorhabditis* species (Hench et al. 2015). Clearly, they have emerged only recently, and seem to be subject to rapid evolutionary change.

The last 20 years were certainly very exciting for Walter. He was still scientifically active until the last moment, and alas, he will not see the results of his latest experiments. Nonetheless, he had the satisfaction of seeing many, though by no means all, of his scientific predictions and hypotheses confirmed.

## Electronic supplementary material

Below is the link to the electronic supplementary material.

Sup. Fig. S1 Multiple sequence alignment of 103 *Drosophila melanogaster* HDs, supplemented with a select few additional HDs from other species. Sequences were collected from HomeoDB (Zhong and Holland 2011b), corrected, and updated. The *Drosophila* Shaven protein was excluded, as it lacks the partial Pax2/5/8 HD of vertebrates (Sup. Fig. S5). An extra gap was introduced upstream of helix 1 that gives a better alignment at the N-terminus for the two Dve HDs. In human HNF1A twelve residues were omitted at the 'x' in the second loop. The default color code of Clustal X was used (Larkin et al. 2007); it colors conserved residues with similar properties. Species abbreviations: Dm: *Drosophila melanogaster*; Hs: human; Dr: *Danio rerio* (zebrafish); Spur: *Strongylocentrotus purpuratus* (purple sea urchin); Sk: *Saccoglossus kowalevskii* (acorn worm; hemichordate); Ce: *Caenorhabditis elegans*; Pt: *Paramecium tetraurelia* (sequence accession number: XP_001455625). (PDF 3.03 MB)

Sup. Fig. S2 Multiple sequence alignment of fungal MATα2 proteins. Default color code from SeaView (Gouy et al. 2010). Species abbreviations: Scer: *Saccharomyces cerevisiae*; Vpol: *Vanderwaltozyma polyspora*; Kafr: *Kazachstania africana*; Zsap: *Zygosaccharomyces sapae*; Tpha: *Tetrapisispora phaffii*; Ndai: *Naumovozyma dairenensis*; Knag: *Kazachstania naganishii*; Ncas: *Naumovozyma castellii*; Tbla: *Tetrapisispora blattae*; Agos: *Ashbya gossypii*; Ecym: *Eremothecium cymbalariae*; Klac: *Kluyveromyces lactis*; Cgla: *Candida glabrata*; Kdob: *Kluyveromyces dobzhanskii*; Kmar: *Kluyveromyces marxianus*; Ndel: *Nakaseomyces delphensis*; Tdel: *Torulaspora delbrueckii*; Skud: *Saccharomyces kudriavzevii*; Zrou: *Zygosaccharomyces rouxii*. (PDF 395 kb)

Sup. Fig. S3 Schematic domain organization of ZF HD proteins. Human (h), selected *Drosophila*, and amphioxus ZF class HD proteins are shown schematically using the output from the SMART domain server (Letunic et al. 2015) with some manual corrections. The *HOMEZ* gene was initially named based on two putative leucine zippers encoded in the mammalian genes (Bayarsaihan et al. 2003). However, these predicted zippers are not conserved in fish, and SMART (Letunic et al. 2015) as well as Interpro (Mitchell et al. 2015) do not identify them as zippers, leaving their functional significance in doubt. (PDF 6.92 MB)

Sup. Fig. S4 3D crystal structure of the HDs of yeast MATα2 and MATa1 bound to DNA as in Fig. 5a, with the addition of the water molecules, which are visualized as red spheres. Left panel: same perspective as in Fig. 5a. Right panel: rotated to provide a side view of the third helix of MATα2 with the water molecules in the major groove. (PDF 2.73 MB)

Sup. Fig. S5 Sequence alignment of selected PRD class proteins generated with SeaView (Gouy et al. 2010) and Clustal X (Larkin et al. 2007). Sequences were extracted from Genbank after a blastp search using a PAIRED domain as seed (Johnson et al. 2008). The different domains are marked. Manual sequence alignment for the shorter motifs was necessary; hence sequence alignments outside the indicated motifs may not be optimal. Furthermore, the usual caveats apply, i.e. ORFs derived from genome projects may contain errors due to mistakes in the initial sequence or assembly, and/or in the subsequent ORF prediction and annotation. Note that the OAR motif in the C-terminal region of Bf_Pax2/5/8, as proposed by (Vorobyov and Horst 2006), is not present. Our analysis of the C-terminal Pax2/5/8 sequences of *Branchiostoma floridae* (Putnam et al. 2008), *B. belcheri* (Huang et al. 2014), and *B. lanceolatum* (Oulion et al. 2012) did not show the proposed frame shift that would be required to place the out-of-frame OAR-like motif at the C-terminus of Pax2/5/8; the sequence similarity may be fortuitous. A PRD class sequence from oyster (Cg) does have an OAR motif matching vertebrate PAX7 proteins. As an update to the previous publication on NPAX genes (Hobert and Ruvkun 1999), we note that new transcript data of NPAX-2 reveal that it also encodes a divergent RED domain, and that NPAX-1 and NPAX-4 encode an EH1 motif, which is conserved in other nematodes (e.g., *Ancylostoma ceylanicum*). Pax-6 proteins from flies and human, but not vertebrate Pax-4 proteins, contain a conserved motif (marked PAX6) between the PAIRED domain and the HD. It has been proposed that this motif is reminiscent of the EH1 motif (Keller et al. 2010). However, we propose a different, shifted alignment that would take the key hydrophobic residues better into consideration with respect to the EH1 profile. In our case, the D.m. Eyeless sequence “YEKLRLL” would align with the EH1 consensus “YSINGIL”. In this alignment the hydrophobic position 3 would have swapped with a polar residue at position 4 (underlined above). Whether this “PAX6” motif functions indeed like EH1 and interacts with Gro would have to be experimentally tested; the shifted hydrophobic position may impair the Gro interaction. The “PAX6” motif may instead be an amphipathic helix that interacts with another type of protein. Species abbreviations: Mm: mouse; Hs: human; Dr: *Danio rerio* (zebrafish); Bf: *Branchiostoma floridae* (amphioxus); Eb: *Eptatretus burgeri* (inshore hagfish); Od: *Oikopleura dioica* (tunicate); Sk: *Saccoglossus kowalevskii* (acorn worm; hemichordate); Spur: *Strongylocentrotus purpuratus* (purple sea urchin); Ac: *Aplysia californica* (California sea hare; mollusk); Cg: *Crassostrea gigas* (Pacific oyster; mollusk); Dm: *Drosophila melanogaster*; Dpse: *Drosophila pseudoobscura pseudoobscura*; Cc: *Ceratitis capitata* (Mediterranean fruit fly); Md: *Musca domestica* (housefly); Am: *Apis mellifera* (honey bee); Nvit: *Nasonia vitripennis* (jewel wasp); Cbir: *Cerapachys biroi* (raider ant); Apis: *Acyrthosiphon pisum* (pea aphid); Ce: *Caenorhabditis elegans*; Crem: *Caenorhabditis remanei*; Acey: *Ancylostoma ceylanicum* (hookworm; nematode). (PDF 1.19 MB)

Sup. Table S1 List of 3D structures (X-ray or NMR) for HDs, HD proteins, or associated domains as Excel spreadsheet. PDB (RCSB Protein Data Bank, http://www.rcsb.org) accession numbers are given in the left column. (XLS 147 kb)

## References

[CR1] Abe N, Dror I, Yang L, Slattery M, Zhou T, Bussemaker HJ, Rohs R, Mann RS (2015) Deconvolving the recognition of DNA shape from sequence. Cell 161:307–318. doi:10.1016/j.cell.2015.02.008 10.1016/j.cell.2015.02.008 PMC4422406 25843630

[CR2] Aboobaker A, Blaxter M (2003). Hox gene evolution in nematodes: novelty conserved. Curr Opin Genet Dev.

[CR3] Aboobaker AA, Blaxter ML (2003). Hox gene loss during dynamic evolution of the nematode cluster. Curr Biol.

[CR4] Affolter M, Müller M (2014). Walter Jakob Gehring (1939-2014). Dev Cell.

[CR5] Affolter M, Percival-Smith A, Müller M, Billeter M, Qian YQ, Otting G, Wüthrich K, Gehring WJ (1991). Similarities between the homeodomain and the Hin recombinase DNA-binding domain. Cell.

[CR6] Affolter M, Slattery M, Mann RS (2008). A lexicon for homeodomain-DNA recognition. Cell.

[CR7] Affolter M, Wüthrich K (2014). Walter Jakob Gehring: a master of developmental biology. Proc Natl Acad Sci U S A.

[CR8] Allen JD, Lints T, Jenkins NA, Copeland NG, Strasser A, Harvey RP, Adams JM (1991). Novel murine homeo box gene on chromosome 1 expressed in specific hematopoietic lineages and during embryogenesis. Genes Dev.

[CR9] Alpy F, Tomasetto C (2014). START ships lipids across interorganelle space. Biochimie.

[CR10] Amin S, Donaldson IJ, Zannino DA, Hensman J, Rattray M, Losa M, Spitz F, Ladam F, Sagerstrom C, Bobola N (2015). Hoxa2 selectively enhances Meis binding to change a branchial arch ground state. Dev Cell.

[CR11] Aravind L, Iyer LM (2012). The HARE-HTH and associated domains: novel modules in the coordination of epigenetic DNA and protein modifications. Cell Cycle.

[CR12] Assa-Munt N, Mortishire-Smith RJ, Aurora R, Herr W, Wright PE (1993). The solution structure of the Oct-1 POU-specific domain reveals a striking similarity to the bacteriophage l repressor DNA-binding domain. Cell.

[CR13] Baëza M, Viala S, Heim M, Dard A, Hudry B, Duffraisse M, Rogulja-Ortmann A, Brun C, Merabet S (2015) Inhibitory activities of short linear motifs underlie Hox interactome specificity in vivo. Elife 4 doi:10.7554/eLife.06034 10.7554/eLife.06034 PMC4392834 25869471

[CR14] Bayarsaihan D, Enkhmandakh B, Makeyev A, Greally JM, Leckman JF, Ruddle FH (2003). *Homez*, a homeobox leucine zipper gene specific to the vertebrate lineage. Proc Natl Acad Sci U S A.

[CR15] Bellaoui M, Pidkowich MS, Samach A, Kushalappa K, Kohalmi SE, Modrusan Z, Crosby WL, Haughn GW (2001). The *Arabidopsis* BELL1 and KNOX TALE homeodomain proteins interact through a domain conserved between plants and animals. Plant Cell.

[CR16] Berger MF, Badis G, Gehrke AR, Talukder S, Philippakis AA, Pena-Castillo L, Alleyne TM, Mnaimneh S, Botvinnik OB, Chan ET (2008). Variation in homeodomain DNA binding revealed by high-resolution analysis of sequence preferences. Cell.

[CR17] Bertolino E, Reimund B, Wildt-Perinic D, Clerc RG (1995). A novel homeobox protein which recognizes a TGT core and functionally interferes with a retinoid-responsive motif. J Biol Chem.

[CR18] Bharathan G, Janssen BJ, Kellogg EA, Sinha N (1997). Did homeodomain proteins duplicate before the origin of angiosperms, fungi, and metazoa?. Proc Natl Acad Sci U S A.

[CR19] Billeter M, Güntert P, Luginbühl P, Wüthrich K (1996). Hydration and DNA recognition by homeodomains. Cell.

[CR20] Blumberg B (2014) Andrés Carrasco (1946-2014). Dev Biol 393:1-2 10.1016/j.ydbio.2014.07.001 25275141

[CR21] Bodmer R (1995). Heart development in *Drosophila* and its relationship to vertebrates. Trends Cardiovasc Med.

[CR22] Boehm T, Foroni L, Kaneko Y, Perutz MF, Rabbitts TH (1991). The rhombotin family of cysteine-rich LIM-domain oncogenes: distinct members are involved in T-cell translocations to human chromosomes 11p15 and 11p13. Proc Natl Acad Sci U S A.

[CR23] Bopp D, Burri M, Baumgartner S, Frigerio G, Noll M (1986). Conservation of a large protein domain in the segmentation gene *paired* and in functionally related genes of *Drosophila*. Cell.

[CR24] Boube M, Hudry B, Immarigeon C, Carrier Y, Bernat-Fabre S, Merabet S, Graba Y, Bourbon HM, Cribbs DL (2014). *Drosophila melanogaster* Hox transcription factors access the RNA polymerase II machinery through direct homeodomain binding to a conserved motif of mediator subunit Med19. PLoS Genet.

[CR25] Brandt R, Cabedo M, Xie Y, Wenkel S (2014). Homeodomain leucine-zipper proteins and their role in synchronizing growth and development with the environment. J Integr Plant Biol.

[CR26] Breitling R, Gerber JK (2000). Origin of the paired domain. Dev Genes Evol.

[CR27] Brooke NM, Garcia-Fernàndez J, Holland PWH (1998). The ParaHox gene cluster is an evolutionary sister of the Hox gene cluster. Nature.

[CR28] Bürglin TR (1994). A *Caenorhabditis elegans prospero* homologue defines a novel domain. Trends Biochem Sci.

[CR29] Bürglin TR, Duboule D (1994). A comprehensive classification of homeobox genes. Guidebook to the Homeobox Genes.

[CR30] Bürglin TR, Arai R, Kato M, Doi Y (1995). The evolution of homeobox genes. Biodiversity and evolution.

[CR31] Bürglin TR (1997). Analysis of TALE superclass homeobox genes (MEIS, PBC, KNOX, Iroquois, TGIF) reveals a novel domain conserved between plants and animals. Nucleic Acids Res.

[CR32] Bürglin TR (1998). The PBC domain contains a MEINOX domain: coevolution of Hox and TALE homeobox genes?. Dev Genes Evol.

[CR33] Bürglin TR (2005) Homeodomain proteins. In: Meyers RA (ed) Encyclopedia of Molecular Cell Biology and Molecular Medicine., vol 6. 2nd Edition edn. Wiley-VCH Verlag GmbH & Co., Weinheim, pp 179-222

[CR34] Bürglin TR (2011). Homeodomain subtypes and functional diversity. Subcell Biochem.

[CR35] Bürglin TR (2013a) Homeobox genes. In: Maloy S, Hughes K (eds) Brenner's Encyclopedia of Genetics, 2ed Academic Press, pp 503-508

[CR36] Bürglin TR (2013b) Homeotic mutations. In: Maloy S, Hughes K (eds) Brenner's Encyclopedia of Genetics, 2ed Academic Press, pp 510-511

[CR37] Bürglin TR, Cassata G (2002). Loss and gain of domains during evolution of cut superclass homeobox genes. Int J Dev Biol.

[CR38] Burri M, Tromvoukis Y, Bopp D, Frigerio G, Noll M (1989). Conservation of the paired domain in metazoans and its structure in three isolated human genes. EMBO J.

[CR39] Cande JD, Chopra VS, Levine M (2009). Evolving enhancer-promoter interactions within the tinman complex of the flour beetle, *Tribolium castaneum*. Development.

[CR40] Capellini TD, Zappavigna V, Selleri L (2011). Pbx homeodomain proteins: TALEnted regulators of limb patterning and outgrowth. Dev Dyn.

[CR41] Carrasco AE, McGinnis W, Gehring WJ, De Robertis EM (1984). Cloning of an *X. laevis* gene expressed during early embryogenesis coding for a peptide region homologous to *Drosophila* homeotic genes. Cell.

[CR42] Causier B, Ashworth M, Guo W, Davies B (2012). The TOPLESS interactome: a framework for gene repression in *Arabidopsis*. Plant Physiol.

[CR43] Chen G, Courey AJ (2000). Groucho/TLE family proteins and transcriptional repression. Gene.

[CR44] Chi YI, Frantz JD, Oh BC, Hansen L, Dhe-Paganon S, Shoelson SE (2002). Diabetes mutations delineate an atypical POU domain in HNF-1alpha. Mol Cell.

[CR45] Chourrout D, Delsuc F, Chourrout P, Edvardsen RB, Rentzsch F, Renfer E, Jensen MF, Zhu B, de Jong P, Steele RE (2006). Minimal ProtoHox cluster inferred from bilaterian and cnidarian Hox complements. Nature.

[CR46] Chu SW, Noyes MB, Christensen RG, Pierce BG, Zhu LJ, Weng Z, Stormo GD, Wolfe SA (2012). Exploring the DNA-recognition potential of homeodomains. Genome Res.

[CR47] Ciarbelli AR, Ciolfi A, Salvucci S, Ruzza V, Possenti M, Carabelli M, Fruscalzo A, Sessa G, Morelli G, Ruberti I (2008). The *Arabidopsis* homeodomain-leucine zipper II gene family: diversity and redundancy. Plant Mol Biol.

[CR48] Cinnamon E, Paroush Z (2008). Context-dependent regulation of Groucho/TLE-mediated repression. Curr Opin Genet Dev.

[CR49] Clarke M, Lohan AJ, Liu B, Lagkouvardos I, Roy S, Zafar N, Bertelli C, Schilde C, Kianianmomeni A, Bürglin TR (2013). Genome of *Acanthamoeba castellanii* highlights extensive lateral gene transfer and early evolution of tyrosine kinase signaling. Genome Biol.

[CR50] Copley RR (2005). The EH1 motif in metazoan transcription factors. BMC Genomics.

[CR51] Costanzo E, Trehin C, Vandenbussche M (2014). The role of *WOX* genes in flower development. Ann Bot.

[CR52] Crocker J, Abe N, Rinaldi L, McGregor AP, Frankel N, Wang S, Alsawadi A, Valenti P, Plaza S, Payre F (2015). Low affinity binding site clusters confer hox specificity and regulatory robustness. Cell.

[CR53] Crooks GE, Hon G, Chandonia JM, Brenner SE (2004). WebLogo: a sequence logo generator. Genome Res.

[CR54] de Mendoza A, Sebe-Pedros A, Sestak MS, Matejcic M, Torruella G, Domazet-Loso T, Ruiz-Trillo I (2013). Transcription factor evolution in eukaryotes and the assembly of the regulatory toolkit in multicellular lineages. Proc Natl Acad Sci U S A.

[CR55] De Robertis EM, Bürglin TR, Fritz A, Oliver G, Cho K, Wright CVE (1988). Sequence conservations in vertebrate homeo-box mRNAs. Arch Biol Med Exp.

[CR56] Derelle R, Lopez P, Le Guyader H, Manuel M (2007). Homeodomain proteins belong to the ancestral molecular toolkit of eukaryotes. Evol Dev.

[CR57] Deutsch JS (2010). Hox genes: studies from the 20th to the 21st century vol 689.

[CR58] Doerks T, Copley R, Bork P (2001). DDT – a novel domain in different transcription and chromosome remodeling factors. Trends Biochem Sci.

[CR59] Dong J, Gao Z, Liu S, Li G, Yang Z, Huang H, Xu L (2013). SLIDE, the protein interacting domain of Imitation Switch remodelers, binds DDT-domain proteins of different subfamilies in chromatin remodeling complexes. J Integr Plant Biol.

[CR60] Dozier C, Kagoshima H, Niklaus G, Cassata G, Bürglin TR (2001). The *Caenorhabditis elegans* Six/sine oculis class homeobox gene *ceh-32* is required for head morphogenesis. Dev Biol.

[CR61] Driever W, Nüsslein-Volhard C (1988). The *bicoid* protein determines position in the *Drosophila* embryo in a concentration-dependent manner. Cell.

[CR62] Dror I, Zhou T, Mandel-Gutfreund Y, Rohs R (2014). Covariation between homeodomain transcription factors and the shape of their DNA binding sites. Nucleic Acids Res.

[CR63] Duboule D (2007). The rise and fall of Hox gene clusters. Development.

[CR64] Duclercq J, Assoumou Ndong YP, Guerineau F, Sangwan RS, Catterou M (2011). *Arabidopsis* shoot organogenesis is enhanced by an amino acid change in the ATHB15 transcription factor. Plant Biol (Stuttg).

[CR65] Fan C, Chen Y, Long M (2008). Recurrent tandem gene duplication gave rise to functionally divergent genes in *Drosophila*. Mol Biol Evol.

[CR66] Feiner N, Meyer A, Kuraku S (2014). Evolution of the vertebrate Pax4/6 class of genes with focus on its novel member, the Pax10 gene. Genome Biol Evol.

[CR67] Feng JA, Johnson RC, Dickerson RE (1994) Hin recombinase bound to DNA: the origin of specificity in major and minor groove interactions. Science 263:348–355 10.1126/science.8278807 8278807

[CR68] Ferguson L, Marletaz F, Carter JM, Taylor WR, Gibbs M, Breuker CJ, Holland PW (2014). Ancient expansion of the hox cluster in lepidoptera generated four homeobox genes implicated in extra-embryonic tissue formation. PLoS Genet.

[CR69] Ferrier DE (2014). The Hox-TALE has been wagging for a long time. Elife.

[CR70] Finney M (1990). The homeodomain of the transcription factor LF-B1 has a 21 amino acid loop between helix 2 and helix 3. Cell.

[CR71] Fisher AL, Caudy M (1998). Groucho proteins: transcriptional corepressors for specific subsets of DNA-binding transcription factors in vertebrates and invertebrates. Genes Dev.

[CR72] Foos N, Maurel-Zaffran C, Mate MJ, Vincentelli R, Hainaut M, Berenger H, Pradel J, Saurin AJ, Ortiz-Lombardia M, Graba Y (2015). A flexible extension of the *Drosophila* ultrabithorax homeodomain defines a novel Hox/PBC interaction mode. Structure.

[CR73] Fortunato SA, Adamski M, Ramos OM, Leininger S, Liu J, Ferrier DE, Adamska M (2014). Calcisponges have a ParaHox gene and dynamic expression of dispersed NK homeobox genes. Nature.

[CR74] Frain M, Swart G, Monaci P, Nicosia A, Stämpfli S, Frank R, Cortese R (1989). The liver-specific transcription factor LF-B1 contains a highly diverged homeobox DNA binding domain. Cell.

[CR75] Friedrich M, Caravas J (2011). New insights from hemichordate genomes: prebilaterian origin and parallel modifications in the paired domain of the Pax gene *eyegone*. J Exp Zool B Mol Dev Evol.

[CR76] Furukawa T, Kozak CA, Cepko CL (1997). *rax*, a novel paired-type homeobox gene, shows expression in the anterior neural fold and developing retina. Proc Natl Acad Sci U S A.

[CR77] Galliot B, de Vargas C, Miller D (1999). Evolution of homeobox genes: Q50 Paired-like genes founded the Paired class. Dev Genes Evol.

[CR78] Garstang M, Ferrier DE (2013). Time is of the essence for ParaHox homeobox gene clustering. BMC Biol.

[CR79] Gehring WJ (2005). New perspectives on eye development and the evolution of eyes and photoreceptors. J Hered.

[CR80] Gehring WJ (2012). The animal body plan, the prototypic body segment, and eye evolution. Evol Dev.

[CR81] Gehring WJ (2014). The evolution of vision. Wiley Interdiscip Rev Dev Biol.

[CR82] Gehring WJ, Affolter M, Bürglin TR (1994). Homeodomain Proteins. Annu Rev Biochem.

[CR83] Gillingham AK, Pfeifer AC, Munro S (2002). CASP, the alternatively spliced product of the gene encoding the CCAAT-displacement protein transcription factor, is a Golgi membrane protein related to giantin. Mol Biol Cell.

[CR84] Goldstein RE, Cook O, Dinur T, Pisante A, Karandikar UC, Bidwai A, Paroush Z (2005). An eh1-like motif in odd-skipped mediates recruitment of Groucho and repression in vivo. Mol Cell Biol.

[CR85] Goriely A, Stella M, Coffinier C, Kessler D, Mailhos C, Dessain S, Desplan C (1996). A functional homologue of *goosecoid* in *Drosophila*. Development.

[CR86] Gouy M, Guindon S, Gascuel O (2010). SeaView version 4: A multiplatform graphical user interface for sequence alignment and phylogenetic tree building. Mol Biol Evol.

[CR87] Halder G, Callaerts P, Gehring WJ (1995). Induction of ectopic eyes by targeted expression of the *eyeless* gene in *Drosophila*. Science.

[CR88] Harvey RP (1996). *NK-2* homeobox genes and heart development. Dev Biol.

[CR89] Hay A, Tsiantis M (2010). KNOX genes: versatile regulators of plant development and diversity. Development.

[CR90] Hayakawa S, Takaku Y, Hwang JS, Horiguchi T, Suga H, Gehring W, Ikeo K, Gojobori T (2015). Function and evolutionary origin of unicellular camera-type eye structure. PLoS One.

[CR91] Hemmati-Brivanlou A, De la Torre JR, Holt C, Harland RM (1991). Cephalic expression and molecular characterization of *Xenopus En-2*. Development.

[CR92] Hench J, Henriksson J, Abou-Zied AM, Lüppert M, Dethlefsen J, Mukherjee K, Tong YG, Tang L, Gangishetti U, Baillie DL, Bürglin TR (2015) The Homeobox Genes of *Caenorhabditis elegans* and Insights into Their Spatio-Temporal Expression Dynamics during Embryogenesis. PLoS One 10 doi:ARTN e0126947 10.1371/journal.pone.0126947 10.1371/journal.pone.0126947 PMC4448998 26024448

[CR93] Herr W, Sturm RA, Clerc RG, Corcoran LM, Baltimore D, Sharp PA, Ingraham HA, Rosenfeld MG, Finney M, Ruvkun G (1988). The POU domain: a large conserved region in the mammalian *pit-1*, *oct-1*, *oct-2*, and *Caenorhabditis elegans unc-86* gene products. Genes Dev.

[CR94] Hill A, Boll W, Ries C, Warner L, Osswalt M, Hill M, Noll M (2010). Origin of Pax and Six gene families in sponges: Single *PaxB* and *Six1/2* orthologs in *Chalinula loosanoffi*. Dev Biol.

[CR95] Hobert O, Ruvkun G (1999). Pax genes in *Caenorhabditis elegans*: a new twist. Trends Genet.

[CR96] Hobert O, Westphal H (2000). Functions of LIM-homeobox genes. Trends Genet.

[CR97] Holland PW (2013). Evolution of homeobox genes. Wiley Interdiscip Rev Dev Biol.

[CR98] Holland PW (2015) Did homeobox gene duplications contribute to the Cambrian explosion? Zoological Letters 10.1186/s40851-014-0004-x PMC4604119 26605046

[CR99] Holland PW, Booth HA, Bruford EA (2007). Classification and nomenclature of all human homeobox genes. BMC Biol.

[CR100] Howard-Ashby M, Materna SC, Brown CT, Chen L, Cameron RA, Davidson EH (2006). Identification and characterization of homeobox transcription factor genes in *Strongylocentrotus purpuratus*, and their expression in embryonic development. Dev Biol.

[CR101] Hu W, dePamphilis CW, Ma H (2008). Phylogenetic analysis of the plant-specific *zinc finger-homeobox* and *mini zinc finger* gene families. J Integr Plant Biol.

[CR102] Huang S, Chen Z, Yan X, Yu T, Huang G, Yan Q, Pontarotti PA, Zhao H, Li J, Yang P (2014). Decelerated genome evolution in modern vertebrates revealed by analysis of multiple lancelet genomes. Nat Commun.

[CR103] Hudry B, Thomas-Chollier M, Volovik Y, Duffraisse M, Dard A, Frank D, Technau U, Merabet S (2014). Molecular insights into the origin of the Hox-TALE patterning system. Elife.

[CR104] Hui C-C, Matsuno K, Ueno K, Suzuki Y (1992). Molecular characterization and silk gland expression of *Bombyx engrailed* and *invected* genes. Proc Natl Acad Sci U S A.

[CR105] Hui JH, Holland PW, Ferrier DE (2008). Do cnidarians have a ParaHox cluster? Analysis of synteny around a *Nematostella* homeobox gene cluster. Evol Dev.

[CR106] Ikeda M, Mitsuda N, Ohme-Takagi M (2009). *Arabidopsis* WUSCHEL is a bifunctional transcription factor that acts as a repressor in stem cell regulation and as an activator in floral patterning. Plant Cell.

[CR107] Ikuta T (2011). Evolution of invertebrate deuterostomes and Hox/ParaHox genes. Genomics Proteomics Bioinformatics.

[CR108] Ivics Z, Izsvak Z, Minter A, Hackett PB (1996). Identification of functional domains and evolution of Tc1-like transposable elements. Proc Natl Acad Sci U S A.

[CR109] Iyaguchi D, Yao M, Watanabe N, Nishihira J, Tanaka I (2007). DNA recognition mechanism of the ONECUT homeodomain of transcription factor HNF-6. Structure.

[CR110] Jacob F (1977). Evolution and tinkering. Science.

[CR111] Jagla K, Bellard M, Frasch M (2001). A cluster of *Drosophila* homeobox genes involved in mesoderm differentiation programs. Bioessays.

[CR112] Jennings BH, Ish-Horowicz D (2008). The Groucho/TLE/Grg family of transcriptional co-repressors. Genome Biol.

[CR113] Jennings BH, Pickles LM, Wainwright SM, Roe SM, Pearl LH, Ish-Horowicz D (2006). Molecular recognition of transcriptional repressor motifs by the WD domain of the Groucho/TLE corepressor. Mol Cell.

[CR114] Jiao S, Tan X, Wang Q, Li M, Du SJ (2015). The olive flounder (*Paralichthys olivaceus*) *Pax3* homologues are highly conserved, encode multiple isoforms and show unique expression patterns. Comp Biochem Physiol B Biochem Mol Biol.

[CR115] Jiménez G, Paroush Z, Ish-Horowicz D (1997). Groucho acts as a corepressor for a subset of negative regulators, including Hairy and Engrailed. Genes Dev.

[CR116] Jiménez G, Verrijzer CP, Ish-Horowicz D (1999). A conserved motif in goosecoid mediates groucho-dependent repression in *Drosophila* embryos. Mol Cell Biol.

[CR117] Johnson M, Zaretskaya I, Raytselis Y, Merezhuk Y, McGinnis S, Madden TL (2008). NCBI BLAST: a better web interface. Nucleic Acids Res.

[CR118] Jolma A, Yan J, Whitington T, Toivonen J, Nitta KR, Rastas P, Morgunova E, Enge M, Taipale M, Wei G (2013). DNA-binding specificities of human transcription factors. Cell.

[CR119] Jones MH, Hamana N, Nezu J, Shimane M (2000). A novel family of bromodomain genes. Genomics.

[CR120] Joshi R, Passner JM, Rohs R, Jain R, Sosinsky A, Crickmore MA, Jacob V, Aggarwal AK, Honig B, Mann RS (2007). Functional specificity of a Hox protein mediated by the recognition of minor groove structure. Cell.

[CR121] Joyner AL, Hanks M (1991). The *engrailed* genes: evolution of function. Semin Dev Biol.

[CR122] Jun S, Desplan C (1996). Cooperative interactions between paired domain and homeodomain. Development.

[CR123] Kadrmas JL, Beckerle MC (2004). The LIM domain: from the cytoskeleton to the nucleus. Nat Rev Mol Cell Biol.

[CR124] Kagale S, Links MG, Rozwadowski K (2010). Genome-wide analysis of ethylene-responsive element binding factor-associated amphiphilic repression motif-containing transcriptional regulators in *Arabidopsis*. Plant Physiol.

[CR125] Kagale S, Rozwadowski K (2011). EAR motif-mediated transcriptional repression in plants: an underlying mechanism for epigenetic regulation of gene expression. Epigenetics.

[CR126] Kagoshima H, Cassata G, Bürglin TR (1999). A *Caenorhabditis elegans* homeobox gene expressed in the male tail, a link between pattern formation and sexual dimophism?. Dev Genes Evol.

[CR127] Kaul A, Schuster E, Jennings BH (2014). The Groucho co-repressor is primarily recruited to local target sites in active chromatin to attenuate transcription. PLoS Genet.

[CR128] Keller RG, Desplan C, Rosenberg MI (2010). Identification and characterization of *Nasonia* Pax genes. Insect Mol Biol.

[CR129] Kmita M, Duboule D (2003). Organizing axes in time and space; 25 years of colinear tinkering. Science.

[CR130] Koch BJ, Ryan JF, Baxevanis AD (2012). The diversification of the LIM superclass at the base of the metazoa increased subcellular complexity and promoted multicellular specialization. PLoS One.

[CR131] Koebernick K, Kashef J, Pieler T, Wedlich D (2006). *Xenopus Teashirt1* regulates posterior identity in brain and cranial neural crest. Dev Biol.

[CR132] Komachi K, Redd MJ, Johnson AD (1994). The WD repeats of Tup1 interact with the homeo domain protein alpha 2. Genes Dev.

[CR133] Kook H, Yung WW, Simpson RJ, Kee HJ, Shin S, Lowry JA, Loughlin FE, Yin Z, Epstein JA, Mackay JP (2006). Analysis of the structure and function of the transcriptional coregulator HOP. Biochemistry.

[CR134] Kumar JP (2009). The sine oculis homeobox (SIX) family of transcription factors as regulators of development and disease. Cell Mol Life Sci.

[CR135] Kuraku S, Meyer A (2009). The evolution and maintenance of Hox gene clusters in vertebrates and the teleost-specific genome duplication. Int J Dev Biol.

[CR136] Larkin MA, Blackshields G, Brown NP, Chenna R, McGettigan PA, McWilliam H, Valentin F, Wallace IM, Wilm A, Lopez R (2007). Clustal W and Clustal X version 2.0. Bioinformatics.

[CR137] Larroux C, Fahey B, Degnan SM, Adamski M, Rokhsar DS, Degnan BM (2007). The NK homeobox gene cluster predates the origin of Hox genes. Curr Biol.

[CR138] Laughon A, Scott MP (1984). Sequence of a *Drosophila* segmentation gene: protein structure homology with DNA-binding proteins. Nature.

[CR139] Laurent A, Bihan R, Omilli F, Deschamps S, Pellerin I (2008). PBX proteins: much more than Hox cofactors. Int J Dev Biol.

[CR140] Law JA, Du J, Hale CJ, Feng S, Krajewski K, Palanca AM, Strahl BD, Patel DJ, Jacobsen SE (2013). Polymerase IV occupancy at RNA-directed DNA methylation sites requires SHH1. Nature.

[CR141] Lee JH, Lin H, Joo S, Goodenough U (2008). Early sexual origins of homeoprotein heterodimerization and evolution of the plant KNOX/BELL family. Cell.

[CR142] Leidenroth A, Clapp J, Mitchell LM, Coneyworth D, Dearden FL, Iannuzzi L, Hewitt JE (2012). Evolution of *DUX* gene macrosatellites in placental mammals. Chromosoma.

[CR143] Leidenroth A, Hewitt JE (2010). A family history of *DUX4*: phylogenetic analysis of *DUXA*, *B*, *C* and *Duxbl* reveals the ancestral *DUX* gene. BMC Evol Biol.

[CR144] Letunic I, Doerks T, Bork P (2015). SMART: recent updates, new developments and status in 2015. Nucleic Acids Res.

[CR145] Levine M (2014). Retrospective. Walter Gehring (1939-2014). Science.

[CR146] Li T, Stark MR, Johnson AD, Wolberger C (1995). Crystal structure of the MATa1/MATa2 homeodomain heterodimer bound to DNA. Science.

[CR147] Lin H, Niu L, McHale NA, Ohme-Takagi M, Mysore KS, Tadege M (2013). Evolutionarily conserved repressive activity of WOX proteins mediates leaf blade outgrowth and floral organ development in plants. Proc Natl Acad Sci U S A.

[CR148] Lints TJ, Parsons LM, Hartley L, Lyons I, Harvey RP (1993). *Nkx-2.5*: a novel murine homeobox gene expressed in early heart progenitor cells and their myogenic descendants. Development.

[CR149] Liu Y, Ma D, Ji C (2015). Zinc fingers and homeoboxes family in human diseases. Cancer Gene Ther.

[CR150] Liu Y, Nandi S, Martel A, Antoun A, Ioshikhes I, Blais A (2012). Discovery, optimization and validation of an optimal DNA-binding sequence for the Six1 homeodomain transcription factor. Nucleic Acids Res.

[CR151] Liu Z, Karmarkar V (2008). Groucho/Tup1 family co-repressors in plant development. Trends Plant Sci.

[CR152] Logan C, Hanks MC, Noble-Topham S, Nallainathan D, Provart NJ, Joyner AL (1992). Cloning and sequence comparison of the mouse, human, and chicken *engrailed* genes reveal potential functional domains and regulatory regions. Dev Genet.

[CR153] Lonfat N, Duboule D (2015). Structure, Function and Evolution of Topologically Associating Domains (TADs) at Hox loci. FEBS Lett.

[CR154] Lonfat N, Montavon T, Darbellay F, Gitto S, Duboule D (2014). Convergent evolution of complex regulatory landscapes and pleiotropy at Hox loci. Science.

[CR155] Maclean JA, Chen MA, Wayne CM, Bruce SR, Rao M, Meistrich ML, Macleod C, Wilkinson MF (2005). *Rhox*: a new homeobox gene cluster. Cell.

[CR156] MacLean JA, Wilkinson MF (2010). The *Rhox* genes. Reproduction.

[CR157] Maeda RK, Karch F (2015). The *open for business* model of the bithorax complex in *Drosophila*. Chromosoma.

[CR158] Magnani E, Barton MK (2011). A per-ARNT-sim-like sensor domain uniquely regulates the activity of the homeodomain leucine zipper transcription factor REVOLUTA in *Arabidopsis*. Plant Cell.

[CR159] Malsam J, Satoh A, Pelletier L, Warren G (2005). Golgin tethers define subpopulations of COPI vesicles. Science.

[CR160] Mann RS, Affolter M (1998). Hox proteins meet more partners. Curr Opin Genet Dev.

[CR161] Mann RS, Lelli KM, Joshi R (2009). Hox specificity: unique roles for cofactors and collaborators. Curr Top Dev Biol.

[CR162] Mannervik M (2014). Control of *Drosophila* embryo patterning by transcriptional co-regulators. Exp Cell Res.

[CR163] Martin KJ, Holland PW (2014). Enigmatic orthology relationships between Hox clusters of the african butterfly fish and other teleosts following ancient whole-genome duplication. Mol Biol Evol.

[CR164] Mathelier A, Zhao X, Zhang AW, Parcy F, Worsley-Hunt R, Arenillas DJ, Buchman S, Chen CY, Chou A, Ienasescu H (2014). JASPAR 2014: an extensively expanded and updated open-access database of transcription factor binding profiles. Nucleic Acids Res.

[CR165] Mayran A, Pelletier A, Drouin J (2015). Pax factors in transcription and epigenetic remodelling. Semin Cell Dev Biol.

[CR166] Mazza ME, Pang K, Reitzel AM, Martindale MQ, Finnerty JR (2010). A conserved cluster of three PRD-class homeobox genes (*homeobrain*, *rx* and *orthopedia*) in the *Cnidaria* and *Protostomia*. Evodevo.

[CR167] McGinnis W, Garber RL, Wirz J, Kuroiwa A, Gehring WJ (1984). A homologous protein-coding sequence in *Drosophila* homeotic genes and its conservation in other metazoans. Cell.

[CR168] McGinnis W, Levine MS, Hafen E, Kuroiwa A, Gehring WJ (1984). A conserved DNA sequence in homoeotic genes of the *Drosophila* Antennapedia and bithorax complexes. Nature.

[CR169] Mendivil Ramos O, Barker D, Ferrier DE (2012). Ghost loci imply Hox and ParaHox existence in the last common ancestor of animals. Curr Biol.

[CR170] Merabet S, Galliot B (2015). The TALE face of Hox proteins in animal evolution. Front Genet.

[CR171] Merabet S, Hudry B (2013). Hox transcriptional specificity despite a single class of cofactors: are flexible interaction modes the key? Plasticity in Hox/PBC interaction modes as a common molecular strategy for shaping Hox transcriptional activities. Bioessays.

[CR172] Merabet S, Hudry B, Saadaoui M, Graba Y (2009). Classification of sequence signatures: a guide to Hox protein function. Bioessays.

[CR173] Merabet S, Lohmann I (2015). Toward a new twist in Hox and TALE DNA-binding specificity. Dev Cell.

[CR174] Mesika A, Ben-Dor S, Laviad EL, Futerman AH (2007). A new functional motif in Hox domain-containing ceramide synthases: identification of a novel region flanking the Hox and TLC domains essential for activity. J Biol Chem.

[CR175] Miller DJ, Hayward DC, Reece-Hoyes JS, Scholten I, Catmull J, Gehring WJ, Callaerts P, Larsen JE, Ball EE (2000). Pax gene diversity in the basal cnidarian *Acropora millepora* (*Cnidaria*, Anthozoa): implications for the evolution of the *Pax* gene family. Proc Natl Acad Sci U S A.

[CR176] Mitchell A, Chang HY, Daugherty L, Fraser M, Hunter S, Lopez R, McAnulla C, McMenamin C, Nuka G, Pesseat S (2015). The InterPro protein families database: the classification resource after 15 years. Nucleic Acids Res.

[CR177] Mizutani Y, Kihara A, Igarashi Y (2005). Mammalian Lass6 and its related family members regulate synthesis of specific ceramides. Biochem J.

[CR178] Mlodzik M, Halder G (2014). Walter J Gehring (1939-2014). Embo J.

[CR179] Mlodzik M, Halder G (2014). Walter J. Gehring (1939-2014). Dev Biol.

[CR180] Morino Y, Okada K, Niikura M, Honda M, Satoh N, Wada H (2013). A genome-wide survey of genes encoding transcription factors in the Japanese pearl oyster, *Pinctada fucata*: I. homeobox genes. Zool Sci.

[CR181] Muhr J, Andersson E, Persson M, Jessell TM, Ericson J (2001). Groucho-mediated transcriptional repression establishes progenitor cell pattern and neuronal fate in the ventral neural tube. Cell.

[CR182] Mukherjee K, Brocchieri L, Bürglin TR (2009). A comprehensive classification and evolutionary analysis of plant homeobox genes. Mol Biol Evol.

[CR183] Mukherjee K, Bürglin TR (2006). MEKHLA, a novel domain with similarity to PAS domains, is fused to plant homeodomain-leucine zipper III proteins. Plant Physiol.

[CR184] Mukherjee K, Bürglin TR (2007). Comprehensive Analysis of Animal TALE Homeobox Genes: New Conserved Motifs and Cases of Accelerated Evolution. J Mol Evol.

[CR185] Najafabadi HS, Mnaimneh S, Schmitges FW, Garton M, Lam KN, Yang A, Albu M, Weirauch MT, Radovani E, Kim PM (2015). C2H2 zinc finger proteins greatly expand the human regulatory lexicon. Nat Biotechnol.

[CR186] Nakamura M, Katsumata H, Abe M, Yabe N, Komeda Y, Yamamoto KT, Takahashi T (2006). Characterization of the class IV homeodomain-Leucine Zipper gene family in Arabidopsis. Plant Physiol.

[CR187] Negre B, Ruiz A (2007). HOM-C evolution in *Drosophila*: is there a need for Hox gene clustering?. Trends Genet.

[CR188] Noll M (1993). Evolution and role of *Pax* genes. Curr Opin Genet Dev.

[CR189] Noyes MB, Christensen RG, Wakabayashi A, Stormo GD, Brodsky MH, Wolfe SA (2008). Analysis of homeodomain specificities allows the family-wide prediction of preferred recognition sites. Cell.

[CR190] Ohta M, Matsui K, Hiratsu K, Shinshi H, Ohme-Takagi M (2001). Repression domains of class II ERF transcriptional repressors share an essential motif for active repression. Plant Cell.

[CR191] Oulion S, Bertrand S, Belgacem MR, Le Petillon Y, Escriva H (2012). Sequencing and analysis of the Mediterranean amphioxus (*Branchiostoma lanceolatum*) transcriptome. PLoS One.

[CR192] Papadopoulos DK, Skouloudaki K, Adachi Y, Samakovlis C, Gehring WJ (2012). Dimer formation via the homeodomain is required for function and specificity of Sex combs reduced in *Drosophila*. Dev Biol.

[CR193] Papizan JB, Singer RA, Tschen SI, Dhawan S, Friel JM, Hipkens SB, Magnuson MA, Bhushan A, Sussel L (2011). Nkx2.2 repressor complex regulates islet β-cell specification and prevents β-to-α-cell reprogramming. Genes Dev.

[CR194] Patrick AN, Cabrera JH, Smith AL, Chen XS, Ford HL, Zhao R (2013). Structure-function analyses of the human SIX1-EYA2 complex reveal insights into metastasis and BOR syndrome. Nat Struct Mol Biol.

[CR195] Pearson JC, Lemons D, McGinnis W (2005). Modulating Hox gene functions during animal body patterning. Nat Rev Genet.

[CR196] Peltenburg LT, Murre C (1996). Engrailed and Hox homeodomain proteins contain a related Pbx interaction motif that recognizes a common structure present in Pbx. Embo J.

[CR197] Pena PV, Davrazou F, Shi X, Walter KL, Verkhusha VV, Gozani O, Zhao R, Kutateladze TG (2006). Molecular mechanism of histone H3K4me3 recognition by plant homeodomain of ING2. Nature.

[CR198] Pérez-Bercoff Å, Bürglin TR (2010) LogoBar - Visualizing protein sequence logos with gaps. In: Fung GPC (ed) Sequence and Genome Analysis: Methods and Applications II. iConcept Press Ltd., Hong Kong, pp 57-70

[CR199] Pérez-Bercoff Å, Koch J, Bürglin TR (2006). LogoBar: bar graph visualization of protein logos with gaps. Bioinformatics.

[CR200] Pettersen EF, Goddard TD, Huang CC, Couch GS, Greenblatt DM, Meng EC, Ferrin TE (2004). UCSF Chimera - a visualization system for exploratory research and analysis. J Comput Chem.

[CR201] Pewzner-Jung Y, Ben-Dor S, Futerman AH (2006). When do Lasses (longevity assurance genes) become CerS (ceramide synthases)?: Insights into the regulation of ceramide synthesis. J Biol Chem.

[CR202] Pick L (2015) *Hox* genes, eve-devo and the case of the *ftz* gene. Chromosoma:in press 10.1007/s00412-015-0553-6 PMC4877300 26596987

[CR203] Punzo C, Kurata S, Gehring WJ (2001). The *eyeless* homeodomain is dispensable for eye development in *Drosophila*. Genes Dev.

[CR204] Purkayastha BP, Roy JK (2015). Cancer cell metabolism and developmental homeodomain/POU domain transcription factors: a connecting link. Cancer Lett.

[CR205] Putnam NH, Butts T, Ferrier DE, Furlong RF, Hellsten U, Kawashima T, Robinson-Rechavi M, Shoguchi E, Terry A, Yu JK (2008). The amphioxus genome and the evolution of the chordate karyotype. Nature.

[CR206] Quinonez SC, Innis JW (2014). Human HOX gene disorders. Mol Genet Metab.

[CR207] Quiring R, Walldorf U, Kloter U, Gehring WJ (1994). Homology of the *eyeless* gene of *Drosophila* to the *Small eye* gene in mice and *Aniridia* in humans. Science.

[CR208] Rajkovic A, Yan C, Yan W, Klysik M, Matzuk MM (2002). Obox, a family of homeobox genes preferentially expressed in germ cells. Genomics.

[CR209] Ratcliffe OJ, Riechmann JL (2002). *Arabidopsis* transcription factors and the regulation of flowering time: a genomic perspective. Curr Issues Mol Biol.

[CR210] Ravi V, Bhatia S, Gautier P, Loosli F, Tay BH, Tay A, Murdoch E, Coutinho P, van Heyningen V, Brenner S (2013). Sequencing of *Pax6* loci from the elephant shark reveals a family of *Pax6* genes in vertebrate genomes, forged by ancient duplications and divergences. PLoS Genet.

[CR211] Reményi A, Schöler HR, Wilmanns M (2004). Combinatorial control of gene expression. Nat Struct Mol Biol.

[CR212] Reményi A, Tomilin A, Pohl E, Lins K, Philippsen A, Reinbold R, Schöler HR, Wilmanns M (2001). Differential dimer activities of the transcription factor Oct-1 by DNA-induced interface swapping. Mol Cell.

[CR213] Rezsohazy R, Saurin AJ, Maurel-Zaffran C, Graba Y (2015). Cellular and molecular insights into Hox protein action. Development.

[CR214] Rohs R, West SM, Sosinsky A, Liu P, Mann RS, Honig B (2009). The role of DNA shape in protein-DNA recognition. Nature.

[CR215] Ronshaugen M, McGinnis N, McGinnis W (2002). Hox protein mutation and macroevolution of the insect body plan. Nature.

[CR216] Ryan JF, Burton PM, Mazza ME, Kwong GK, Mullikin JC, Finnerty JR (2006). The cnidarian-bilaterian ancestor possessed at least 56 homeoboxes. Evidence from the starlet sea anemone, *Nematostella vectensis*. Genome Biol.

[CR217] Ryan JF, Mazza ME, Pang K, Matus DQ, Baxevanis AD, Martindale MQ, Finnerty JR (2007). Pre-bilaterian origins of the Hox cluster and the Hox code: evidence from the sea anemone, *Nematostella vectensis*. PLoS One.

[CR218] Ryan JF, Pang K, Program NCS, Mullikin JC, Martindale MQ, Baxevanis AD (2010). The homeodomain complement of the ctenophore *Mnemiopsis leidyi* suggests that *Ctenophora* and *Porifera* diverged prior to the ParaHoxozoa. Evodevo.

[CR219] Ryter JM, Doe CQ, Matthews BW (2002). Structure of the DNA binding region of prospero reveals a novel homeo-prospero domain. Structure (Camb).

[CR220] Santos JS, Fonseca NA, Vieira CP, Vieira J, Casares F (2010). Phylogeny of the teashirt-related zinc finger (tshz) gene family and analysis of the developmental expression of *tshz2* and *tshz3b* in the zebrafish. Dev Dyn.

[CR221] Schier AF (2014). Obituary: Walter J. Gehring (1939-2014). Development.

[CR222] Schrick K, Bruno M, Khosla A, Cox PN, Marlatt SA, Roque RA, Nguyen HC, He C, Snyder MP, Singh D (2014). Shared functions of plant and mammalian StAR-related lipid transfer (START) domains in modulating transcription factor activity. BMC Biol.

[CR223] Schrick K, Nguyen D, Karlowski WM, Mayer KF (2004). START lipid/sterol-binding domains are amplified in plants and are predominantly associated with homeodomain transcription factors. Genome Biol.

[CR224] Schulte D, Frank D (2014). TALE transcription factors during early development of the vertebrate brain and eye. Dev Dyn.

[CR225] Scott MP, Weiner AJ (1984). Structural relationships among genes that control development: Sequence homology between the *Antennapedia*, *Ultrabithorax*, and *fushi tarazu* loci in *Drosophila*. Proc Natl Acad Sci U S A.

[CR226] Seifert A, Werheid DF, Knapp SM, Tobiasch E (2015). Role of Hox genes in stem cell differentiation. World J Stem Cells.

[CR227] Shimeld SM (1997). A transcriptional modification motif encoded by homeobox and fork head genes. FEBS Lett.

[CR228] Simakov O, Marletaz F, Cho SJ, Edsinger-Gonzales E, Havlak P, Hellsten U, Kuo DH, Larsson T, Lv J, Arendt D (2013). Insights into bilaterian evolution from three spiralian genomes. Nature.

[CR229] Sivanantharajah L, Percival-Smith A (2015). Differential pleiotropy and HOX functional organization. Dev Biol.

[CR230] Slattery M, Riley T, Liu P, Abe N, Gomez-Alcala P, Dror I, Zhou T, Rohs R, Honig B, Bussemaker HJ (2011). Cofactor binding evokes latent differences in DNA binding specificity between Hox proteins. Cell.

[CR231] Smith RL, Johnson AD (2000). Turning genes off by Ssn6-Tup1: a conserved system of transcriptional repression in eukaryotes. Trends Biochem Sci.

[CR232] Smith ST, Jaynes JB (1996). A conserved region of engrailed, shared among all en-, gsc-, Nk1-, Nk2- and msh-class homeoproteins, mediates active transcriptional repression in vivo. Development.

[CR233] Srivastava M, Begovic E, Chapman J, Putnam NH, Hellsten U, Kawashima T, Kuo A, Mitros T, Salamov A, Carpenter ML (2008). The *Trichoplax* genome and the nature of placozoans. Nature.

[CR234] Srivastava M, Larroux C, Lu DR, Mohanty K, Chapman J, Degnan BM, Rokhsar DS (2010). Early evolution of the LIM homeobox gene family. BMC Biol.

[CR235] Srivastava M, Simakov O, Chapman J, Fahey B, Gauthier ME, Mitros T, Richards GS, Conaco C, Dacre M, Hellsten U (2010). The *Amphimedon queenslandica* genome and the evolution of animal complexity. Nature.

[CR236] Stauber M, Jäckle H, Schmidt-Ott U (1999). The anterior determinant *bicoid* of *Drosophila* is a derived *Hox* class 3 gene. Proc Natl Acad Sci U S A.

[CR237] Stauber M, Prell A, Schmidt-Ott U (2002). A single *Hox3* gene with composite *bicoid* and *zerknüllt* expression characteristics in non-Cyclorrhaphan flies. Proc Natl Acad Sci U S A.

[CR238] Suga H, Tschopp P, Graziussi DF, Stierwald M, Schmid V, Gehring WJ (2010). Flexibly deployed *Pax* genes in eye development at the early evolution of animals demonstrated by studies on a hydrozoan jellyfish. Proc Natl Acad Sci U S A.

[CR239] Takatori N, Butts T, Candiani S, Pestarino M, Ferrier DE, Saiga H, Holland PW (2008). Comprehensive survey and classification of homeobox genes in the genome of amphioxus, *Branchiostoma floridae*. Dev Genes Evol.

[CR240] Takatori N, Saiga H (2008). Evolution of CUT class homeobox genes: insights from the genome of the amphioxus, *Branchiostoma floridae*. Int J Dev Biol.

[CR241] Te Velthuis AJ, Isogai T, Gerrits L, Bagowski CP (2007). Insights into the molecular evolution of the PDZ/LIM family and identification of a novel conserved protein motif. PLoS One.

[CR242] Töhönen V, Katayama S, Vesterlund L, Jouhilahti E-M, Sheikhi M, Madissoon E, Filippini-Cattaneo G, Jaconi M, Johnsson A, Bürglin TR et al. (2015) Novel PRD-like homeodomain transcription factors and retrotransposon elements in early human development. Nat Commun 6:doi:10.1038/ncomms9207 10.1038/ncomms9207 PMC4569847 26360614

[CR243] Tour E, Hittinger CT, McGinnis W (2005). Evolutionarily conserved domains required for activation and repression functions of the *Drosophila* Hox protein Ultrabithorax. Development.

[CR244] Tron AE, Bertoncini CW, Chan RL, Gonzalez DH (2002). Redox regulation of plant homeodomain transcription factors. J Biol Chem.

[CR245] Tsai IJ, Zarowiecki M, Holroyd N, Garciarrubio A, Sanchez-Flores A, Brooks KL, Tracey A, Bobes RJ, Fragoso G, Sciutto E (2013). The genomes of four tapeworm species reveal adaptations to parasitism. Nature.

[CR246] Tsuda K, Hake S (2015). Diverse functions of KNOX transcription factors in the diploid body plan of plants. Curr Opin Plant Biol.

[CR247] Turki-Judeh W, Courey AJ (2012). Groucho: a corepressor with instructive roles in development. Curr Top Dev Biol.

[CR248] Underhill DA (2012). PAX proteins and fables of their reconstruction. Crit Rev Eukaryot Gene Expr.

[CR249] van der Graaff E, Laux T, Rensing SA (2009). The WUS homeobox-containing (WOX) protein family. Genome Biol.

[CR250] Viola IL, Gonzalez DH (2015) Structure and evolution of plant homeobox genes. In: Gonzalez DH (ed) Plant Transcription Factors. Evolutionary, Structural and Functional Aspects. Academic Press, Elsevier, pp 101-112

[CR251] Vollmer J-Y, Clerc RG (1998). Homeobox genes in the developing mouse brain. J Neurochem.

[CR252] Vorobyov E, Horst J (2006). Getting the proto-Pax by the tail. J Mol Evol.

[CR253] Wang X, He C, Hu X (2014). LIM homeobox transcription factors, a novel subfamily which plays an important role in cancer (review). Oncol Rep.

[CR254] Wang YT, Pan YJ, Cho CC, Lin BC, Su LH, Huang YC, Sun CH (2010). A novel pax-like protein involved in transcriptional activation of cyst wall protein genes in *Giardia lamblia*. J Biol Chem.

[CR255] Wang Z, Yang X, Chu X, Zhang J, Zhou H, Shen Y, Long J (2012). The structural basis for the oligomerization of the N-terminal domain of SATB1. Nucleic Acids Res.

[CR256] Wang Z, Yang X, Guo S, Yang Y, Su XC, Shen Y, Long J (2014). Crystal structure of the ubiquitin-like domain-CUT repeat-like tandem of special AT-rich sequence binding protein 1 (SATB1) reveals a coordinating DNA-binding mechanism. J Biol Chem.

[CR257] Wieschaus E, Nüsslein-Volhard C (2014). Walter Gehring (1939-2014). Curr Biol.

[CR258] Winnier AR, Meir JY, Ross JM, Tavernarakis N, Driscoll M, Ishihara T, Katsura I, Miller DM (1999). UNC-4/UNC-37-dependent repression of motor neuron-specific genes controls synaptic choice in *Caenorhabditis elegans*. Genes Dev.

[CR259] Wong KH, Struhl K (2011). The Cyc8-Tup1 complex inhibits transcription primarily by masking the activation domain of the recruiting protein. Genes Dev.

[CR260] Xiong D, Wang Y, Deng C, Hu R, Tian C (2015). Phylogenic analysis revealed an expanded C_2_H_2_-homeobox subfamily and expression profiles of C_2_H_2_ zinc finger gene family in *Verticillium dahliae*. Gene.

[CR261] Xu HE, Rould MA, Xu W, Epstein JA, Maas RL, Pabo CO (1999). Crystal structure of the human Pax6 paired domain-DNA complex reveals specific roles for the linker region and carboxy-terminal subdomain in DNA binding. Genes Dev.

[CR262] Xu W, Rould MA, Jun S, Desplan C, Pabo CO (1995). Crystal structure of a paired domain-DNA complex at 2.5Å resolution reveals structural basis for Pax developmental mutations. Cell.

[CR263] Yaklichkin S, Vekker A, Stayrook S, Lewis M, Kessler DS (2007). Prevalence of the EH1 Groucho interaction motif in the metazoan Fox family of transcriptional regulators. BMC Genomics.

[CR264] Yamasaki K, Akiba T, Yamasaki T, Harata K (2007). Structural basis for recognition of the matrix attachment region of DNA by transcription factor SATB1. Nucleic Acids Res.

[CR265] Yang L, Zhou T, Dror I, Mathelier A, Wasserman WW, Gordan R, Rohs R (2014). TFBSshape: a motif database for DNA shape features of transcription factor binding sites. Nucleic Acids Res.

[CR266] Young RA (2011). Control of the embryonic stem cell state. Cell.

[CR267] Zagozewski JL, Zhang Q, Pinto VI, Wigle JT, Eisenstat DD (2014). The role of homeobox genes in retinal development and disease. Dev Biol.

[CR268] Zakany J, Duboule D (2007). The role of Hox genes during vertebrate limb development. Curr Opin Genet Dev.

[CR269] Zhang Y, Emmons SW (1995). Specification of sense-organ identity by a *Caenorhabditis elegans Pax-6* homologue. Nature.

[CR270] Zheng Q, Zhao Y (2007). The diverse biofunctions of LIM domain proteins: determined by subcellular localization and protein-protein interaction. Biol Cell.

[CR271] Zhong YF, Holland PW (2011). The dynamics of vertebrate homeobox gene evolution: gain and loss of genes in mouse and human lineages. BMC Evol Biol.

[CR272] Zhong YF, Holland PW (2011). HomeoDB2: functional expansion of a comparative homeobox gene database for evolutionary developmental biology. Evol Dev.

