# Supplementary figures and images for: Homeodomain proteins: an update
Source: Chromosoma. 2015 Oct 13;125:497–521. doi: 10.1007/s00412-015-0543-8 (PMC4901127; doi:10.1007/s00412-015-0543-8)

Sup. Fig. S1 Multiple sequence alignment of HDs

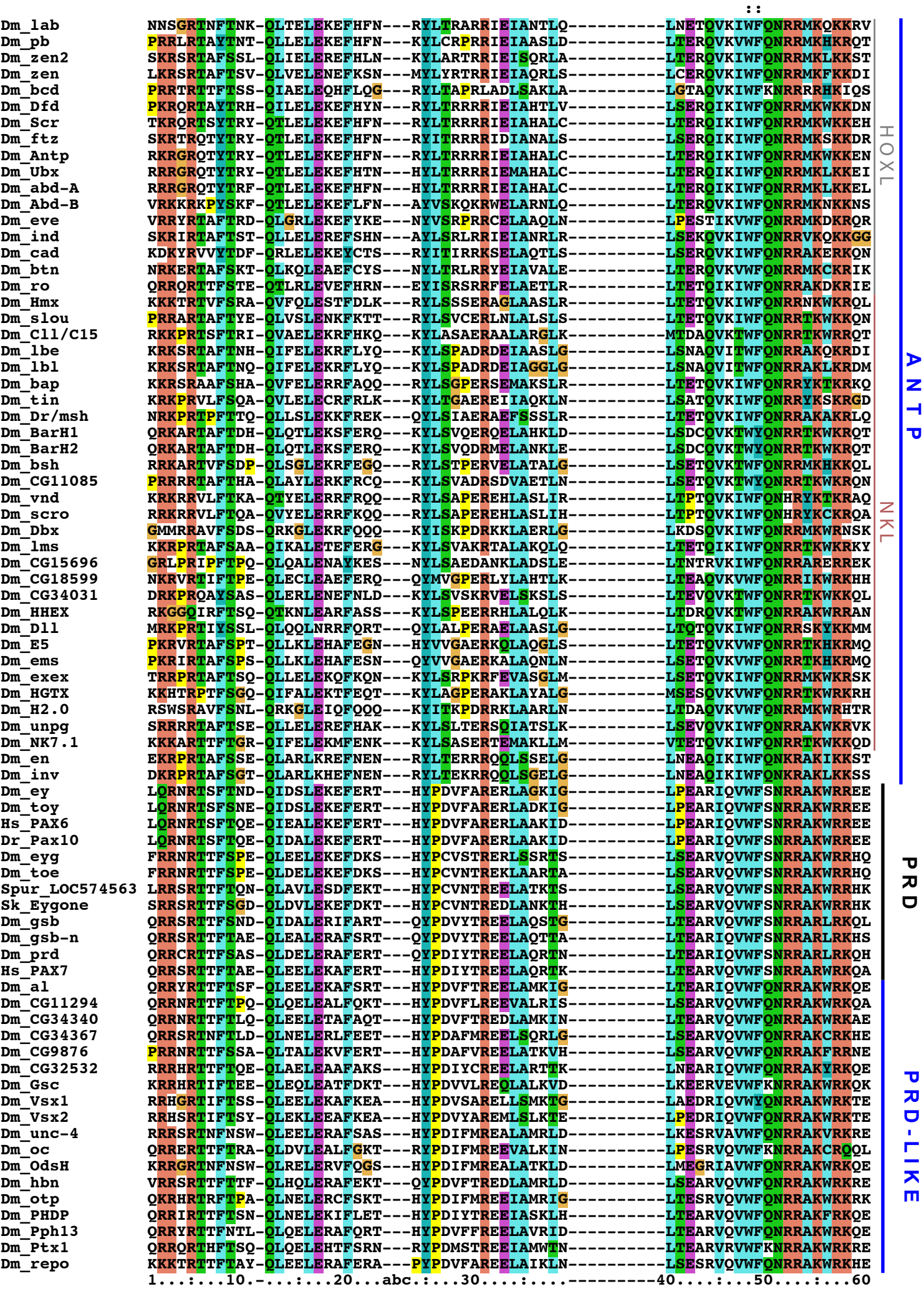

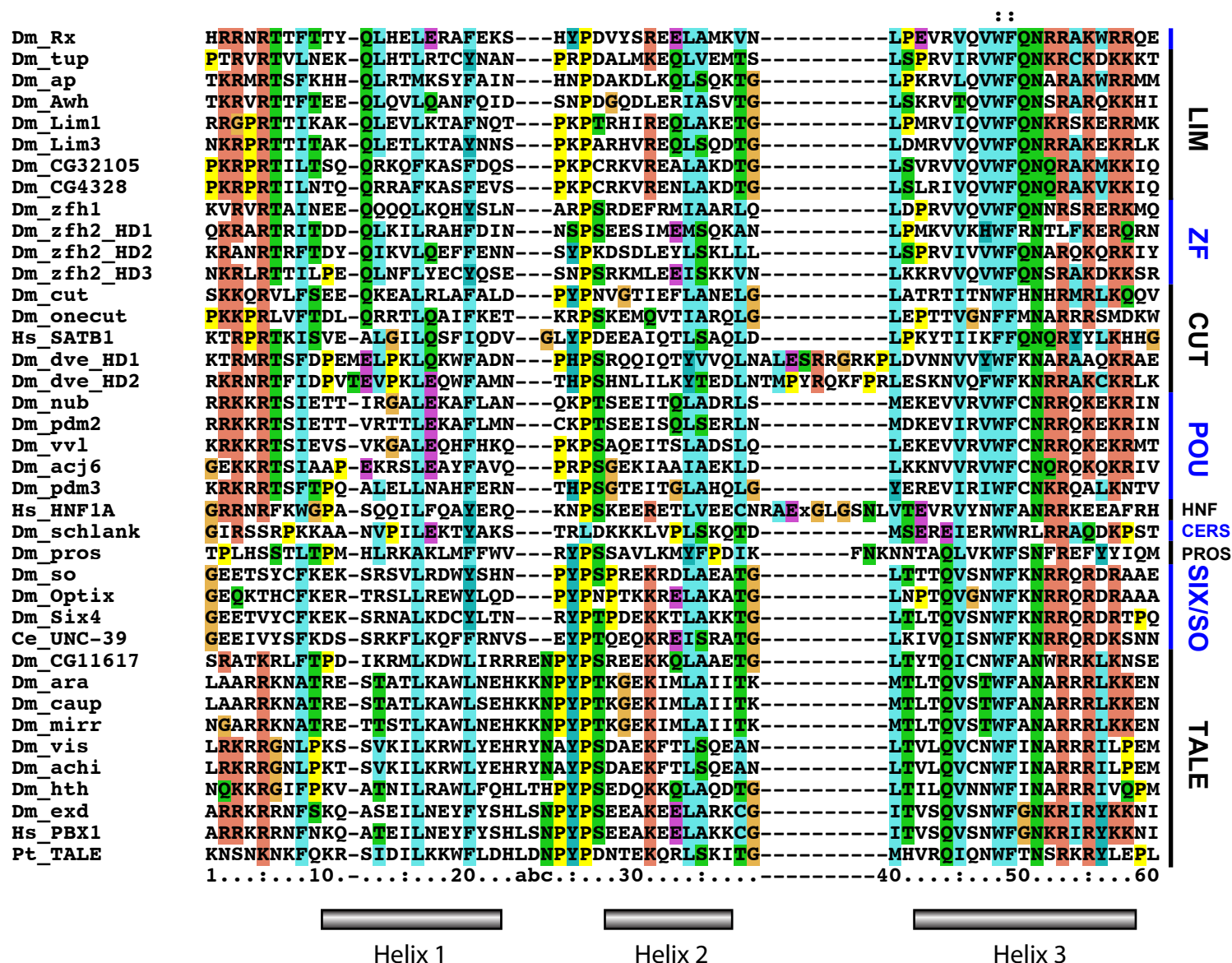

Supplement: Supplementary file 1 — Multiple sequence alignment of 103 Drosophila melanogaster HDs, supplemented with a select few additional HDs from other species. Sequences were collected from HomeoDB (Zhong and Holland 2011b), corrected, and updated. The Drosophila Shaven protein was excluded, as it lacks the partial Pax2/5/8 HD of vertebrates (Sup. Fig. S5). An extra gap was introduced upstream of helix 1 that gives a better alignment at the N-terminus for the two Dve HDs. In human HNF1A twelve residues were omitted at the 'x' in the second loop. The default color code of Clustal X was used (Larkin et al. 2007); it colors conserved residues with similar properties. Species abbreviations: Dm: Drosophila melanogaster; Hs: human; Dr: Danio rerio (zebrafish); Spur: Strongylocentrotus purpuratus (purple sea urchin); Sk: Saccoglossus kowalevskii (acorn worm; hemichordate); Ce: Caenorhabditis elegans; Pt: Paramecium tetraurelia (sequence accession number: XP_001455625). (PDF 3.03 MB) [file 412_2015_543_MOESM1_ESM.pdf]

**Sup. Fig. S2.** Multiple sequence alignment of fungal mating type alpha2 HD proteins.

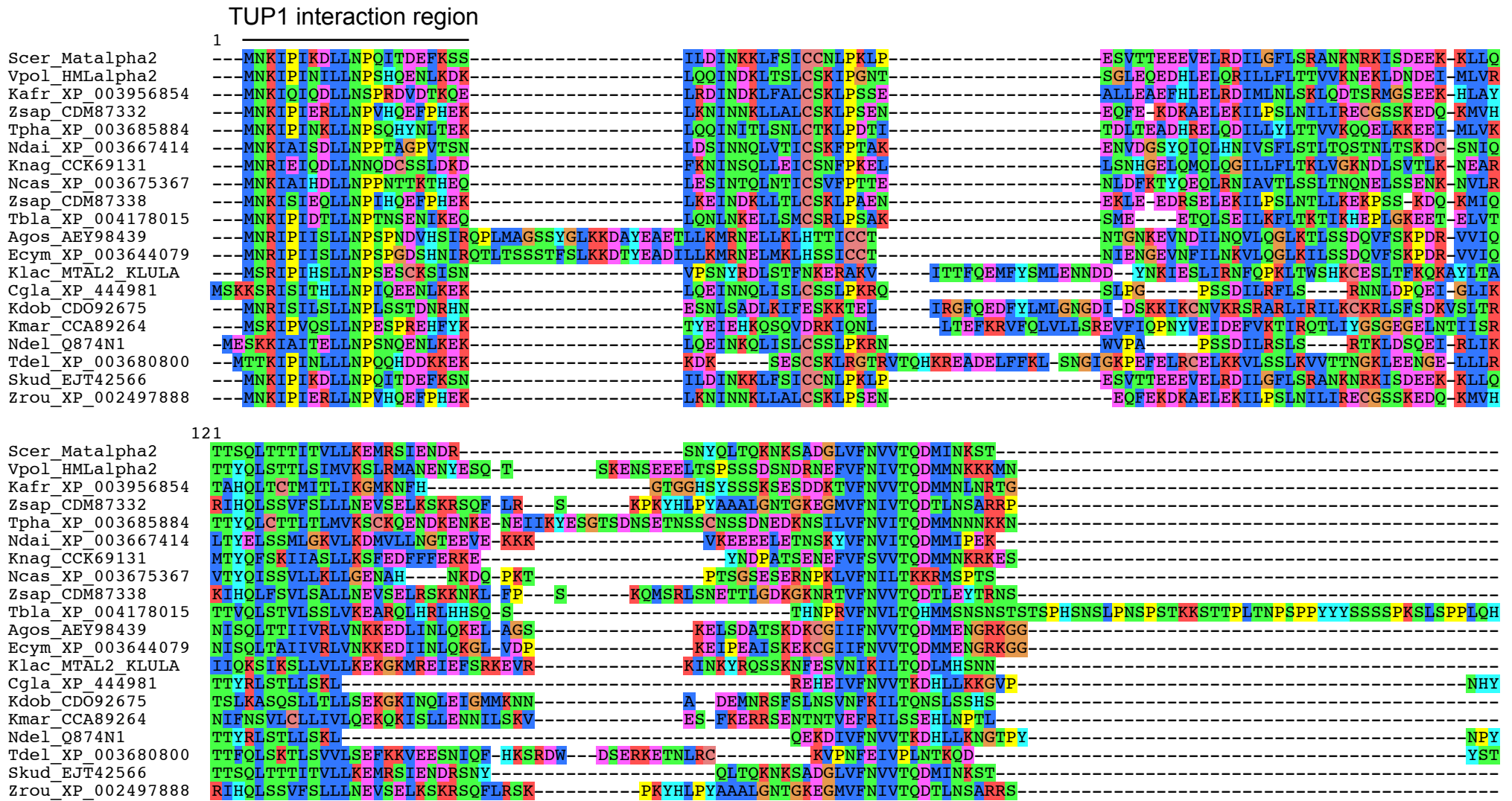

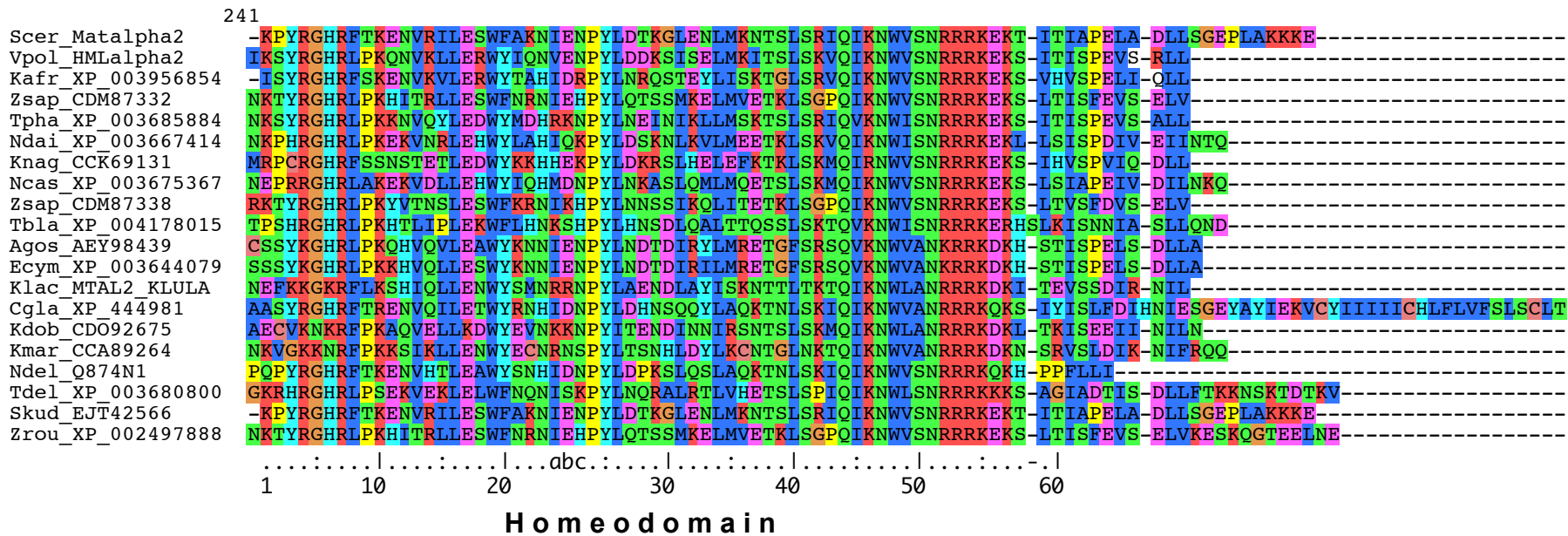

Supplement: Supplementary file 2 — Multiple sequence alignment of fungal MATα2 proteins. Default color code from SeaView (Gouy et al. 2010). Species abbreviations: Scer: Saccharomyces cerevisiae; Vpol: Vanderwaltozyma polyspora; Kafr: Kazachstania africana; Zsap: Zygosaccharomyces sapae; Tpha: Tetrapisispora phaffii; Ndai: Naumovozyma dairenensis; Knag: Kazachstania naganishii; Ncas: Naumovozyma castellii; Tbla: Tetrapisispora blattae; Agos: Ashbya gossypii; Ecym: Eremothecium cymbalariae; Klac: Kluyveromyces lactis; Cgla: Candida glabrata; Kdob: Kluyveromyces dobzhanskii; Kmar: Kluyveromyces marxianus; Ndel: Nakaseomyces delphensis; Tdel: Torulaspora delbrueckii; Skud: Saccharomyces kudriavzevii; Zrou: Zygosaccharomyces rouxii. (PDF 395 kb) [file 412_2015_543_MOESM2_ESM.pdf]

**Sup. Fig. S4** 3D crystal structure of the HDs of yeast MAT $\alpha$ 2 and MAT $\alpha$ 1 with water molecules.

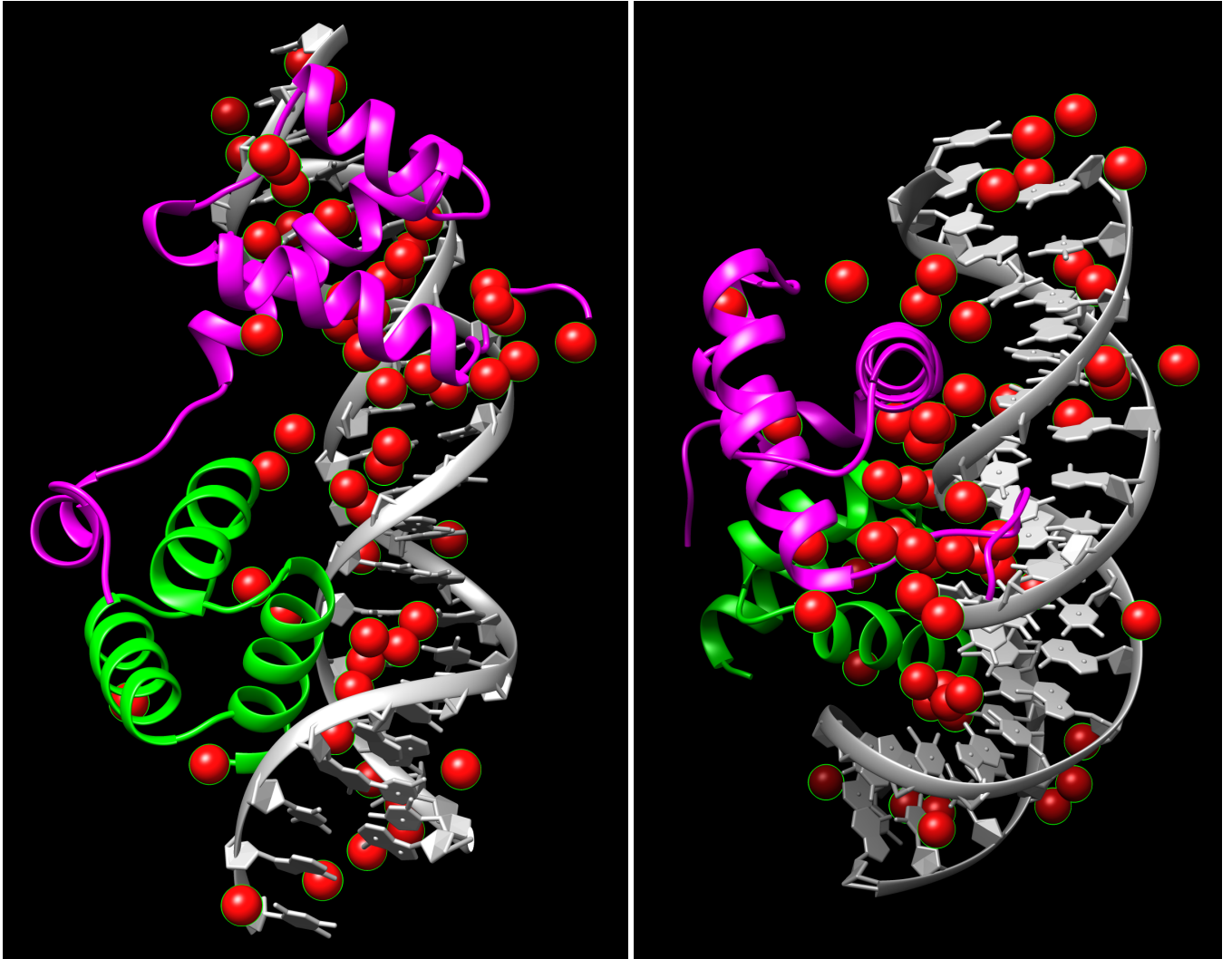

Supplement: Supplementary file 4 — 3D crystal structure of the HDs of yeast MATα2 and MATa1 bound to DNA as in Fig. 5a, with the addition of the water molecules, which are visualized as red spheres. Left panel: same perspective as in Fig. 5a. Right panel: rotated to provide a side view of the third helix of MATα2 with the water molecules in the major groove. (PDF 2.73 MB) [file 412_2015_543_MOESM4_ESM.pdf]
