# Supplementary material for: Homeodomain proteins: an update
Source: Chromosoma. 2015 Oct 13;125:497–521. doi: 10.1007/s00412-015-0543-8 (PMC4901127; doi:10.1007/s00412-015-0543-8)
Supplement: Supplementary file 3 — Schematic domain organization of ZF HD proteins. Human (h), selected Drosophila, and amphioxus ZF class HD proteins are shown schematically using the output from the SMART domain server (Letunic et al. 2015) with some manual corrections. The HOMEZ gene was initially named based on two putative leucine zippers encoded in the mammalian genes (Bayarsaihan et al. 2003). However, these predicted zippers are not conserved in fish, and SMART (Letunic et al. 2015) as well as Interpro (Mitchell et al. 2015) do not identify them as zippers, leaving their functional significance in doubt. (PDF 6.92 MB) [file 412_2015_543_MOESM3_ESM.pdf]

ZF family	Name	Protein structure
**Adnp**	h ADNP	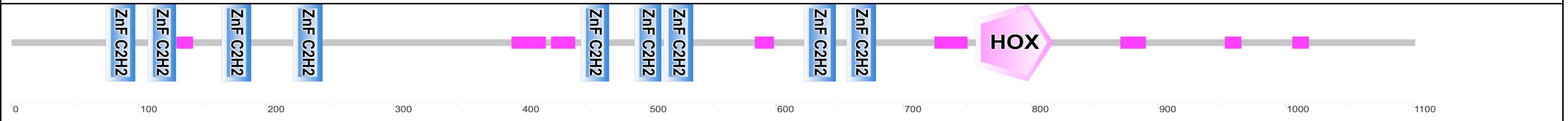
	h ADNP2	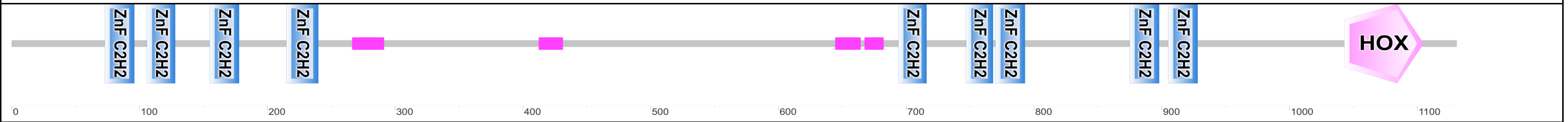
**Tshz**	h TSHZ1	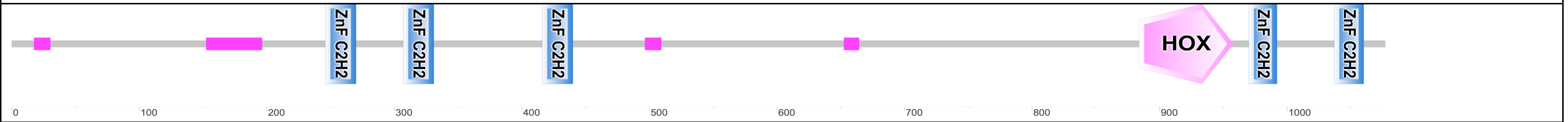
	h TSHZ2	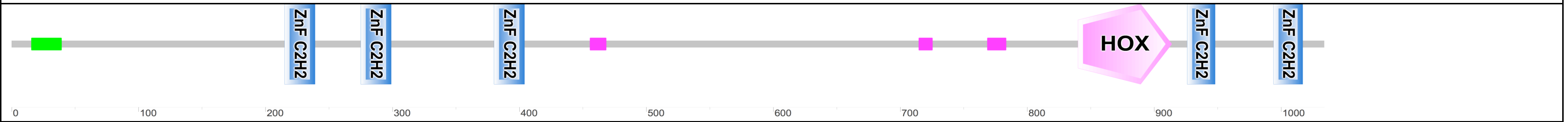
	h TSHZ3	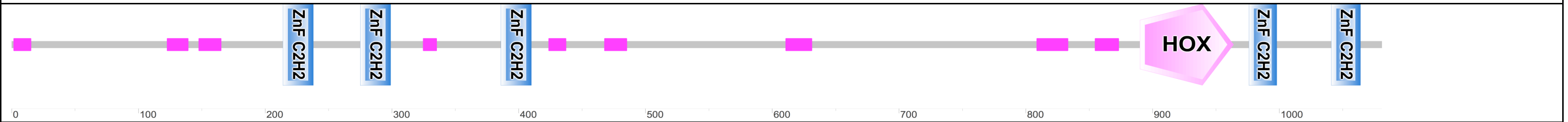
	Drosophila Tsh (no HD)	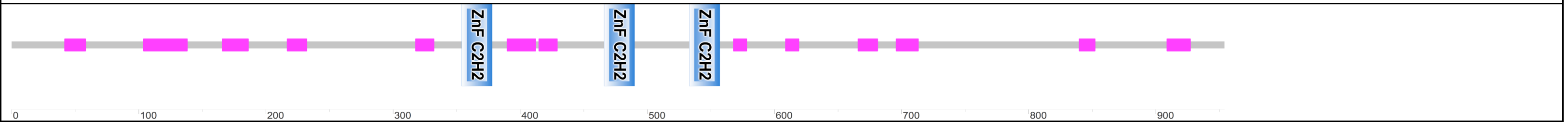
**Zeb**	h ZEB1	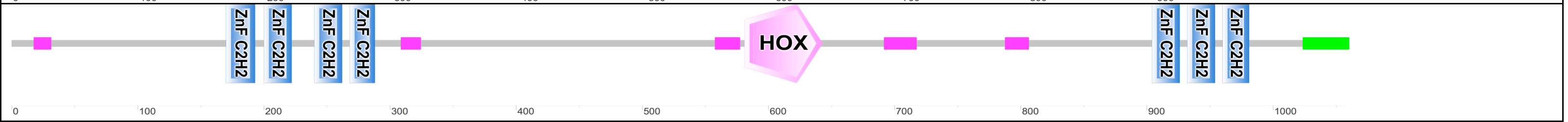
	h ZEB2	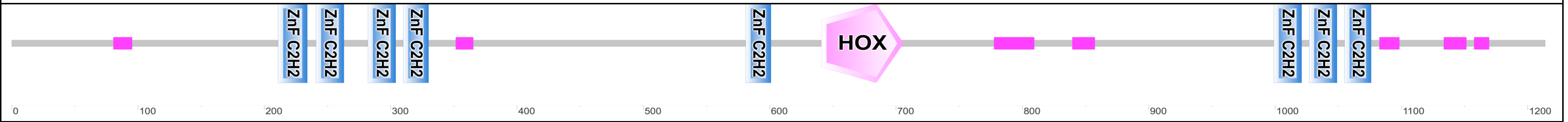
	Drosophila Zfh1	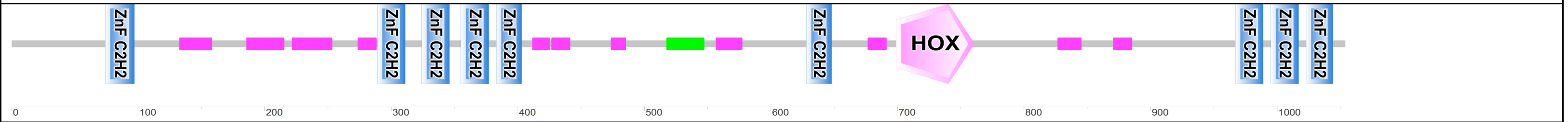

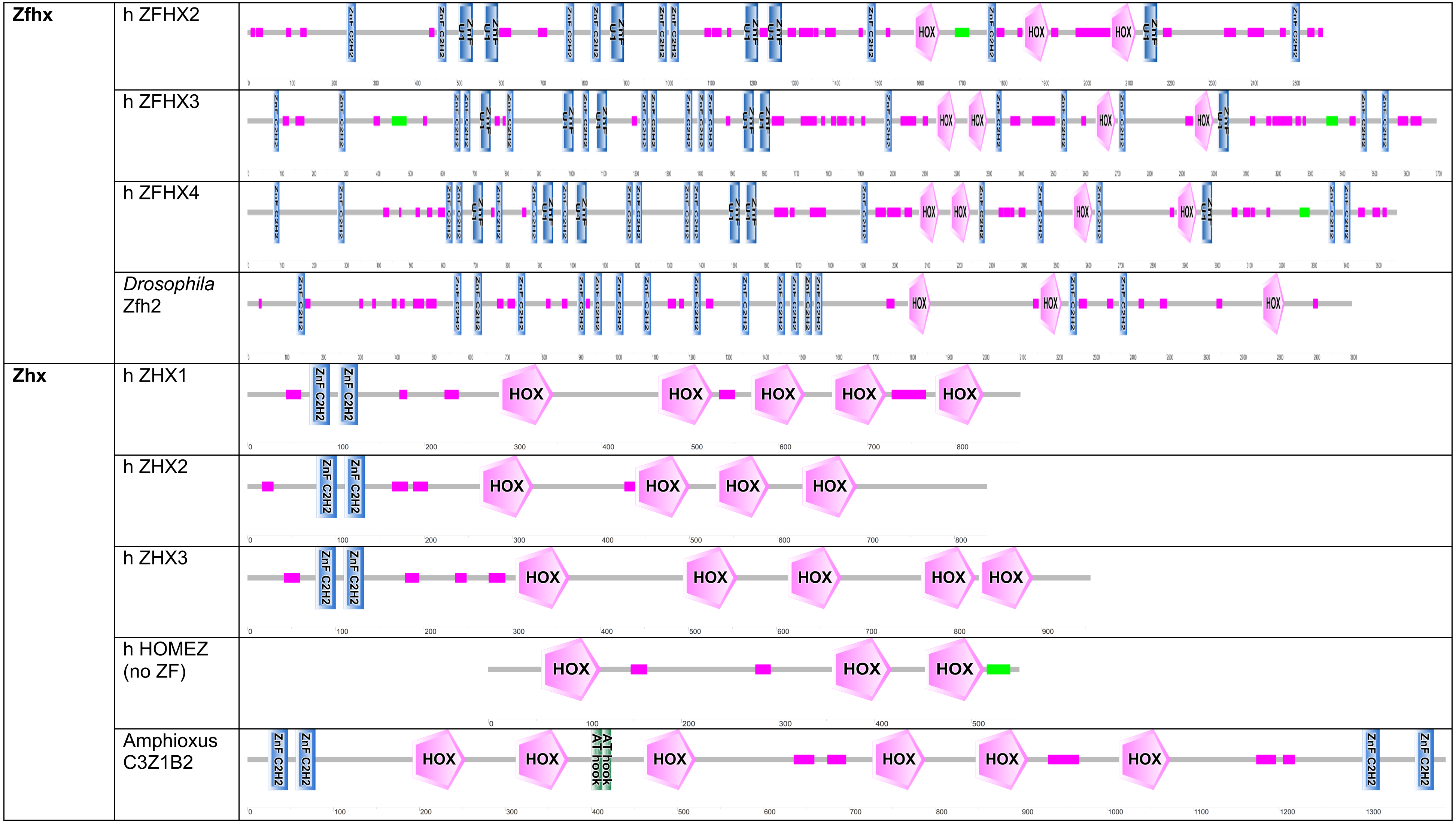

Sup. Fig. S3 Schematic domain organization of ZF HD proteins.

Homeodomain proteins: an update. Chromosoma, Bürglin, TR, and Affolter, M. Dept. of Biomedicine, University of Basel. email: thomas.buerglin@unibas.ch
