# Supplementary material for: Homeodomain proteins: an update
Source: Chromosoma. 2015 Oct 13;125:497–521. doi: 10.1007/s00412-015-0543-8 (PMC4901127; doi:10.1007/s00412-015-0543-8)
Supplement: Supplementary file 5 — Sequence alignment of selected PRD class proteins generated with SeaView (Gouy et al. 2010) and Clustal X (Larkin et al. 2007). Sequences were extracted from Genbank after a blastp search using a PAIRED domain as seed (Johnson et al. 2008). The different domains are marked. Manual sequence alignment for the shorter motifs was necessary; hence sequence alignments outside the indicated motifs may not be optimal. Furthermore, the usual caveats apply, i.e. ORFs derived from genome projects may contain errors due to mistakes in the initial sequence or assembly, and/or in the subsequent ORF prediction and annotation. Note that the OAR motif in the C-terminal region of Bf_Pax2/5/8, as proposed by (Vorobyov and Horst 2006), is not present. Our analysis of the C-terminal Pax2/5/8 sequences of Branchiostoma floridae (Putnam et al. 2008), B. belcheri (Huang et al. 2014), and B. lanceolatum (Oulion et al. 2012) did not show the proposed frame shift that would be required to place the out-of-frame OAR-like motif at the C-terminus of Pax2/5/8; the sequence similarity may be fortuitous. A PRD class sequence from oyster (Cg) does have an OAR motif matching vertebrate PAX7 proteins. As an update to the previous publication on NPAX genes (Hobert and Ruvkun 1999), we note that new transcript data of NPAX-2 reveal that it also encodes a divergent RED domain, and that NPAX-1 and NPAX-4 encode an EH1 motif, which is conserved in other nematodes (e.g., Ancylostoma ceylanicum). Pax-6 proteins from flies and human, but not vertebrate Pax-4 proteins, contain a conserved motif (marked PAX6) between the PAIRED domain and the HD. It has been proposed that this motif is reminiscent of the EH1 motif (Keller et al. 2010). However, we propose a different, shifted alignment that would take the key hydrophobic residues better into consideration with respect to the EH1 profile. In our case, the D.m. Eyeless sequence “YEKLRLL” would align with the EH1 consensus “YSINGIL”. In this alignment the hydropho [file 412_2015_543_MOESM5_ESM.pdf]

Sup. Fig. S5 Sequence alignment of selected PRD class proteins

```
                    1
Ce_NPAX-3           -----------------------------------------------------------------------------------------------------MLDVHCCDHEQCGSSVIDLSTPHTLQRDFNNWLNGIEVP
Ce_NPAX-4           --------------------------------------------------------------------------------------------------------MMSPSIFPPINFLFQ-NSALLSSFAAQKALSNGHPS
Acey_NPAX-4         ---------------------------------------------------------------------------------------------------------MHPAAAQLQQFFLPRMSSTYENDLAMRLQADATTP
Ce_NPAX-1           ----------------------------------------------------------------------------------------------------------------------------MLPQIHQHTHFPVSTN
Ce_NPAX-2           -----------------------------------------------------------------------------------MDEPVIDTSTPESLERDFMAWLNSDPTNTPIRKSVYHSSDSIAPALISQFTDAFEAG
Dm_Poxn             --------------------------------------------------------------------------------------------------------------------------------------------
Md_Poxn             --------------------------------------------------------------------------------------------------------------------------------------------
Cc_Poxn             --------------------------------------------------------------------------------------------------------------------------------------------
Sk_Poxn             --------------------------------------------------------------------------------------------------------------------------------------------
Ac_Poxn             --------------------------------------------------------------------------------------------------------------------------------------------
Cg_EKC37735         --------------------------------------------------------------------------------------------------------------------------------------------
Hs_PAX1             ----------------------------------------------------------------------------------MKFTLGLGSRAWRVSWEGAAAAAAGPGAGGSALRCRAQRVSSPRLGRRGSRLSGALPL
Mm_Pax1             ----------------------------------------------------------------------------------MKFTLGLGSRAWRVSWERAAAAAAGPGAGGALGSG-SLR----VSSRRGPRLARALPL
Hs_PAX9             --------------------------------------------------------------------------------------------------------------------------------------------
Mm_Pax9             --------------------------------------------------------------------------------------------------------------------------------------------
Eb_Pax1/9           --------------------------------------------------------------------------------------------------------------------------------------------
Sk_Pax1             --------------------------------------------------------------------------------------------------------------------------------------------
Cg_EKC39574         --------------------------------------------------------------------------------------------------------------------------------------------
Dm_Poxm             --------------------------------------------------------------------------------------------------------------------------------------------
Dm_Sv               -----MLIMDIQTSTHHIHGLGTHELQHRILHPHILHSTQEETLNTSTGQLEHDSQHLQQHHLTHHQQQDVSPTLHNLQNTRTGDSLSTTINTNQNQHGHQHLSGSNMYTSSQMEDKSKANKYDEYSSRTLSNISDANTT
Sk_Pax2             --------------------------------------------------------------------------------------------------------------------------------------------
Cg_EKC36239         -----------------------------------------------------------------------------------------------------------------------------------------MRL
Hs_PAX2             --------------------------------------------------------------------------------------------------------------------------------------------
Mm_Pax2             --------------------------------------------------------------------------------------------------------------------------------------------
Hs_PAX5             --------------------------------------------------------------------------------------------------------------------------------------------
Mm_Pax5             --------------------------------------------------------------------------------------------------------------------------------------------
Hs_PAX8             --------------------------------------------------------------------------------------------------------------------------------------------
Mm_Pax8             --------------------------------------------------------------------------------------------------------------------------------------------
Bf_Pax2/5/8         --------------------------------------------------------------------------------------------------------------------------------------------
Hs_PAX3             --------------------------------------------------------------------------------------------------------------------------------------------
Mm_Pax3             --------------------------------------------------------------------------------------------------------------------------------------------
Hs_PAX7             --------------------------------------------------------------------------------------------------------------------------------------------
Mm_Pax7             --------------------------------------------------------------------------------------------------------------------------------------------
Cg_EKC41820         ----------------------------------------------------------------------------------------------------------------------------------------MFGF
Sk_LOC100371646     --------------------------------------------------------------------------------------------------------------------------------------------
Dm_Gsb              --------------------------------------------------------------------------------------------------------------------------------------------
Dm_Gsbn             --------------------------------------------------------------------------------------------------------------------------------------------
Dm_Prd              --------------------------------------------------------------------------------------------------------------------------------------------
Hs_PAX4             --------------------------------------------------------------------------------------------------------------------------------------------
Mm_Pax4             --------------------------------------------------------------------------------------------------------------------------------------------
Hs_PAX6             --------------------------------------------------------------------------------------------------------------------------------------------
Mm_Pax6             --------------------------------------------------------------------------------------------------------------------------------------------
Bf_Pax6             --------------------------------------------------------------------------------------------------------------------------------------------
Sk_Pax6             --------------------------------------------------------------------------------------------------------------------------------------------
Dr_Pax10            --------------------------------------------------------------------------------------------------------------------------------------------
Dm_Toy              --------------------------------------------------------------------------------------------------------------------------------------------
Od_Pax6             --------------------------------------------------------------------------------------------------------------------------------------------
Dm_Ey               ----------------------------------------------------------------------------------------------------------------------------MRNLPCLGTAGGSGLG
Dpse_Ey             --------------------------------------------------------------------------------------MFTLQPTPTAISTVVPPWSAGTLIERLPSLEEMAHKDNVIAMRNLPCLSTASGS
Cg_EKC31583         --------------------------------------------------------------------------------------------------------------------------------------------
Dm_Eyg              ----------------------------------------------------MLIEPTLNRDLGSLTVANSGLSAAHWKM---EPHPQRITSLAPHASSVAAASVAAAAAAAAA----AAAAAAGAGSGAVAGGGGAT--
Md_Eyg              ----------------------------------------------------MLIESGINRDFGSIAPGSFN-IPAHWKM---DP--PRIPALNTPA----AANVAGAM-------------------------------
Dm_Toe              --------------------------------------------------MATSTSPGPTRTTAAIRVSLPGAAP-----------AIPATA-------TAGSQSPGAIMPMAAVPLPSHLQLLGSSAGGVGQPAAITPV
Am_Eyg              ----------------------MMIGGGVATPPGTAKSVAAID--SEMVQVDGIGSGSVSGSASGSGSPATGATTTRIAN-----SNNVGGNNNNNNNNGDVANNNNNNNEGHAVVNCGSGPGAPLTDKFCCHDDDAPVS
Cbir_X777_10953     ----------------------MMIGGGVATPPGTAKSAAAID--GEMVQVDGIGSGSVSGSASGSGSPATGATTTRLTNN--TNNGNNSNSNNNNNNNNNNGDVSNNNNDGHGVANCAGTTAASAGDKFCCQDDDVPAS
Nvit_Eyg            -----------------------MIGGGVATPPGQAKSAAAACELPQNHQESAAAKATTMLLEDRIGSASVSSAGASAAEPTSPLRTNSREDSNNNNNNNSNNNNNSGNHSSGNSSNDEETTTAQTTRRKCNSSGGSREE
Apis_LOC100165522   ------------------------------------------------------------------------------------------------------------------------------MANSTSVTKCTDFS
Sk_Eygone           --------------------------------------------------------------------------------------------------------------------------------------------
Spur_LOC574563      --------------------------------------------------------------------------------------------------------------------------------------------
Cg_EKC38178         --------------------------------------------------------------------------------------------------------------------------------------------
Ce_EYG-1            --------------------------------------------------------------------------------------------------------------------------------------------
Crem_EYG-1          --------------------------------------------------------------------------------------------------------------------------------------------
```

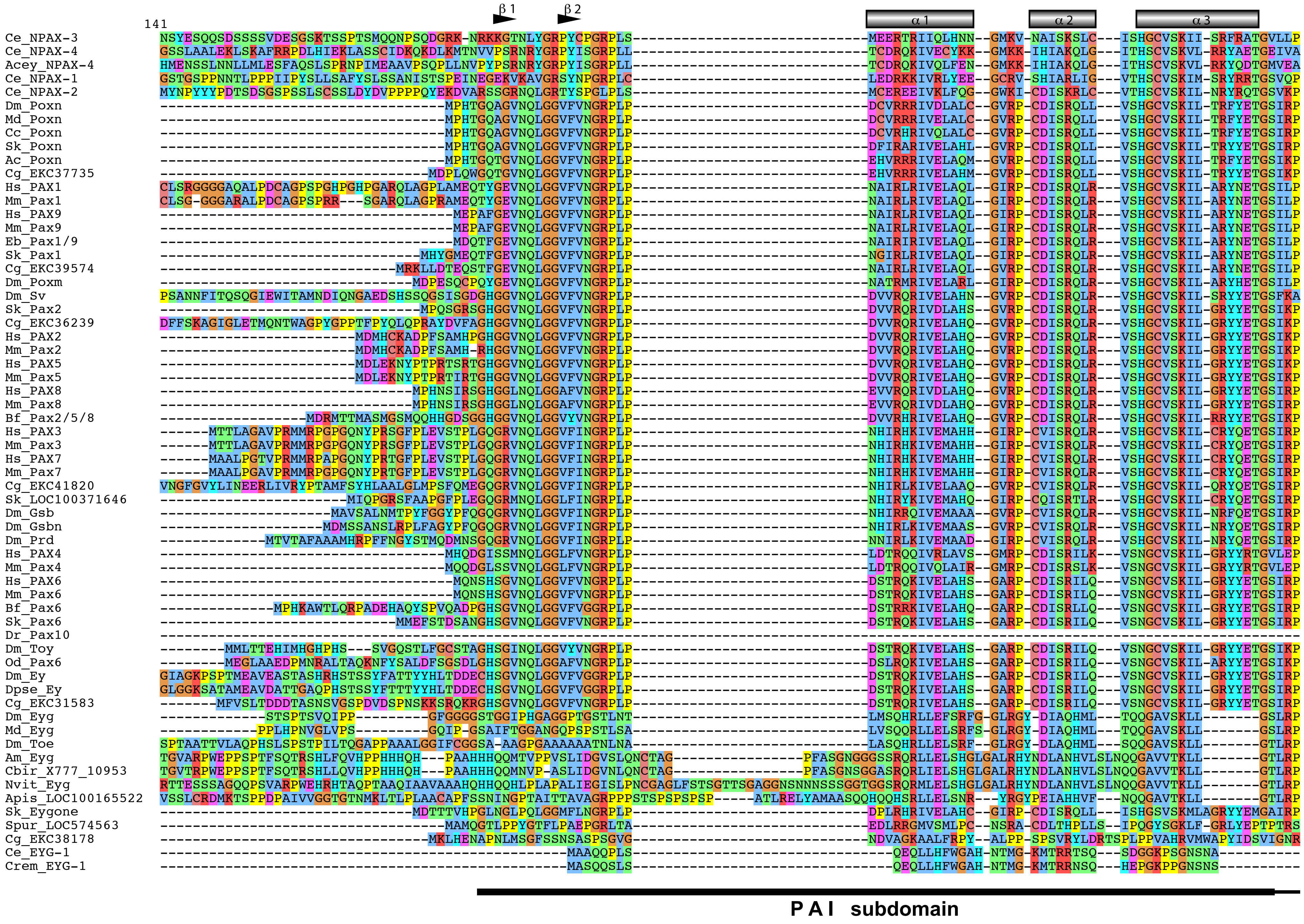

141
β 1
β 2
α 1
α 2
α 3
Ce_NPAX-3
Ce_NPAX-4
Acey_NPAX-4
Ce_NPAX-1
Ce_NPAX-2
Dm_Poxn
Md_Poxn
Cc_Poxn
Sk_Poxn
Ac_Poxn
Cg_EKC37735
Hs_PAX1
Mm_Pax1
Hs_PAX9
Mm_Pax9
Eb_Pax1/9
Sk_Pax1
Cg_EKC39574
Dm_Poxm
Dm_Sv
Sk_Pax2
Cg_EKC36239
Hs_PAX2
Mm_Pax2
Hs_PAX5
Mm_Pax5
Hs_PAX8
Mm_Pax8
Bf_Pax2/5/8
Hs_PAX3
Mm_Pax3
Hs_PAX7
Mm_Pax7
Cg_EKC41820
Sk_LOC100371646
Dm_Gsb
Dm_Gsbn
Dm_Prd
Hs_PAX4
Mm_Pax4
Hs_PAX6
Mm_Pax6
Bf_Pax6
Sk_Pax6
Dr_Pax10
Dm_Toy
Od_Pax6
Dm_Ey
Dpse_Ey
Cg_EKC31583
Dm_Eyg
Md_Eyg
Dm_Toe
Am_Eyg
Cbir_X777_10953
Nvit_Eyg
Apis_LOC100165522
Sk_Eygone
Spur_LOC574563
Cg_EKC38178
Ce_EYG-1
Crem_EYG-1
PAI subdomain

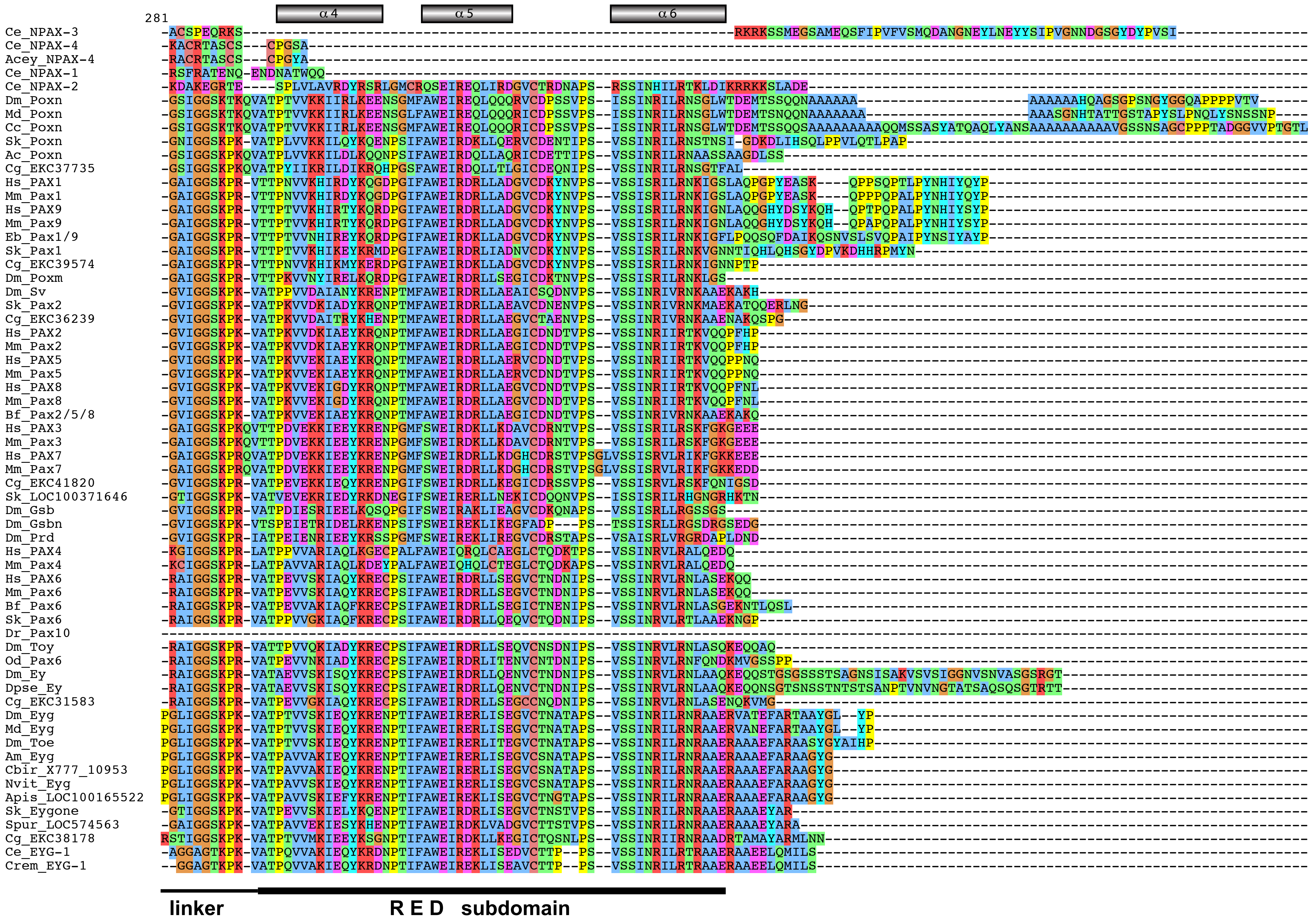
α4
α5
α6
281
Ce_NPAX-3 -ACSPEQRKS-RKRKSSMEGSAMEQSFIPVFVSMQDANGNEYLNEYYSIPVGNNDGSGYDYPVSI
Ce_NPAX-4 -KACRTASCS---CPGSA
Acey_NPAX-4 -RACRTASCS---CPGYA
Ce_NPAX-1 -RSFRATENQ-ENDNATWQQ
Ce_NPAX-2 -KDAKEGRTE----SPLVLAVRDYRSRLGMCRQSEIREQLIRDGVCTRDNAPS--RSSINHILRTKLDIKRRKKSLADE
Dm_Poxn -GSIGGSKTKQVATPTVVKKIIRLKEENSGMFAWEIREQLQQQRVCDPSSVPS--ISSINRILRNSGLWTDEMTSSQQNAAAAAA-AAAAAAHQAGSGPSNGYGGQAPPPPVTV
Md_Poxn -GSIGGSKTKQVATPTVVKKIIRLKEENSGLFAWEIREQLQQQRICDPSSVPS--ISSINRILRNSGLWTDEMTSNQQNAAAAAAA-AAASGNHTATTGSTAPYSLPNQLYSNSSNP
Cc_Poxn -GSIGGSKTKQVATPTVVKKIIRLKEENSGMFAWEIREQLQQQRICDPSSVPS--ISSINRILRNSGLWTDEMTSSQQSAAAAAAAAAQQMSSASYATQAQLYANSAAAAAAAAAAVGSSNSAGCPPPTADGGVVPTGTL
Sk_Poxn -GNIGGSKPK-VATPLVVKKILQYKQENPSIFAWEIRDKLLQERVCDENTIPS--VSSINRILRNSTNSI-GDKDLIHSQLPPVLQTLPAP
Ac_Poxn -GSIGGSKPKQVATPLVVKKILDLKQQNPSIFAWEIRDQLLAQRICDETTIPS--VSSINRILRNAASSAAGDLSS
Cg_EKC37735 -GSIGGSKPKQVATPYIIKRILDIKRQHPGSFAWEIRDQLLTLGICDEQNIPS--VSSINRILRNSGTFAL
Hs_PAX1 -GAIGGSKPR-VTTPNVVKHIRDYKQGDPGIFAWEIRDRLLADGVCDKYNVPS--VSSISRILRNKIGSLAQPGPYEASK----QPPSQPTLPYNHIYQYP
Mm_Pax1 -GAIGGSKPR-VTTPNVVKHIRDYKQGDPGIFAWEIRDRLLADGVCDKYNVPS--VSSISRILRNKIGSLAQPGPYEASK----QPPPQPALPYNHIYQYP
Hs_PAX9 -GAIGGSKPR-VTTPTVVKHIRTYKQRDPGIFAWEIRDRLLADGVCDKYNVPS--VSSISRILRNKIGNLAQQGHYDSYKQH--QPTPQPALPYNHIYSYP
Mm_Pax9 -GAIGGSKPR-VTTPTVVKHIRTYKQRDPGIFAWEIRDRLLADGVCDKYNVPS--VSSISRILRNKIGNLAQQGHYDSYKQH--QPAPQPALPYNHIYSYP
Eb_Pax1/9 -GAIGGSKPR-VTTPTVVNHIREYKQRDPGIFAWEIRDKLLADGVCDKYNVPS--VSSISRILRNKIGFLPQQSQFDAIKQSNVSLSVQPAIPYNSIYAYP
Sk_Pax1 -GAIGGSKPR-VTTPTVVKHIKEYKRMDPGIFAWEIRDRLIADNVCDKYNVPS--VSSISRILRNKVGNNTIQHLQHSGYDPVKDHHRPMYN
Cg_EKC39574 -GAIGGSKPR-VTTPNVVKHIKMYKERDPGIFAWEIRDKLLADGVCDKYNVPS--VSSISRILRNKIGNNPTP
Dm_Poxm -GAIGGSKPR-VTTPKVVNYIRELKQRDPGIFAWEIRDRLLSEGICDKTNVPS--VSSISRILRNKLGS
Dm_Sv -GVIGGSKPK-VATPPVVDAIANYKRENPTMFAWEIRDRLLAEAICSQDNVPS--VSSINRIVRNKAAEKAKH
Sk_Pax2 -GVIGGSKPK-VATPKVVDKIADYKRQNPTMFAWEIRDRLLAEAVCDNENVPS--VSSINRIVRNKMAEKATQQERLNG
Cg_EKC36239 -GVIGGSKPK-VATPKVVDAITRYKHENPTMFAWEIRDRLLAEGVCTAENVPS--VSSINRIVRNKAAENAKQSPG
Hs_PAX2 -GVIGGSKPK-VATPKVVDKIAEYKRQNPTMFAWEIRDRLLAEGICDNDTVPS--VSSINRIIRTKVQQPFHP
Mm_Pax2 -GVIGGSKPK-VATPKVVDKIAEYKRQNPTMFAWEIRDRLLAEGICDNDTVPS--VSSINRIIRTKVQQPFHP
Hs_PAX5 -GVIGGSKPK-VATPKVVEKIAEYKRQNPTMFAWEIRDRLLAERVCDNDTVPS--VSSINRIIRTKVQQPPNQ
Mm_Pax5 -GVIGGSKPK-VATPKVVEKIAEYKRQNPTMFAWEIRDRLLAERVCDNDTVPS--VSSINRIIRTKVQQPPNQ
Hs_PAX8 -GVIGGSKPK-VATPKVVEKIGDYKRQNPTMFAWEIRDRLLAEGVCDNDTVPS--VSSINRIIRTKVQQPFNL
Mm_Pax8 -GVIGGSKPK-VATPKVVEKIGDYKRQNPTMFAWEIRDRLLAEGVCDNDTVPS--VSSINRIIRTKVQQPFNL
Bf_Pax2/5/8 -GVIGGSKPK-VATPKVVEKIAEYKRQNPTMFAWEIRDRLLAEGICDNDTVPS--VSSINRIVRNKAAEKAKQ
Hs_PAX3 -GAIGGSKPKQVTTPDVEKKIEEYKRENPGMFSWEIRDKLLKDAVCDRNTVPS--VSSISRILRSKFGKGEEE
Mm_Pax3 -GAIGGSKPKQVTTPDVEKKIEEYKRENPGMFSWEIRDKLLKDAVCDRNTVPS--VSSISRILRSKFGKGEEE
Hs_PAX7 -GAIGGSKPRQVATPDVEKKIEEYKRENPGMFSWEIRDRLLKDGHCDRSTVPSGLVSSISRVLRIKFGKKEEE
Mm_Pax7 -GAIGGSKPRQVATPDVEKKIEEYKRENPGMFSWEIRDRLLKDGHCDRSTVPSGLVSSISRVLRIKFGKKEDD
Cg_EKC41820 -GVIGGSKPR-VATPEVEKKIEQYKRENPGIFSWEIRDRLLKEGICDRSSVPS--VSSISRVLRSKFQNIGSD
Sk_LOC100371646 -GTIGGSKPK-VATVEVEKRIEDYRKDNEGIFSWEIRERLLNEKICDQQNVPS--ISSISRILRHGNGRHKTN
Dm_Gsb -GVIGGSKPR-VATPDIESRIEELKQSQPGIFSWEIRAKLIEAGVCDKQNAPS--VSSISRLLRGSSGS
Dm_Gsbn -GVIGGSKPK-VTSPEIETRIDELRKENPSIFSWEIREKLIKEGFADP---PS--TSSISRLLRGSDRGSEDG
Dm_Prd -GVIGGSKPR-IATPEIENRIEEYKRSSPGMFSWEIREKLIREGVCDRSTAPS--VSAISRLVRGRDAPLDND
Hs_PAX4 -KGIGGSKPR-LATPPVVARIAQLKGECPALFAWEIQRQLCAEGLCTQDKTPS--VSSINRVLRALQEDQ
Mm_Pax4 -KCIGGSKPR-LATPAVVARIAQLKDEYPALFAWEIQHQLCTEGLCTQDKAPS--VSSINRVLRALQEDQ
Hs_PAX6 -RAIGGSKPR-VATPEVVSKIAQYKRECPSIFAWEIRDRLLSEGVCTNDNIPS--VSSINRVLRNLASEKQQ
Mm_Pax6 -RAIGGSKPR-VATPEVVSKIAQYKRECPSIFAWEIRDRLLSEGVCTNDNIPS--VSSINRVLRNLASEKQQ
Bf_Pax6 -RAIGGSKPR-VATPEVVAKIAQFKRECPSIFAWEIRDRLLSEGICTNENIPS--VSSINRVLRNLASGEKNTLQSL
Sk_Pax6 -RAIGGSKPR-VATPPVVGKIAQFKRECPSIFAWEIRDRLLQEQVCTQDNIPS--VSSINRVLRTLAAEKNGP
Dr_Pax10
Dm_Toy -RAIGGSKPR-VATTPVVQKIADYKRECPSIFAWEIRDRLLSEQVCNSDNIPS--VSSINRVLRNLASQKEQQAQ
Od_Pax6 -RAIGGSKPR-VATPEVVNKIADYKRECPSIFAWEIRDRLITENVCNTDNIPS--VSSINRVLRNFQNDKMVGSSPP
Dm_Ey -RAIGGSKPR-VATAEVVSKISQYKRECPSIFAWEIRDRLLQENVCTNDNIPS--VSSINRVLRNLAAQKEQQSTGSGSSSTSAGNSISAKVSVSIGGNVSNVASGSRGT
Dpse_Ey -RAIGGSKPR-VATAEVVSKISQYKRECPSIFAWEIRDRLLQENVCTNDNIPS--VSSINRVLRNLAAQKEQQNSGTSNSSTNTSTSANPTVNVNGTATSAQSQSGTRTT
Cg_EKC31583 -RAIGGSKPR-VATPEVVGKIAQYKRECPSIFAWEIRDRLLSEGCCNQDNIPS--VSSINRVLRNLASENQKVMG
Dm_Eyg PGLIGGSKPK-VATPTVVSKIEQYKRENPTIFAWEIRERLISEGVCTNATAPS--VSSINRILRNRAAERVATEFARTAAYGL--YP
Md_Eyg PGLIGGSKPK-VATPTVVSKIEQYKRENPTIFAWEIRERLISEGVCTNATAPS--VSSINRILRNRAAERVANEFARTAAYGL--YP
Dm_Toe PGLIGGSKPK-VATPTVVSKIEQYKRENPTIFAWEIRERLITEGVCTNATAPS--VSSINRILRNRAAERAAAEFARAASYGYAIHP
Am_Eyg PGLIGGSKPK-VATPAVVAKIEQYKRENPTIFAWEIRERLISEGVCSNATAPS--VSSINRILRNRAAERAAAEFARAAGYG
Cbir_X777_10953 PGLIGGSKPK-VATPAVVAKIEQYKRENPTIFAWEIRERLISEGVCSNATAPS--VSSINRILRNRAAERAAAEFARAAGYG
Nvit_Eyg PGLIGGSKPK-VATPAVVSKIEQYKRENPTIFAWEIRERLISEGVCSNATAPS--VSSINRILRNRAAERAAAEFARAAGYG
Apis_LOC100165522 PGLIGGSKPK-VATPAVVSKIEFYKRENPTIFAWEIREKLISEGVCTNGTAPS--VSSINRILRNRAAERAAAEFARAAGYG
Sk_Eygone -GTIGGSKPK-VATPEVVSKIELYKQENPTIFAWEIRDRLISEGVCTNSTVPS--VSSINRILRNRAAERAAAEYAR
Spur_LOC574563 -GAIGGSKPK-VATPAVVEKIESYKHENPTIFAWEIRDKLVADGVCTTSTVPS--VSSINRILRNRAAERAAAEYARA
Cg_EKC38178 RSTIGGSKPK-VATPTVVMKIEEYKSGNPTIFAWEIRDKLLKEGICTQSNLPS--VSSINRIIRNRAADRTAMAYARMLNN
Ce_EYG-1 -AGGAGTKPK-VATPQVVAKIEQYKRDNPTIFAWEIREKLISEDVCTTP--PS--VSSINRILRTRAAERAAEELQMILS
Crem_EYG-1 --GGAGTKPK-VATPQVVAKIEQYKRDNPTIFAWEIREKLISEAVCTTP--PS--VSSINRILRTRAAERAAEELQMILS
linker
R E D subdomain

```
                      421
Ce_NPAX-3             --------------------------------------------------------------------------------------------------------------------------------------------
Ce_NPAX-4             --------------------------------------------------------------------------------------------------------------------------------------------
Acey_NPAX-4           --------------------------------------------------------------------------------------------------------------------------------------------
Ce_NPAX-1             --------------------------------------------------------------------------------------------------------------------------------------------
Ce_NPAX-2             --------------------------------------------------------------------------------------------------------------------------------------------
Dm_Poxn               --------------------------------APPTPAATPSIARYAKPPALMMNSAG--------------------------EMPIKPAPKMPPSMGH--------GHSHGL------------NPNVSGLDLSY-SA
Md_Poxn               ------------------------------LPPATMALASSYRYADINMPTASNTPAHAAAS----------------------EHPIKPSPK-HPMVGL--------GATPST-----------STPQLTPQDLSYSAA
Cc_Poxn               LHRASLTPLSTAHHAHTSTHLTAANAHPHNAAPHALTLPAQFRYSSAAAAAAAAAAMSANESNRGNNATTTQDARLLASPGSGVEMPIKPAPK-HPVAAHMTALTTAGGSTSALAQLSATVSSAVSTGHLSPQDLSY-AA
Sk_Poxn               --------------------------------------------------------------------------------------------------------------------------------------------
Ac_Poxn               --------------------------------------------------------------------------------------------------------------------------------------------
Cg_EKC37735           --------------------------------------------------------------------------------------------------------------------------------------------
Hs_PAX1               --------------------------------------------------------------------------------------------------------------------------------------------
Mm_Pax1               --------------------------------------------------------------------------------------------------------------------------------------------
Hs_PAX9               --------------------------------------------------------------------------------------------------------------------------------------------
Mm_Pax9               --------------------------------------------------------------------------------------------------------------------------------------------
Eb_Pax1/9             --------------------------------------------------------------------------------------------------------------------------------------------
Sk_Pax1               --------------------------------------------------------------------------------------------------------------------------------------------
Cg_EKC39574           --------------------------------------------------------------------------------------------------------------------------------------------
Dm_Poxm               --------------------------------------------------------------------------------------------------------------------------LGHQHTPGTVMGSGSSSG
Dm_Sv                 --------------------------------------------------------------------------------------------------------------------------------------------
Sk_Pax2               --------------------------------------------------------------------------------------------------------------------------------------------
Cg_EKC36239           --------------------------------------------------------------------------------------------------------------------------------------------
Hs_PAX2               --------------------------------------------------------------------------------------------------------------------------------------------
Mm_Pax2               --------------------------------------------------------------------------------------------------------------------------------------------
Hs_PAX5               --------------------------------------------------------------------------------------------------------------------------------------------
Mm_Pax5               --------------------------------------------------------------------------------------------------------------------------------------------
Hs_PAX8               --------------------------------------------------------------------------------------------------------------------------------------------
Mm_Pax8               --------------------------------------------------------------------------------------------------------------------------------------------
Bf_Pax2/5/8           --------------------------------------------------------------------------------------------------------------------------------------------
Hs_PAX3               --------------------------------------------------------------------------------------------------------------------------------------------
Mm_Pax3               --------------------------------------------------------------------------------------------------------------------------------------------
Hs_PAX7               --------------------------------------------------------------------------------------------------------------------------------------------
Mm_Pax7               --------------------------------------------------------------------------------------------------------------------------------------------
Cg_EKC41820           --------------------------------------------------------------------------------------------------------------------------------------------
Sk_LOC100371646       --------------------------------------------------------------------------------------------------------------------------------------------
Dm_Gsb                --------------------------------------------------------------------------------------------------------------------------------------------
Dm_Gsbn               --------------------------------------------------------------------------------------------------------------------------------------------
Dm_Prd                --------------------------------------------------------------------------------------------------------------------------------------------
Hs_PAX4               --------------------------------------------------------------------------------------------------------------------------------------------
Mm_Pax4               --------------------------------------------------------------------------------------------------------------------------------------------
Hs_PAX6               --------------------------------------------------------------------------------------------------------------------------------------------
Mm_Pax6               --------------------------------------------------------------------------------------------------------------------------------------------
Bf_Pax6               --------------------------------------------------------------------------------------------------------------------------------------------
Sk_Pax6               --------------------------------------------------------------------------------------------------------------------------------------------
Dr_Pax10              --------------------------------------------------------------------------------------------------------------------------------------------
Dm_Toy                --------------------------------------------------------------------------------------------------------------------------------------------
Od_Pax6               --------------------------------------------------------------------------------------------------------------------------------------------
Dm_Ey                 -----------------------------------------------------------------------------------------------------------------------------------LSSSTDLMQ
Dpse_Ey               -----------------------------------------------------------------------------------------------------------------------------------LSSGGDLMQ
Cg_EKC31583           --------------------------------------------------------------------------------------------------------------------------------------------
Dm_Eyg                --------------------------------------------------------------------------------------------------------------------------------------------
Md_Eyg                --------------------------------------------------------------------------------------------------------------------------------------------
Dm_Toe                --------------------------------------------------------------------------------------------------------------------------------------------
Am_Eyg                --------------------------------------------------------------------------------------------------------------------------------------------
Cbir_X777_10953       --------------------------------------------------------------------------------------------------------------------------------------------
Nvit_Eyg              --------------------------------------------------------------------------------------------------------------------------------------------
Apis_LOC100165522     --------------------------------------------------------------------------------------------------------------------------------------------
Sk_Eygone             --------------------------------------------------------------------------------------------------------------------------------------------
Spur_LOC574563        ----------------------------------------------------------------------------------------------------------------------------------------TEQA
Cg_EKC38178           --------------------------------------------------------------------------------------------------------------------------------------------
Ce_EYG-1              --------------------------------------------------------------------------------------------------------------------------------------------
Crem_EYG-1            --------------------------------------------------------------------------------------------------------------------------------------------
```

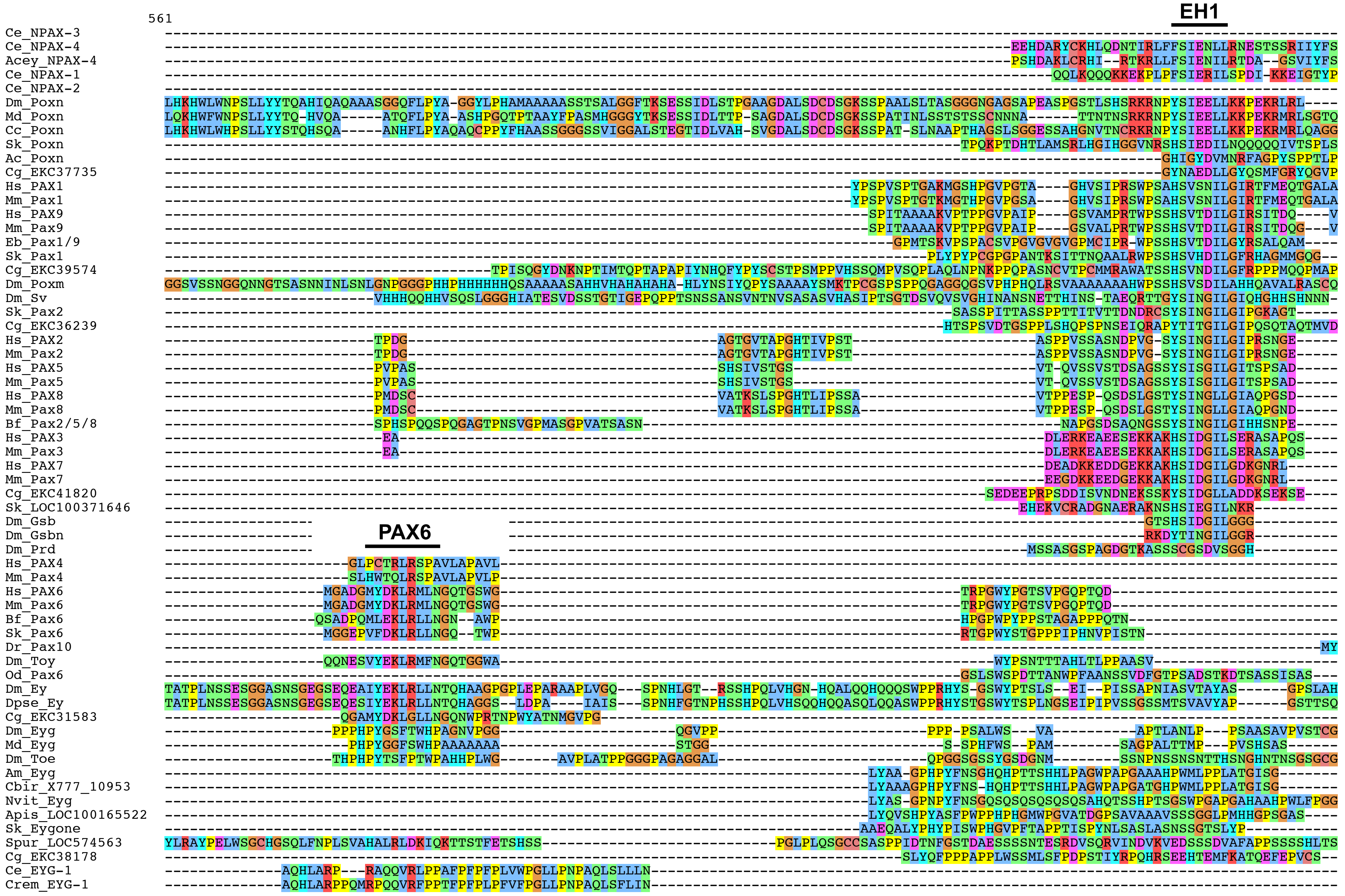
561
EH1
PAX6
Ce_NPAX-3
Ce_NPAX-4
Acey_NPAX-4
Ce_NPAX-1
Ce_NPAX-2
Dm_Poxn
Md_Poxn
Cc_Poxn
Sk_Poxn
Ac_Poxn
Cg_EKC37735
Hs_PAX1
Mm_Pax1
Hs_PAX9
Mm_Pax9
Eb_Pax1/9
Sk_Pax1
Cg_EKC39574
Dm_Poxm
Dm_Sv
Sk_Pax2
Cg_EKC36239
Hs_PAX2
Mm_Pax2
Hs_PAX5
Mm_Pax5
Hs_PAX8
Mm_Pax8
Bf_Pax2/5/8
Hs_PAX3
Mm_Pax3
Hs_PAX7
Mm_Pax7
Cg_EKC41820
Sk_LOC100371646
Dm_Gsb
Dm_Gsbn
Dm_Prd
Hs_PAX4
Mm_Pax4
Hs_PAX6
Mm_Pax6
Bf_Pax6
Sk_Pax6
Dr_Pax10
Dm_Toy
Od_Pax6
Dm_Ey
Dpse_Ey
Cg_EKC31583
Dm_Eyg
Md_Eyg
Dm_Toe
Am_Eyg
Cbir_X777_10953
Nvit_Eyg
Apis_LOC100165522
Sk_Eygone
Spur_LOC574563
Cg_EKC38178
Ce_EYG-1
Crem_EYG-1

```
                    701
Ce_NPAX-3           --------------------------------------------------------------------------------------------------------------------------------------------
Ce_NPAX-4           KPPSYSTLEI----------------------------------------------------------------------------------------------------------------------------------
Acey_NPAX-4         KAKDES--------------------------------------------------------------------------------------------------------------------------------------
Ce_NPAX-1           RVISQ---------------------------------------------------------------------------------------------------------------------------------------
Ce_NPAX-2           --------------------------------------------------------------------------------------------------------------------------------------------
Dm_Poxn             --------------------------------DSNRLECLESSSCESSQDSPVAPPLETPEDEDPAEAEEEQEEEDSVEVVN----------------------------------------------------------
Md_Poxn             SHLHSSASGALRLSPKKDDCNISLCSASSSHSNSSHISCVGDDSCD---EATTRSPLHNPANGN----NDAEENCETVEVVN----------------------------------------------------------
Cc_Poxn             SARQDNESSA----------------SSISSSGSSRSSC-CDDSGD----ETRSQPAETLAADD--------CNDNVEVVN----------------------------------------------------------
Sk_Poxn             SSSFINTPLPTSTTMAMSPAAMQTSCASLTGDDKRLNGGLKRKRSVDEPHAWTTVMKGDNDKLAQSVNMNGATTMMSHPCTVTSPIPGTISGNVATSP-----AMKHTNDVIAPPAKKMCEVPAMTKLSPSMTTNLTTSD
Ac_Poxn             LFPLPAYPQGWYSHLPMPGLSYPRLVTPLLPEVEPESLTPRLVRCDGDSDFLSHHERKRKASASSSIENKVCKYEGDSKLAAMSPSQRLTPASTQNSGKDVDSDSDVKVADETRNINPFKMSFTNPDGIETSRDRIQCVS
Cg_EKC37735         SIAPPPCPEANGFSLAVPGLSYPKLVEQIN-DDRKDSEQSDDCDKETEGKGSSKHNERQKSNKDDDDEFKIEERFPSLAGKIRSVNSANIRNRTSPQHCAND-IYDNATTENVDGNKNPVEKTDDSPNETFLKHRSRHDT
Hs_PAX1             GSEGTA------YSPKMEDWAGVNRTAFPAT--PAVNGLEK-PALEADIKYTQSASTLSAVGGFLPACA---YPASNQHG---VYSAPGGGYLAPGPP-----WPPAQGPPLAPPGAGVAVHGGELAAAMTFKHPS--RE
Mm_Pax1             GSEGAA------YSPKMEDWAGVNRAAFPTS--PAVNGLEK-PALEADIKYTQSASSLSAVGGFLPACA---YPASNQHG---VYSAPAAGYLSPGPP-----WPPAQAPPLTPHGAGVAVHGGELAAAMTFKH----RE
Hs_PAX9             SDSSPY------HSPKVEEWSSLGRNNFPAAAPHAVNGLEK-GALEQEAKYGQAPNGLPAVGSFVSASSMAPYPTPAQVSPYMTYSAAPSGYV-AGHG-----WQHAGGTSLSP-------HNCDIPASLAFKGMQAARE
Mm_Pax9             SDSSPY------HSPKVEEWSSLGRNNFPAAAPHAVNGLEK-GALEQEAKYGQAPNGLPAVSSFVSASSMAPYPTPAQVSPYMTYSAAPSGYV-AGHG-----WQHAGSTPLSP-------HNCDIPASLAFKGMQAARE
Eb_Pax1/9           -GESAA------YPTKMEDWTSMSRAAFAGG-CAASALPEKATPLEPQLKYTQAH-----VSAYVPYGA----SGSLMAAEAPAWSAQG-----VGIG-----RIPTHPGELTAG------LTGLRFRTSSAREVEDTRE
Sk_Pax1             ------------------------------------------PDHQGPFAMYPDSTGHSHAASSLADYSRPPHHATTGYPGPADHQRSSMHCESVKAE------KSHLEGDTGKMSISPTDPSLPNVGSFVPSTLTISPY
Cg_EKC39574         HHQPLPQDNMGNLGQNYNMNYYAMKPTSVAPMYLQS---------------------------------------------------------------------------------------------------------
Dm_Poxm             VGVGVGGMGGMGSTVSPLPMTPSPVAGTAGGQPLLDCEGGAGQQSPYNYYMYFQNGGMHHHHHHGGMMAAGATGL-----------------------------------------------------------------
Dm_Sv               ------NNSSVNNNNNTESSCKRKRIEAHDENHDTNIHS-------------------------DNDDGKRQRM--STYSGDQLYTNIWSGKWCIK--DDHKLLAELGNLTASTGNCPATYYEASNGFSTTPISGSGATA
Sk_Pax2             --------------------EKRKRDSQSDHHESQAN----------------------GVISSPAMENDSRKRSRPTYTSEQIEALEKAFDRSHFP-ESASTGNYHDDMIVKNAYGRDDVI-QKNEQSANQGYQTLSPL
Cg_EKC36239         -----------------PNGNKRKREDQVPNGHEAEVKIEPNTGDMNIWYQAQPRPTKQARPENGTTDAQGMPHTLIINNAPMYPTGVPTNLPPNYN-AHFVTTTTTISGDVIKPQVGVEYTIASPAIGTVANSESQSTT
Hs_PAX2             ---------------------KRKRDEVEVYTDPAHIRGGGGLHLVWTLRDVSEGSVPNGDSQSGVDSLRKHLRADT-FTQQQLEALDRVFERPSYP-DVFQAS-EHIKSEQGNEYSLPALT-PGLDEVKSSLSASTNPE
Mm_Pax2             ---------------------KRKREEVEVYTDPAHIRGGGGLHLVWTLRDVSEGSVPNGDSQSGVDSLRKHLRADT-FTQQQLEALDRVFERPSYP-DVFQAS-EHIKSEQGNEYSLPALT-PGLDEVKSSLSASANPE
Hs_PAX5             -------------------TNKRKRDE-----------------------GIQESPVPNGHSLPGRDFLRKQMRGDL-FTQQQLEVLDRVFERQHYS-DIFTTT-EPIKPEQTTEYSAMASLAGGLDDMKANLASPTPAD
Mm_Pax5             -------------------TNKRKRDE-----------------------GIQESPVPNGHSLPGRDFLRKQMRGDL-FTQQQLEVLDRVFERQHYS-DIFTTT-EPIKPEQTTEYSAMASLAGGLDDMKANLTSPTPAD
Hs_PAX8             ---------------------KRKMDD-----------------------SDQDSCRLSIDSQSSSSGPRKHLRTDA-FSQHHLEPLECPFERQHYP-EAYASP-SHTKGEQGL-YPLPLLN-STLDDGKATLTPSNTP-
Mm_Pax8             --------------------NKRKMDD-----------------------SDQDSCRLSIDSQSSSSGPRKHLRTDT-FSQHHLEALECPFERQHYP-EAYASP-SHTKGEQGL-YPLPLLN-SALDDGKATLTSSNTP-
Bf_Pax2/5/8         ---------------------KVKREG--------------------------DRETGPAMENGMIVNGDPEQKRST-FTPDQLEALEQAFNRGHYPTDPFNRDNMSNKVDLSQTRVQDVK-------------------
Hs_PAX3             ------------------------------------------------------DEGSDIDSEPDLPLKRKQRRSRTTFTAEQLEELERAFERTHYP-DIYTREELAQRAKLTEARVQVWFSNRRARWRKQAGANQLMA-
Mm_Pax3             ------------------------------------------------------DEGSDIDSEPDLPLKRKQRRSRTTFTAEQLEELERAFERTHYP-DIYTREELAQRAKLTEARVQVWFSNRRARWRKQAGANQLMA-
Hs_PAX7             ------------------------------------------------------DEGSDVESEPDLPLKRKQRRSRTTFTAEQLEELEKAFERTHYP-DIYTREELAQRTKLTEARVQVWFSNRRARWRKQAGANQLAA-
Mm_Pax7             ------------------------------------------------------DEGSDVESEPDLPLKRKQRRSRTTFTAEQLEELEKAFERTHYP-DIYTREELAQRTKLTEARVQVWFSNRRARWRKQAGANQLAA-
Cg_EKC41820         ------------------------------------------------------DEESDCDSEPGLSVKRKQRRSRTTFTAEQLEELERAFERTHYP-DIYTREELAQRTKLTEARVQVWFSNRRARWRKQMGSNQISAL
Sk_LOC100371646     ----------------------------------------------------DEEPDYEPELQPDLPLKHKQRRCRTTFTCEQLEQLEKAFDRTHYP-DIYTREELAQRTGLTEARVQVWFSNRRARWRKQASFLPLNGY
Dm_Gsb              ------------------------------------------------AGSVGSEDESEDDAEPSVQLKRKQRRSRTTFSNDQIDALERIFARTQYP-DVYTREELAQSTGLTEARVQVWFSNRRARLRKQLNTQQVPS-
Dm_Gsbn             -----------------------------------------------------DSDISDTESEPGIPLKRKQRRSRTTFTAEQLEALERAFSRTQYP-DVYTREELAQTTALTEARIQVWFSNRRARLRKHSGGSNSGLS
Dm_Prd              ----------------------------------------------HNNGKPSDEDISDCESEPGIALKRKQRRCRTTFSASQLDELERAFERTQYP-DIYTREELAQRTNLTEARIQVWFSNRRARLRKQHTSVSGGAP
Hs_PAX4             -------------------------------------------------------TPHSGSETPRGTHPGTGHRNRTIFSPSQAEALEKEFQRGQYP-DSVARGKLATATSLPEDTVRVWFSNRRAKWRRQEKLKWEMQ-
Mm_Pax4             -------------------------------------------------------SPHSNCGAPRGPHPGTSHRNRTIFSPGQAEALEKEFQRGQYP-DSVARGKLAAATSLPEDTVRVWFSNRRAKWRRQEKLKWEAQ-
Hs_PAX6             -------GCQQQEGGGENTNS------------------------------ISSNGEDSDEAQMRLQLKRKLQRNRTSFTQEQIEALEKEFERTHYP-DVFARERLAAKIDLPEARIQVWFSNRRAKWRREEKLRNQRRQ
Mm_Pax6             -------GCQQQEGGGENTNS------------------------------ISSNGEDSDEAQMRLQLKRKLQRNRTSFTQEQIEALEKEFERTHYP-DVFARERLAAKIDLPEARIQVWFSNRRAKWRREEKLRNQRRQ
Bf_Pax6             -----------------------GNVT-TKKEGDGKLASQILTL-HGYQDQGDGSNDDSDEAQARLRLKRKLQRNRTSFTQEQIEALEKEFERTHYP-DVFARERLAAKIDLPEARIQVWFSNRRAKWRREEKLRNQRRS
Sk_Pax6             -------------GTTPTNTSSTEGLVCSKKDIDA--------GEISDHKDGTDASGDDDEAQARLRLKRKLQRNRTSFTQYQIETLEKEFERTHYP-DVFARERLAQKIDLPEARIQVWFSNRRAKWRREEKLRNQRRS
Dr_Pax10            DNLTMLNLQNSSNWSGPNNWYHPADTITPQHGTDGCVEDLNVQMAGAGEGVVQGERDEVDDSQLHLQLKRKLQRNRTSFTQEQIDALEKEFERTHYP-DVFARERLAAKIDLPEARIQVWFSNRRAKWRREEKLRNQRRS
Dm_Toy              VTSPANLS-GQADRDDVQKRELQFSVEVS--HTNSHDSTSDGN---------SEHNSSGDEDSQMRLRLKRKLQRNRTSFSNEQIDSLEKEFERTHYP-DVFARERLADKIGLPEARIQVWFSNRRAKWRREEKMRTQRRS
Od_Pax6             ---------------------------------------------------DEDRVKEDPDIQARLQLKRKLQRNRTSFTQQQIESLESEFERTHYP-DVFARERLATKIGLPEARIQVWFSNRRAKWRREEKMRNQRPA
Dm_Ey               SLSPPNDIESLASIGHQRNCPVATEDIHLKKELDGHQSDETGSGEGENSNGGASNIGNTEDDQARLILKRKLQRNRTSFTNDQIDSLEKEFERTHYP-DVFARERLAGKIGLPEARIQVWFSNRRAKWRREEKLRNQRRT
Dpse_Ey             PLSPPNLA-GGGNHNHLSNCPMTTDDILLKKELDGHQSDETGSGEGENSNGGASNIGNSEDDQARLILKRKLQRNRTSFTNEQIDSLEKEFERTHYP-DVFARERLAGKIGLPEARIQVWFSNRRAKWRREEKLRNQRRT
Cg_EKC31583         ----------------------LPPSAYTHQPTPTLGMEKKNNDGSSSESNQSEGQNNETDEQLRLRLKRKLQRNRTSFTAAQIEALEKEFERTHYP-DVFARERLSQKIDLPEARIQVWFSNRRAKWRREEKLRNQRRE
Dm_Eyg              SLSSAHLMAGGAGGT------PTNR--AISPGSGSHDTLES-A---DENRHID---SD----YLDDDDEPKFRRNRTTFSPEQLEELEKEFDKSHYP-CVSTRERLSSRTSLSEARVQVWFSNRRAKWRRHQRMNLLKRQ
Md_Eyg              ----------VINVN------STSR--AISPSSGSHDTLES-P---DDSRHID---SD----YLDDDDEPKFRRNRTTFTPEQLEELEKEFDKSHYP-CVSTRERLSSRTSLSEARVQVWFSNRRAKWRRHQRMNLLKRR
Dm_Toe              DSSA-------GSGR------LSLP--ALSPDSGSRDSRSPDA---DANRMID---IEGEDSESQDSDQPKFRRNRTTFSPEQLDELEKEFDKSHYP-CVNTREKLAARTALSEARVQVWFSNRRAKWRRHQRVNLIKQR
Am_Eyg              -------AASALLLPPSLSPGAAAAAAAASASAAGAPDHSLHA-DAIARGYL-QDGDGDEGSLDGSEQPKFRRNRTTFSPEQLEELEKEFERSHYP-CVSTRERLASKTSLSEARVQVWFSNRRAKWRRHQRMNLLKRS
Cbir_X777_10953     --------ASALLLPPSLSPGAAAAAAAAASASVAGSSEHALHAADAIARGYL-QDGDGDDGSLDGSEQPKFRRNRTTFSPEQLEELEKEFERSHYP-CVSTRERLASKTSLSEARVQVWFSNRRAKWRRHQRMNLLKRS
Nvit_Eyg            LPAGLAGPASALLLQPPLPSAAAAAAAAAAAAAS--PEHALHAVDAIARGYLHQEGDGEDGSLDGSEQPKFRRNRTTFSPEQLEELEKEFERSHYP-CVSTRERLASKTSLSEARVQVWFSNRRAKWRRHQRMNLLKRS
Apis_LOC100165522   ---------------NKNTPLQQMINAHQPCGVMSSRDLMPRLLGDGSGGVDGCKDDMESVGSGSGSEQPKFRRNRTTFSPDQLDELEKEFDKSHYP-CVSTRERLAAKTSLSEARVQVWFSNRRAKWRRHQRMNKSRRS
Sk_Eygone           --------------------------TSLLNIPPMTSLRGIPTSSVPTFTTTSHKDETRKQEQ-TEEQKKKSRRSRTTFSGDQLDVLEKEFDKTHYP-CVNTREDLANKTHLSEARVQVWFSNRRAKWRRHKKIGSLPHP
Spur_LOC574563      SSSLMSSSSPSSSSSFSMDDFVNMSLLDCHNAAAAAAAKEMSMAAAAA-----------------SAERKKLRRSRTTFTQNQLAVLESDFEKTHYP-CVNTREELATKTSLSEARVQVWFSNRRAKWRRHKKMPSTSIP
Cg_EKC38178         ---------------------------------------------EEEKEITSKDTCTPKEDEETETSTSKLRRNRTTFTSQQLDLLEQSFQKAHYP-GVQTREELATKTNLSEARVQVWFSNRRAKWRRSQRFTFLHQT
Ce_EYG-1            --------------------------SGALAPPVQSGGAGGPGVF-GNSGQISVDSNLNLSEDDSTLGANARRLSRSTFTNEQLQSLEEVFLRDPYP-SPNERADLVKRTNLPEARIQVWFSNRRAKWRKTNANGSDRDE
Crem_EYG-1          --------------------------SGALSLPVQGAGGALIGSGGGSSGQVSVESNPSLSEDDSTLGANARRLSRSTFSNEQLQSLEEVFLREPYP-SQTERADLVKRTGLPEARIQVWFSNRRAKWRKTAAN--DRDE
                                                                                          1...:...10...:...20...:. ..30...:...40...:...50...:...60
```

HOMEODOMAIN

```
                    841
Ce_NPAX-3           --------------------------------------------------------------------------------------------------------------------------------------------
Ce_NPAX-4           --------------------------------------------------------------------------------------------------------------------------------------------
Acey_NPAX-4         --------------------------------------------------------------------------------------------------------------------------------------------
Ce_NPAX-1           --------------------------------------------------------------------------------------------------------------------------------------------
Ce_NPAX-2           --------------------------------------------------------------------------------------------------------------------------------------------
Dm_Poxn             --------------------------------------------------------------------------------------------------------------------------------------------
Md_Poxn             --------------------------------------------------------------------------------------------------------------------------------------------
Cc_Poxn             --------------------------------------------------------------------------------------------------------------------------------------------
Sk_Poxn             NSYAHHRTFSRPDLPITTCGPVLMPPTMLHFHPPVTTASFTRPPHYQFPACNMSSDWGYTQLTAQHKH------------------------------------------------------------------------
Ac_Poxn             DDTCSVAGCSERSESPVDIDVVAEESEDCAESIKSNDTTNKLETIIRSSTPTLKRSAEVDTRPYYRRINSPSPTVTSPNSPTRNTVTSSELVMATESPS-TLTPVAATLKSPHVSTNSASGHPALLNPPALAVSTVLSGR
Cg_EKC37735         YFSTCLTNISSKAAREHSPSALYSSFYHPYARFGATSRGHDVTPYVIPRLCMLPDGLSAFSNSLLTHTDSKASTYSRSFDSHLLQSSLHMK-------------------------------------------------
Hs_PAX1             GSLPAPAARPRTPSVAYTDCPSRPRPPRGSSPRTRARRERQADPGAQVCAAAPAIGTGRIGGLAEEEASAGPRGARPASPQAQPCLWPDPPHFLYWSGFLGFSELGF---------------------------------
Mm_Pax1             GTDRKPPSPGGKATDALGSLHGLPIPASTS--------------------------------------------------------------------------------------------------------------
Hs_PAX9             GS-------HSVTASAL---------------------------------------------------------------------------------------------------------------------------
Mm_Pax9             GS-------HSVTASAL---------------------------------------------------------------------------------------------------------------------------
Eb_Pax1/9           GS-----TSPEWPGFAYGACPTGPWQNCPARVLMQAAYDLQSDGGKDFCS------------------------------------------------------------------------------------------
Sk_Pax1             HHPNPTYVPTYGATTAMSSSLGHSVWHSGNGFPCSSGTNLNSDVLRPAALAYKPVVTTSSCSVVENASHQHTNSMALIQSTQSS--------------------------------------------------------
Cg_EKC39574         --------------------------------------------------------------------------------------------------------------------------------------------
Dm_Poxm             --------------------------------------------------------------------------------------------------------------------------------------------
Dm_Sv               SGNDTSMLYDSITTISQTQSSIYTPAIGPSIGTGSLTPLVPISMHEMKLSAN-----SIQEQTVPPFYTALAFDGNYTSMTSLENCSSLVGQEHIVMPESSDSNTLCPISTRIVPDITETNSTRVKEPLTNSDGCSEDNN
Sk_Pax2             APVSMIQDVKPSIDRMVHSPPQQPIPSPYQTGSCIGGNPSPQSNLKMEATTSGPTTLTVLQPVASNNNMSGYMTDRPSLPQLPPANPPSGFQVGQYSGQSRDMSG----IHGYPPAAAAQ--SSYPSSTMSGLVPPLVLP
Cg_EKC36239         SNFVTSLGHQLGTEISVNAQTQDMKPIVVAVVTESQPPAQQEQFQEATNNTPTSSSTPTINNNNNTPPPKAGSPGSGGGANNLTELKPVQPTMSQSYTPLPSFSGQFSSQASSYNGSHATFPSQAVVGTVVQPLVISSGS
Hs_PAX2             LGSNVSGTQTYPVV------------------------------------------------------------------------------------TGRDM--ASTTLPGYPPHVPPTGQGSYPTSTLAGMVP-----
Mm_Pax2             LGSNVSGTQTYPVV------------------------------------------------------------------------------------TGRDM--TSTTLPGYPPHVPPTGQGSYPTSTLAGMVP-----
Hs_PAX5             IGSSVPGPQSYPIV------------------------------------------------------------------------------------TGRDL--ASTTLPGYPPHVPPAGQGSYSAPTLTGMVP-----
Mm_Pax5             IGSSVPGPQSYPIV------------------------------------------------------------------------------------TGRDL--ASTTLPGYPPHVPPAGQGSYSAPTLTGMVP-----
Hs_PAX8             LGRNLSTHQTYPVVADPHSPFAIKQETPEVSSSSSTPSSLSSSAFLDLQQVGSG---------------------VPPFNAFPHAASVYGQFTGQALLSGREM--VGPTLPGYPPHIPTSGQGSYASSAIAGMVA-----
Mm_Pax8             LGRNLSTHQTYPVVADPHSPFAIKQETPELSSSSSTPSSLSSSAFLDLQQVGSGGPAGA---------------SVPPFNAFPHAASVYGQFTGQALLSGREM--VGPTLPGYPPHIPTSGQGSYASSAIAGMVA-----
Bf_Pax2/5/8         ---------------------------------PSISCSTTSVAMTDTAPHVPTG----------------------------HYPVAVPGLPQSAVTPGRDMRDMNSTLPGYPPHAPPSNLSGQTGYPSNTMATGLVPP
Hs_PAX3             ----FNH------------------LIPG--------GFPPTAMPTLPTYQLS---ETSYQPTSIPQAVSDPSSTV------------HRPQPLPPS--------------------------------TVHQSTIPSNP
Mm_Pax3             ----FNH------------------LIPG--------GFPPTAMPTLPTYQLS---ETSYQPTSIPQAVSDPSSTV------------HRPQPLPPS--------------------------------TVHQSTIPSNA
Hs_PAX7             ----FNH------------------LLPG--------GFPPTGMPTLPPYQLP---DSTYPTTTISQDGGSTV---------------HRPQPLPPS--------------------------------TMHQGGLAAAA
Mm_Pax7             ----FNH------------------LLPG--------GFPPTGMPTLPPYQLP---DSTYPTTTISQDGGSTV---------------HRPQPLPPS--------------------------------TMHQGGLAAAA
Cg_EKC41820         NSILHLPQGGASSYLLHETTPSYPLPSVSDTLWHRNSMTHHLQNFSSLKTNDSAYAGLMENYLSHASQMHNLTNPTDSLASNWCSPVTSSVSALAYQTTTNHFNPHG---------------------------------
Sk_LOC100371646     SPWYPMATTGSVMPVSAGPPCVLPDTMHSFPSQDFSFLPPSHPSRLYQNSFYTQYNPPTFSLPSMPDLSTGSKSVVSGPVHQHVYQGQPEYVQSSLCKNPIVMNRPIIPPVSKSPVCSLPMIVNHPMTPTVTNTPCQPFP
Dm_Gsb              ----FAPTSTSFGATPTTSAAPAPNMGMS--------LYSSQSWPSSGAYENH---AAYGGSV-------------------------ASMSPASST--------------------------------SGTSSAAHSPV
Dm_Gsbn             PMNS-----GSSNVG---------------VGVGLSGATAPLGYGPLGVGSMAGYSPA---PGTTATGAGMNDGV------------------HHAAHAPSS--------------------------------HHSAAT
Dm_Prd              GGAAASVSHVAASSSLPSVVSSVPSMAPLAMMPGS-------LDPATVYQQQYDFYGS---HANISVSAAAPMASSNLSPGITTTPPHHHQF-YNPSANTAS--------------------------------YIMPGE
Hs_PAX4             ----LPG------------------ASQG--------LTVPRVAPGI-----------------------------------------ISAQQSPG--------------------------------------------
Mm_Pax4             ----LPG------------------ASQD--------LTVPKNSPGI-----------------------------------------ISAQQSPG--------------------------------------------
Hs_PAX6             A---SNT---------------PSHIPISSSFST-SVYQP-IPQPTTPVSSF-TSGS--------------MLGRTD--------------------------------------------TALTNT-------------
Mm_Pax6             A---SNT---------------PSHIPISSSFST-SVYQP-IPQPTTPVSSF-TSGS--------------MLGRTD--------------------------------------------TALTNT-------------
Bf_Pax6             QDSDSSS---------------PSRIPISSSFSTATMYQP-IAPPSAPVMSR----------------------SSH--------------------------------------------AGLTDS-------------
Sk_Pax6             MSSAPTSSSHLPINSSFTNSVYQPIPQPTTPMVPRTADSYSALPPVPSFSMATPPNLSGPRDTSSYSCMIPPSARSYEHHLSLGGYSS----------------------------------------------------
Dr_Pax10            GSSSSCSQ---------------THTPLSTSFNSP-VYHSHHSNSSGSMH-----------------------SRSDSSLSGYSSLSVFSSMQSLPSQ------------------------------------------
Dm_Toy              ADTVDGSGRTSTAN-------NPSGTTASSSVATSNNSTPGIVNSAINVAER-TSSALVSNSLPEASNGPTVLGG-E--------------------------------------------ANTTHT-------------
Od_Pax6             TSAHSNSYYPSIKTEHSTGSSPISGYPAMASGIPNSASAVSDVVNPAAYSGMHSMTQN----------------------------------------------------------------------------------
Dm_Ey               PNSTGASATSSSTSATASLTDSPNSLSACSSLLSGSAGGPSV--------ST-INGLSSPSTLSTNVNAPTLGAGIDSSE--SPTPIPHIRPSCTSDNDNGRQSEDCRRVCSPCPLGVGGHQNTHHIQSNGHAQ------
Dpse_Ey             PNSTGTSGTSSSTSANASLTDSPNSLGACSSLLSGNC--PSG--------NT-INGLASPNTLSTPA-----VGGVESTD--SPTPTLHLRSVGNSDSAGGQPQEDGSDACSPGLAISGQHSTHHHLA-HAHAHAHSHPH
Cg_EKC31583         VANGATRLPINGGFTNGMYPTIHQQIGTMAADTYSSMPTMPSYTLSSNNIQANPACLQSSNASSYSCMIPEYAPRSYDPL----SLSNYPRPSCNPAAGMQSHMTHNNHPSAASTGLISPGVSVPIQV------------
Dm_Eyg              RSSPANPLHSQQS---------ND-------APAS-SPTPSNHSSASTSA---------PVAPVPPPQQPLPLCGDH--SPQGPPPSVLLHP-LHGPPGGHHHHHPLHLPTHPAALQLLSHYHQQQQQQQQQQHQQQQQQ
Md_Eyg              SSPTGNTSTAHYS---------QQSLETCSNPTST-SPTPSTNSNASTCPNDQSSSITQQSHSAPPSSDPSLLPGHQAGQPVQPQPSLQAHM------SNMHSH--HGDPQSAAAALLLNHYHQQHQQLAKGQSPNLAMP
Dm_Toe              DSPSTSSSP-----------------------------TPLVNP---------------VVSPVSPIPVPVPVAVPESGQQKQPYPYSTSNM-CNTSSSSSNS-QPCNT-INPGS-----KMSSKTSSV----SSNQH--
Am_Eyg              PPPPPPPPPPPLQQQQQ------------------------------------------------------------------------------------------------------------------QQQQQQQQQ
Cbir_X777_10953     PPPPPPPPQPQPQQQQQ-------------------------------------------------------------------------------------------------------------------------
Nvit_Eyg            PPHHPASGTSGVPATSSIPTVPAVAAGQ-----------------------------------------------------------------------------------------------------------PQQQQ
Apis_LOC100165522   GVAAASTVSGGSSGSAAGTPTPPVVSPVAGAIGGPVG-------------------------------------------------------------------------------------------------------
Sk_Eygone           SPVQPSYPLYKPIPLNEPASSTVHVSEEKAKTLIP---------------------------------------------------------------------------------------------------------
Spur_LOC574563      HQVSHAHLPRMPLSSASTSMAAVGLLSHPYYQFYS---------------------------------------------------------------------------------------------------------
Cg_EKC38178         TYPVLPAFPSISLADGVRF-------------------------------------------------------------------------------------------------------------------------
Ce_EYG-1            LRAERSETDDALSISSQSPSPGGGQETGSDEGKKKTITLFKPYE------------------------------------------------------------------------------------------------
Crem_EYG-1          SRAERSETDDAMSNFSQSPSPGGQGSNSEDGGKKKTVTLFKPYE------------------------------------------------------------------------------------------------
```

```
                         981
Ce_NPAX-3               --------------------------------------------------------------------------------------------------------------------------------------------
Ce_NPAX-4               --------------------------------------------------------------------------------------------------------------------------------------------
Acey_NPAX-4             --------------------------------------------------------------------------------------------------------------------------------------------
Ce_NPAX-1               --------------------------------------------------------------------------------------------------------------------------------------------
Ce_NPAX-2               --------------------------------------------------------------------------------------------------------------------------------------------
Dm_Poxn                 --------------------------------------------------------------------------------------------------------------------------------------------
Md_Poxn                 --------------------------------------------------------------------------------------------------------------------------------------------
Cc_Poxn                 --------------------------------------------------------------------------------------------------------------------------------------------
Sk_Poxn                 --------------------------------------------------------------------------------------------------------------------------------------------
Ac_Poxn                 HHHHHQSHNFLESKLAFL----NSYTLAAHRFPGLALTPDPRLCLSHPRFLPLLGQSVSPISLRSPPSSGIASVYPLQLQEQRCALSSVPPT------------------------------------------------
Cg_EKC37735             --------------------------------------------------------------------------------------------------------------------------------------------
Hs_PAX1                 --------------------------------------------------------------------------------------------------------------------------------------------
Mm_Pax1                 --------------------------------------------------------------------------------------------------------------------------------------------
Hs_PAX9                 --------------------------------------------------------------------------------------------------------------------------------------------
Mm_Pax9                 --------------------------------------------------------------------------------------------------------------------------------------------
Eb_Pax1/9               --------------------------------------------------------------------------------------------------------------------------------------------
Sk_Pax1                 --------------------------------------------------------------------------------------------------------------------------------------------
Cg_EKC39574             --------------------------------------------------------------------------------------------------------------------------------------------
Dm_Poxm                 --------------------------------------------------------------------------------------------------------------------------------------------
Dm_Sv                   -KEPEKSNSSQSSDHIASPHLHHIGEEQLRGNRRSNLNASTHPSSLIPLQPSGGSSLLNINPSENRSDVELNLSNNVGLYSTPTVLPSFNHYSAGCSSVVPGSDYAYNPAYTQYGGAYGSYGYGTGSGLINSSYYYESGQ
Sk_Pax2                 HA-------SASYSAREPTDYSTQFSSVPYTHAQYSSMSSMSSHYSDAWS--RYGS-TGLLSECQTYRHIDRQSHR----------------------------------------------------------------
Cg_EKC36239             YNPTNPPS----------GEYYNTAGTVPYTQYQ-----TAAYPADPAWT-MRYTNPTGILNTPYYYQTPVSGRNEPNTTVAASASPSKS--------------------------------------------------
Hs_PAX2                 -----------------GSEFSGNPYSHPQYTA-----------YNEAW---RFSN-PALLSSPYYYSAAPRGSAPAAAAAAYDRH------------------------------------------------------
Mm_Pax2                 -----------------GSEFSGNPYSHPQYTA-----------YNEAW---RFSN-PALLSSPYYYSAAPRGSAP--AAAAYDRH------------------------------------------------------
Hs_PAX5                 -----------------GSEFSGSPYSHPQYSS-----------YNDSW---RFPN-PGLLGSPYYYSAAARGAAPPAAATAYDRH------------------------------------------------------
Mm_Pax5                 -----------------GSEFSGSPYSHPQYSS-----------YNDSW---RFPN-PGLLGSPYYYSPAARGAAPPAAATAYDRH------------------------------------------------------
Hs_PAX8                 -----------------GSEYSGNAYGHTPYSS-----------YSEAW---RFPN-SSLLSSPYYYSSTSRPSAPPTTATAFDHL------------------------------------------------------
Mm_Pax8                 -----------------GSEYSGNAYSHTPYSS-----------YSEAW---RFPN-SSLLSSPYYYSSTSRPSAPPTSATAFDHL------------------------------------------------------
Bf_Pax2/5/8             IVLPSASNSYSSASTMSGSDYSSQFSGVPYTHAQY------SSHYNDAWNQMRYPT-PGILSSYPYYSSS-RGPATAAAAARQYST------------------------------------------------------
Hs_PAX3                 DSSSAYCLPSTRHGFSS---------------YTDSFV---PPSGPSNPMNPTIGNGLSPQVMGLL----TNHGGVPHQPQTDYALSP-----LTGGLEPTTTVSASCS--------QRLDHMKSLD-------------
Mm_Pax3                 DSSSAYCLPSTRHGFSS---------------YTDSFV---PPSGPSNPMNPTIGNGLSPQVMGLL----TNHGGVPHQPQTDYALSP-----LTGGLEPTTTVSASCS--------QRLEHMKNVD-------------
Hs_PAX7                 AAADTSSAYGARHSFSS---------------YSDSFM---NPAAPSNHMNP-VSNGLSPQVMSIL----GNPSAVPPQPQADFSISP-----LHGGLDSATSISASCS--------QRADSIKPGD-------------
Mm_Pax7                 AAADTSSAYGARHSFSS---------------YSDSFM---NPGAPSNHMNP-VSNGLSPQVMSIL----SNPSAVPPQPQADFSISP-----LHGGLDSASSISASCS--------QRADSIKPGD-------------
Cg_EKC41820             ----------------------------------------------------------------------------------------------------------------------YGDVTKSTLH------------
Sk_LOC100371646         SPPWVHTMPAGGQEPYQQVSQFSPKHGLYSSFM-----------------------------------------------------------------------------------------------------------
Dm_Gsb                  QTQAQQPGTGSEFMTST---------------YGV------GSSNATYPS---AAYSMPQTPATS------------------------------------------------------AEQLRSQF-------------
Dm_Gsbn                 AAAAAHHHTQMGGYDLVQSAAQ----------HG------------FPGGFAQPGHFGSQNYYHQDYSKLTIDD--FSKLTADSVSKISPSLHLSDNY--SKLEAPSNWSQAAYH----AAANYNAHVA----------
Dm_Prd                  NGNTTPTGNIIVSSYETQLGSV----------YGTE-----TETHQTMPRNESPNESVSSAFGQ------------LPPTPNSLSAVVSGAGVTSSSGANSGADPSQSLANASAGSEELSAALKVESVDLIAASQSQLYG
Hs_PAX4                 ----------------------------------SVPTAALPALEPLGPSCYQLCWAT----------------------------------------------------------------------------------
Mm_Pax4                 ----------------------------------SVPSAALPVLEPLSPSFCQLCCGT----------------------------------------------------------------------------------
Hs_PAX6                 -YSALPPM----PSFTMA-----NNLPM-QPPVP----SQ----TS--SYSCMLPTSPS---------------------------------------------------------------------------------
Mm_Pax6                 -YSALPPM----PSFTMA-----NNLPM-QPPVP----SQ----TS--SYSCMLPTSPS---------------------------------------------------------------------------------
Bf_Pax6                 -YSSLPPV----PSFSVP-----GNMAP-MPSMQQ--SRD----QT--SYSCMIPHSTA---------------------------------------------------------------------------------
Sk_Pax6                 ---------------------------------NSTHPIGQPPIQSHVSSACMTQTS-----------------------------------------------------------------------------------
Dr_Pax10                ---------------------------------------------STPTYSCMLPPSPSALP------------------------------------------------------------------------------
Dm_Toy                  SSESPPLQPAA-PRLPLNSGFN-----TMYSSIPQPIATMAENYNSS---------------------------------------------------------------------------------------------
Od_Pax6                 --------------------------------------------------------------------------------------------------------------------------------------------
Dm_Ey                   ---GHALVPAISPRLNFNSGSFG----AMYSNMHHTALSMSDSYGAVTPIPSFNHSAVGPLAPPSPIPQQGDLTPSSLYPCHMTLRPPPMAPAHHHIVPGDGGRPAGVGLGSGQSANLGASCSGSGYEVLSAYALPPPPM
Dpse_Ey                 THTHSHALPAISPRLNFNSGSFSSSMSAMYSNMHHPALSMGDSYGSITPMPSFSHSSVGPLAPPSPLSQQRDLTPPSLYPCHMTLRPPPIAGPHHHLSPNDAGNSANV-----HSSNLGASNNGTGYEVISSYGLAPPPL
Cg_EKC31583             --------------------------------------------------------------------------------------------------------------------------------------------
Dm_Eyg                  H------HQQQQQLSPSGCNPAANHLSMGGERSAFRSLVS----------------------------------------------------------------------------------------------------
Md_Eyg                  ATSFGVLGSESHQQLAAAIATGSPTLSMGGEHSAFRSLVS----------------------------------------------------------------------------------------------------
Dm_Toe                  -------MEEPAAAVATASPTASAPLSMGGENSAFRALPMTLPMPMT---------------------------------------------------------------------------------------------
Am_Eyg                  QQQQQPPQPHSASGIEVNRASSCSIGGMGGESSAFRVVVTNSSSCSSSS-------------------------------------------------------------------------------------------
Cbir_X777_10953         ------PHSATSAMEIGQRTSSCSIAGMGGESSAFRAVVTNSTSRDTNLT------------------------------------------------------------------------------------DRPSER
Nvit_Eyg                PSQSS-------------HSAGGGARLGSGEKSAFRTV------------------------------------------------------------------------------------------------------
Apis_LOC100165522       ---------GPDGVGGPPCPAAVARCLGGGENSAFRSLMPQQHLHRQLIEQSYAASESSEEINVTTDDEDDEGGAVGSGGVCGVGVIDEPMDDGDGSNNSSSYNHNRNNDNNNNDGSGGGGDGDIDDDDDDGKTSLTSPA
Sk_Eygone               -----------------------------SSRSAFTAAPSPKITSPASSPLVIVSSPSPSAMTSSASISFSRRP------------------------------------------------------------------
Spur_LOC574563          ------ALQRPESFKPIELG-GSKMLKSSRVTSAFQPVTSDMVRPSMNSSS-----------------------------------------------------------------------------------------
Cg_EKC38178             ---SSFQTDARFMNKPSDISFSEEHDVIGSKESAFRRLKYPEERKSV---------------------------------------------------------------------------------------------
Ce_EYG-1                --------------------------------------------------------------------------------------------------------------------------------------------
Crem_EYG-1              --------------------------------------------------------------------------------------------------------------------------------------------
```

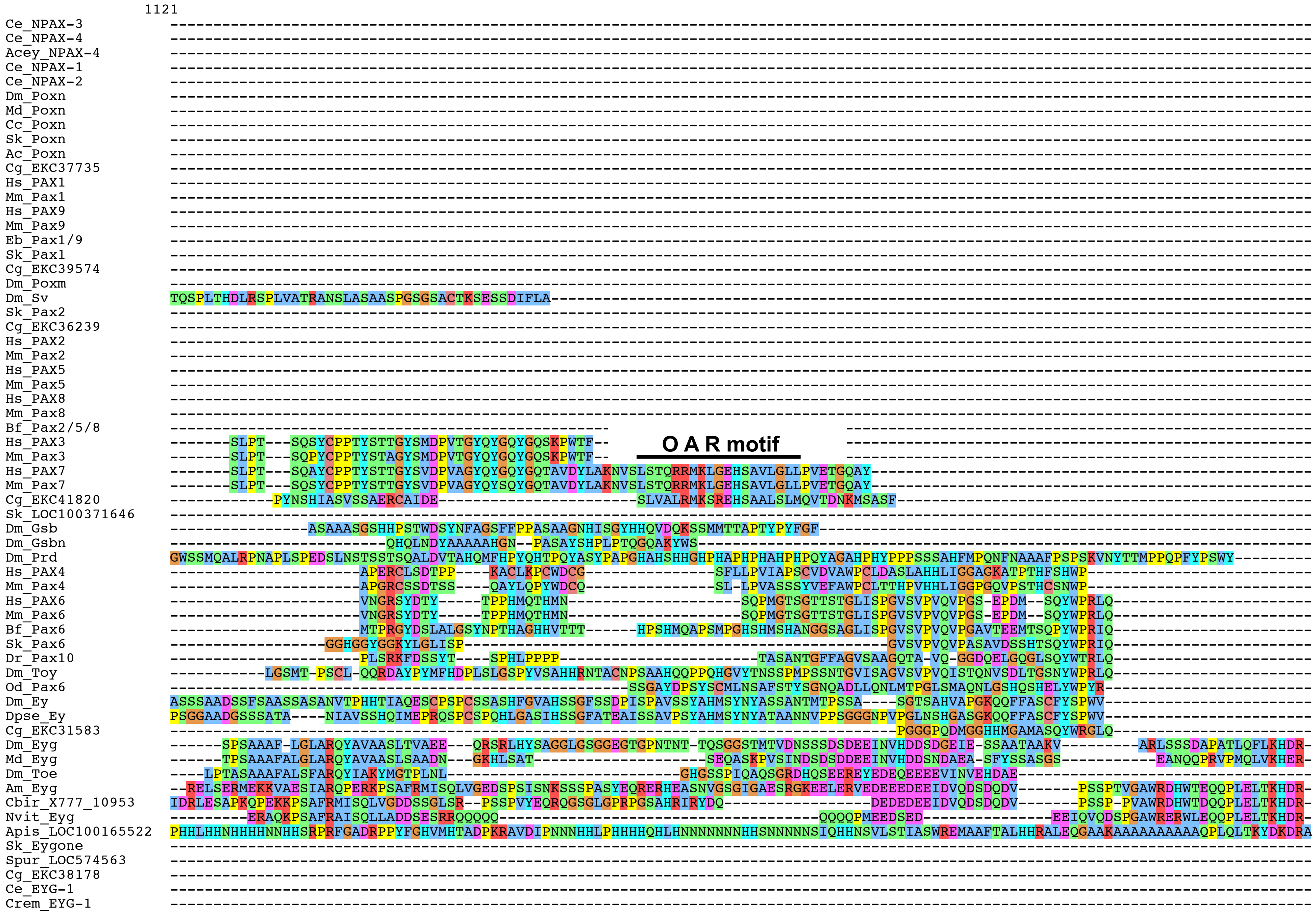

Homeodomain proteins: an update. Chromosoma, Bürglin, TR, and Affolter, M. Dept. of Biomedicine, University of Basel. email: thomas.buerglin@unibas.ch
